# Harnessing Neurogenic Potential of Glial Cell Types as an Emerging Strategy for Treating Epilepsy

**DOI:** 10.3390/ijms27209147

**Published:** 2026-10-15

**Authors:** Ekaterina Chesnokova, Irina Kotova, Anna Reznikova, Pavel Balaban, Natalia Bal

**Affiliations:** Laboratory of Cellular Neurobiology of Learning, Institute of Higher Nervous Activity and Neurophysiology of the Russian Academy of Sciences, Moscow 117485, Russia; laylay.96@mail.ru (I.K.); anchez.rez@gmail.com (A.R.); pmbalaban@gmail.com (P.B.); bal_nv@mail.ru (N.B.)

**Keywords:** epilepsy, reactive gliosis, astrocytes, neural stem cells, oligodendrocyte precursors, microglia, transdifferentiation, dedifferentiation, induced neurons

## Abstract

In every third case of epilepsy, anti-seizure drugs are ineffective, so the search for new therapies for this disease is ongoing. In recent years, new methods for generating cells with desired properties, as well as for the targeted expression of genes in specific cell types in situ within the brain, were designed. These methods allowed researchers to consider the idea of transforming resident glial cells into neurons in conditions like epilepsy; in such pathologies it may rescue both neuronal loss and gliosis. In this review, we describe epilepsy-associated pathological changes in glial cells, the neurogenic potential of different glial types (their similarities to neural stem cells and their inherent limited ability to produce neurons in response to brain damage), and existing methods of neuronal transdifferentiation starting with different cell types with varying proximity in lineage distance to neurons. Based on overlaps between these topics, we make conclusions about the applicability of glia-to-neuron conversion in treating epilepsy and the most important challenges that should be considered in designing experiments and clinical trials for this strategy.

## 1. Introduction

Epilepsy is one of the most common neurological disorders, affecting about 50 million people worldwide [1]. It is characterized by increased predisposition to seizures. A seizure is defined as a transient behavioral change caused by abnormal excessive or synchronous neuronal activity in the brain, with diverse symptoms like loss of awareness, specific sensations or uncontrolled movements [2]. The factors that may trigger epileptogenesis are also very diverse; there are multiple genes associated with epilepsy, and seizures may result from various brain insults and injuries, like trauma, infection, tumors, stroke and others [2,3]. Importantly, the latent period between the initial brain damage and the start of chronic, unprovoked seizures may be very long, taking years in some cases [4].

Temporal lobe epilepsy (TLE) is a type of epilepsy that attracts particular attention amongst researchers. It is the most common form of focal-onset epilepsy to be referred for surgical intervention due to being refractory to antiepileptic drugs. The temporal lobe is considered the most epileptogenic region within the brain [5].

Seizures are a consequence of disrupted excitatory/inhibitory balance in neuronal circuits. The usual mechanisms of this on the cellular level are an altered function of ion channels and/or an imbalance in glutamatergic and GABAergic synaptic transmission [6,7,8,9]. Notably, to trigger seizures, a mutation in only a fraction of neurons may be enough. A targeted somatic mutation (*Pten* deletion) was sufficient to cause epilepsy in mice when mutant cells comprised as few as 9% of the whole dentate granule neuron population [10]. There is also evidence of alterations in the topologic properties of the whole brain white matter network in epilepsy [11,12,13]. This is why epilepsy research has been mostly focused on the pathological changes in neurons, and why existing anti-seizure drugs act on neurons. However, these drugs only provide seizure control, while not eliminating the underlying pathological changes in the brain. Moreover, about a third of epilepsy cases are drug-resistant [14,15,16,17]. In recent years, the role of glia in this disease has become increasingly recognized, with multiple mechanisms by which glia contribute to epileptogenesis being revealed. In 2015, International League Against Epilepsy (ILAE) and International Bureau for Epilepsy (IBE) included assessing the therapeutic potential of targeting glial cell function in the list of priorities for epilepsy research [18].

The four major glial cell types are astrocytes, oligodendrocytes, microglia and oligodendrocyte precursors (OPCs) [19]. In this review, we will mostly describe the pathological changes in astrocytes and their contribution to epileptogenesis. We will also discuss other types of glial cells in this context where relevant. Neural stem cells (NSCs) are not usually classified as glia, but they share a lot of similarities with embryonic radial glia and astrocytes, to the point where some researchers consider them a special type of astrocytes. So we discuss NSCs and their role in epilepsy as well.

**Figure 1:**
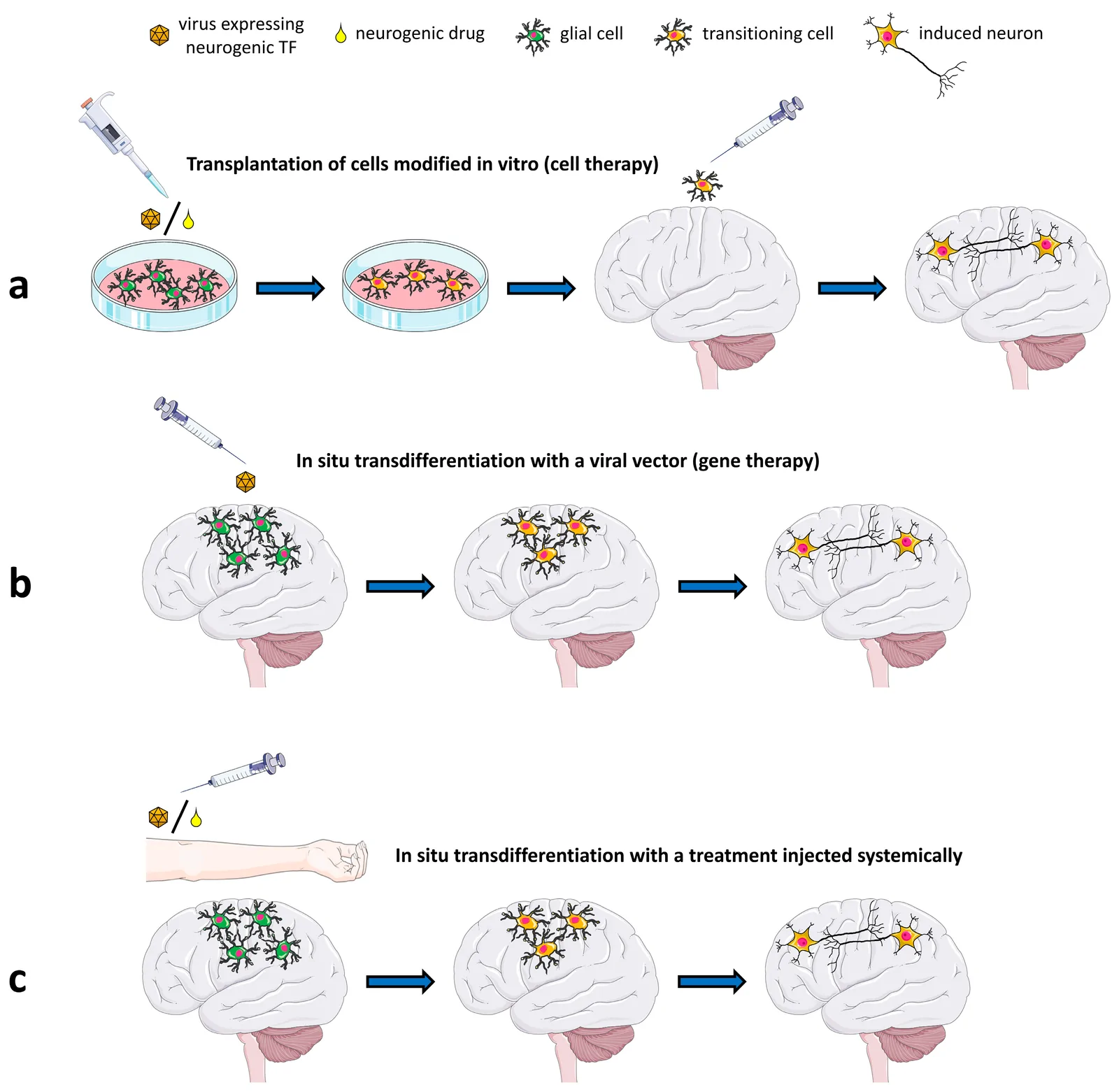
An illustration of different methods and delivery strategies proposed for treating neuropathologies by glia-to-neuron transdifferentiation and tested in animal models. (**a**) Cell therapy. Cultured glial cells (that may be obtained by different methods from the patient or from a donor) are treated in vitro by neurogenic factors (drugs or viruses expressing TFs). After the treatment, the cells are transplanted to the damaged brain region where they undergo neuronal transdifferentiation. (**b**) Gene therapy. A virus expressing a neurogenic TF is injected to the damaged brain region. Resident glial cells infected by the virus undergo neuronal transdifferentiation. (**c**) Systemic treatments. Small-molecule drugs and some viral vectors are able to cross the blood–brain barrier (BBB) after a systemic injection. In this case, resident glial cells undergo neuronal transdifferentiation without the need to perform injections into the brain. It is expected that glial cells in the damaged brain region would be more responsive to the neurogenic treatment than glial cells in other regions. Any strategy of glia-to-neuron transdifferentiation produces fewer induced neurons (iNs) compared to the number of treated glial cells; some cells do not complete the transdifferentiation process, they undergo cell death or revert to their glial identity. In most cases in animal models, the starting cell type is astrocytes. However, some experiments used OPCs or microglia as the starting cell type. In addition, resident NSCs may also respond to the neurogenic treatment, and in some cases NSCs may be confused with astrocytes.

In recent years, the progress of methods allowing researchers to create cells with desired properties has made it possible to consider epilepsy treatments where transformed cells would replace the lost neurons, especially GABAergic neurons. Different cell types may be used as the starting material. Progress has been made with stem cell therapy in epilepsy [20,21,22,23,24]. However, this requires a source of stem cells and then an injection of the manipulated cells into the brain. A recently proposed alternative approach, which is the focus of the present review, is to use resident glial cells in the brain to transform them into neurons. If the transformation happens in situ, possible complications of cell transplantation would be avoided, and even an injection into the brain may be avoided if the transformation factor is given systemically. This is an ideal scenario; glia-to-neuron transformation protocols that are currently researched employ different ways of manipulating cells in vitro or in vivo. In some studies, glial cells are manipulated in vitro and then injected into the brain [25,26], which fits the classic definition of cell therapy. The most common approach in current studies is transdifferentiation in situ induced by a viral vector expressing a neurogenic transcription factor (TF); formally it should be called gene therapy, and the term “autologous form of cell therapy” has also been used for this [27]. The virus is usually injected directly into the target brain region; however, for some vectors, intravenous (i.v.) delivery was shown to also be effective [28,29,30]. Another way to achieve glia-to-neuron transformation is gene expression downregulation by oligonucleotides that may be injected intracerebroventricularly (i.c.v.) [31]. Some authors also propose drug-induced cell transdifferentiation in situ; the drug in this case may be injected systemically [32]. Different approaches to glia-to-neuron transdifferentiation therapy are shown in Figure 1.

The idea of treating epilepsy with these methods is still new, and so far there are few published experimental papers wherein both epilepsy models and glia-to-neuron conversion have been used. Thus, our review covers mostly indirect evidence that may be used as arguments to continue research into transdifferentiation-based epilepsy treatments, as well as to outline possible problems that may be related to this approach. First, to provide the description of the cells that would be the subjects of transdifferentiation specifically in the epileptic brain, we list epilepsy-associated changes in the types of glial cells known to be able to produce neurons (at least in vitro). We describe changes of astrocytes and microglia (Section 2.1, Section 2.2, Section 2.3 and Section 2.4), NSCs (Section 2.5) and OPCs (Section 2.6) that occur in the epileptic brain. Second, we describe the neurogenic potential of astrocytes and OPCs and injury-related neurogenesis in particular (Section 3.1 and Section 3.2); this theme is relevant for both neuropathologies and neuronal transdifferentiation, since artificial transdifferentiation exploits inherent neurogenic programs of the treated cells, the same programs that may be activated by brain lesions. Third, we describe processes happening in cells undergoing neuronal transdifferentiation (Section 4.1) and then catalog existing methods of this transdifferentiation (starting with different cell types) and application of these methods in models of different neuropathologies (Section 4.2, Section 4.3, Section 4.4, Section 4.5, Section 4.6, Section 4.7, Section 4.8, Section 4.9, Section 4.10 and Section 5, with Section 5 being focused on epilepsy models). Based on overlaps between these three main topics, we make conclusions about the specific properties of glia in the epileptic brain that, hypothetically, may be exploited for direct neuronal conversion, as well as the most important challenges and possible complications of this strategy that should be considered in designing experiments and clinical trials (Section 6 and Section 7).

## 2. Reactive Gliosis and Its Features Associated with Epilepsy; The Role of Glia in Epileptogenesis

### 2.1. Reactive Astrocytes; Heterogeneity of Reactive Astrogliosis

The term “gliosis” refers to a spectrum of changes in glial cells, particularly astrocytes and microglia, that occur universally in response to diverse forms of central nervous system (CNS) insults. The most common mechanism by which different injuries trigger reactive gliosis is through the changes associated with BBB damage. The increased permeability of the BBB promotes the extravasation of serum albumin, peripheral immune cells and chemokines into the brain parenchyma, where they interact with glia and induce gliosis [4]. Notably, a focal brain injury may quickly trigger gliosis in brain areas remote from the site of damage, even in the other hemisphere [33]. In neurodegenerative disorders such as Alzheimer’s disease (AD), Huntington’s disease (HD), amyotrophic lateral sclerosis or Parkinson’s disease (PD), there is no immediate BBB damage, and other mechanisms triggering reactive gliosis may be involved. In AD, reactive gliosis may be detected before the onset of symptoms [34].

Different grades of severity of reactive astrogliosis are recognized. In all cases, changes in gene expression (upregulation of *GFAP* expression in particular) and cell morphology (hypertrophy of astrocyte body and processes) are observed. In mild to moderate reactive astrogliosis, there are no other changes; if the triggering mechanism has been resolved, the process (at least, the morphology change) may be reversed. In severe diffuse reactive astrogliosis, astrocytes proliferate, and their processes start to overlap. This may cause the irreversible reorganization of tissue architecture. In severe reactive astrogliosis with compact glial scar formation, astrocyte proliferation and the overlapping of processes cause the formation of compact borders around areas of severe tissue damage; these glial scars include other cell types (different types of glia and fibromeningeal cells) and deposit collagenous extracellular matrix (ECM) with factors inhibiting axonal and cellular migration. These structural changes persist long after the triggering insult has been resolved [35,36]. Studies with nascent cell quantification showed that when there is no glial scar formation, reactive astrocyte proliferation is actually very limited, at least in neurodegenerative disorders. The increased density of GFAP+ cells in these cases is explained mostly by enhanced *GFAP* expression in existing astrocytes [34,37]. Bardehle et al. performed a live imaging of astrocyte responses to an acute injury using in vivo two-photon laser-scanning microscopy. GFAP was upregulated in almost all astrocytes close to the injury site, but most of them maintained their morphology for up to 4 weeks post-injury. Among the observed astrocytes, 42% entered a hypertrophic state; 10% formed elongated processes directed toward the lesion site (polarization), typically at 3–5 days post-injury; 16% proliferated (mostly astrocytes located close to blood vessels), typically at 5–7 days post-injury. Each proliferating astrocyte generated no more than two daughter cells. A larger injury size increased the proportion of polarized astrocytes, but not that of proliferating astrocytes. Importantly, no migration of astrocytes towards the lesion site was observed. Thus, the reactive astrocyte migration reported in scratch wound assays in vitro may be an artifact of culturing conditions [38].

Perisynaptic astrocyte processes (PAPs) are the finest astrocyte processes that govern astrocyte–synapse interactions. Neuronal activity and glutamate affect PAP plasticity through a Ca^2+^-dependent mechanism [39,40]. In astrogliosis, PAPs undergo structural remodeling; PAP changes are different for different underlying neuropathologies, with controversial reports in the case of epilepsy [16,41].

On the gene expression and function level, the heterogeneity of reactive astrogliosis is more complicated [42]. Sirko et al. demonstrated that reactive astrocytes triggered by different brain insults have different proliferation capacities; invasive injuries like a stab wound elicit their proliferation, while pathologies like amyloid plaques fail to do so [43] (see also Section 3.1.2). Zamanian et al. compared gene expression in reactive astrocytes in ischemic stroke or neuroinflammation induced by lipopolysaccharide in mice. The reactive astrocyte phenotype strongly depended on the type of inducing injury. At least half of the differentially expressed genes (DEGs) were specific to the injury type. In ischemia, reactive astrocytes had a molecular phenotype which suggested that they may be protective, while the phenotype of astrocytes activated by neuroinflammation suggested that they may be detrimental [44]. Later, the phenotype classification of reactive astrocytes was proposed: A1 for “detrimental” and A2 for “beneficial” types. A1 astrocytes show the upregulated expression of proteins known to be destructive to synapses. A2 astrocytes may show the upregulation of neurotrophic factors and secrete proteins that promote synaptogenesis. Astrocyte activation into A1 or A2 state (sometimes also called “polarization”, which in this case does not refer to the direction of growing processes) is determined by the signals from activated microglia [45]. In response to various brain insults, proinflammatory cytokines secreted by activated microglia, such as TNFα, IL1α, and C1q, induce A1 astrocytic phenotype. A1 astrocytes secrete neurotoxic factors, complement components like C3, and proinflammatory cytokines like CXCL10, which facilitate the recruitment of immune cells across the BBB into the CNS. Astrocyte monocultures treated with toxic agents like lipopolysaccharide or Aβ42 remain inactivated in the absence of microglia, highlighting the necessity of astrocyte and microglia crosstalk in this process [46]. The A1/A2 classification is not universally accepted, because it is oversimplified and fails to accurately represent the diversity of molecular expression patterns and functional changes in reactive astrocytes [37,42,46]. The range of possible functional changes in reactive astrocytes includes even their partial dedifferentiation, which is important to consider for transdifferentiation therapies (see Section 3.1.2).

Glial fibrillary acidic protein (GFAP) is the most commonly used cellular marker of reactive astrocytes [37], although there are other possible markers such as vimentin that are absent in resting (non-reactive) astrocytes [47]. Some genes upregulated in different types of reactive astrocytes may be used as more specific markers of these cells, like C3 for A1 astrocytes or STAT3 for astrocytes in a gliotic scar [37]. Burda et al. combined gene expression data from multiple studies to compare DEGs in reactive astrocytes (from different brain regions or spinal cord) across 15 different pathological CNS conditions in mice and humans (disorders, disorder models and aging). Over 12,300 genes that change expression level in astrocytes in at least one of these conditions were identified, with remarkable heterogeneity and low overlap across pathologies [48]. In regard to epilepsy, Chen et al. performed a subpopulation analysis of astrocytes in TLE patients using snRNA-seq data, and identified six distinct subclusters. Two subclusters expressed markers of “protective” astrocytes and one those of “detrimental” astrocytes. One subcluster had a high expression of genes associated with lipid transfer and metabolism [49] (see also Section 2.4.7).

Contributions of reactive astroglia with different phenotypes to the pathogenesis of different disorders are also different. In acute insults (like ischemia), the loss of homeostatic astroglial function leads to an acute loss of neurons. In chronic neuropathologies, a more subtle dysfunction of astrocytes over long periods contributes to epilepsy and to the progressive loss of neurons in neurodegenerative diseases [35].

### 2.2. Reactive Microglia

Microglia activation in response to brain damage includes complex changes in cell morphology and gene expression. Activated microglial cells develop an amoeboid shape and produce inflammatory mediators like cytokines and complement proteins. Short-term inflammation can promote neuroprotection by attracting microglia to phagocytose damaged cells, while long-lasting inflammation is linked to pathological consequences including epilepsy [50]. Activated microglia also proliferate in response to diverse brain lesions and neuropathologies [51].

Microglial cells can adopt several activation states; their simplest classification includes M1 (classical) and M2 (alternative) phenotypes. M1-polarized microglia express proinflammatory cytokines and key phagocytic markers. M2-polarized microglia are suggested to be reparative and neuroprotective, expressing anti-inflammatory cytokines and enzymes associated with cellular integrity and scaffolding. Microglia may express M1 and M2 markers simultaneously. M1/M2 marker expression varies in different neuropathologies [52]. In a more detailed classification, M2 is divided into different subtypes. A widely used marker for microglia is ionized calcium binding adaptor molecule-1 (IBA1), a protein involved in phagocytosis. This protein is present in all microglia; in activated microglia, IBA1 undergoes an increase in its expression level and a change in its localization within the cell. Its applicability as an activation marker is debated [53].

The presence of activated microglia is not only relevant for inflammation processes; both resting (with ramified morphology) and activated (amoeboid) microglia have functions in the intact brain, regulating neural circuit formation and maintenance [54] and the homeostasis of the neurogenic cascade [55]. In an early postnatal brain, microglia are mitotic, amoeboid in shape and phagocytically active; their transition to a ramified, nonphagocytic state happens later in development [56]. However, reactive microglia in a healthy brain has not been well studied; these cells are particularly hard to culture [37,57]. Microglia in culture rapidly lose their defining features [58].

### 2.3. Reactive Gliosis and Accompanying Histopathologies in Epilepsy

Since epilepsy is a heterogeneous disorder that may be triggered by diverse brain insults [2], it is safe to assume that in different cases of epilepsy, different types of gliosis may be found, from diffuse astrogliosis resembling that in neurodegenerative disorders to extensive astrocytic proliferation and glial scar formation (for example, associated with trauma). However, some specific traits of gliosis are more common in this disease.

Reactive astrogliosis is always visible in epilepsy surgery specimens [59]. It is an important argument for developing transdifferentiation-based therapies for this disease, since many researchers agree that reactive astrocytes are the preferred starting cell type; compared to resting astrocytes, reactive astrocytes may initiate neurogenic programs even without additional treatments, and are more susceptible to applied neurogenic factors (see Section 3.1.2, Section 4.4 and Section 6.1). In a massive histopathological study of resected brain specimens from 9523 epilepsy patients (71.9% of operations involved the temporal lobe), 36.4% of the patients had hippocampal sclerosis, 23.6% had tumors (mainly ganglioglioma), and 19.8% had malformations of cortical development. Glial scars were found in 4.9% of specimens. Notably, specimens classified as “no lesions” (7.7% of patients) had nonspecific reactive gliosis as the only histopathologic abnormality in the neocortex, white matter, or hippocampus [60]. In the neocortex of patients with epilepsy, astrocytes are typically activated at the BBB or the blood–cerebrospinal fluid (CSF) barrier (regarding the theme of the present review, we may speculate that such a location of reactive astrocytes is favorable for transdifferentiation treatments given systemically). In subcortical white matter, fibrillary gliosis can be seen. It is difficult to identify single astrocytes in this condition because they are covered by a dense meshwork of spider-like ramifying processes. An excess of heterotopic neurons in white matter can be seen in many epilepsy surgery specimens. White matter angiopathy and inflammatory cellular infiltrates are also frequently found [59]. In TLE brain samples, an increased number of GFAP+ reactive astrocytes in the cornus ammonis 1 (CA1) and cornus ammonis 3 (CA3) regions of the hippocampus is observed [5,61]. Increased numbers of IBA1+ activated microglial cells are observed in post-mortem brain tissue from people with different forms of epilepsy [62]. In hippocampal tissue surgically resected from patients treated for TLE with hippocampal sclerosis (HS-TLE), the excessive activated microglia in CA1 and CA3 was identified by immunolabeling for HLA-DR (a class II major histocompatibility antigen) [63]. The number of IBA1+ reactive microglial cells in CA1 and CA3 is also increased in animal models of TLE [64,65]; the degree of microgliosis within CA1 and CA3 corresponds to the level of neuronal loss and seizure severity [64]. It is suggested that microglia are the first cell type to become activated by a seizure insult. After this, microglia propagate seizure-induced inflammatory responses and contribute to epileptogenesis [66]. It should also be noted that in specific types of epilepsy [67,68] and in some animal epilepsy models [69], demyelination or hypermyelination may be observed. At the same time, demyelinating diseases increase the risk of developing epilepsy [67,68].

Hippocampal sclerosis (HS) is a histopathology particularly associated with mesial temporal lobe epilepsy (MTLE), but it may also be present in other epilepsy syndromes and sometimes in other neuropathologies. Despite having distinct diagnostic criteria, HS in epilepsy is heterogeneous in many aspects, including patterns of neuronal loss and responses to treatments [70]. HS is diagnosed in hippocampus samples by neuronal loss and chronic fibrillary gliosis centered on the pyramidal cell layer. The pathologic changes of HS include granule cell dispersion, mossy fiber sprouting, and alterations to interneurons. A more extensive sclerosis of adjacent structures in the medial temporal lobe, including the amygdala and parahippocampal gyrus, may also be observed [71]. In sclerotic hippocampi, in addition to the loss of more than 50% of neurons, the glial density is increased by ~80% compared to nonsclerotic hippocampi of TLE patients and normal autopsy controls [61]. We may suggest that this may be exploited in glia-to-neuron transdifferentiation therapies, especially considering some results in conditions with glial scars (see Section 6.1). In HS, selective neuronal death in hippocampal regions CA1, CA3, and CA4 is observed, while dentate gyrus (DG) granule cells and CA2 neurons survive. Granule cell dispersion means the migration of cell bodies of granule cells. Mossy fiber sprouting means that granule cell axons (mossy fibers) lose their target cells in CA3 and CA4 and sprout to other regions. However, the sprouting may happen independently of the loss of target neurons [72]. It is the most typical example of pathological neuronal circuit remodeling in epilepsy [14]. In the context of transdifferentiation therapy, such pathological circuit remodeling may present an obstacle for the correct integration of iNs (see Section 2.5 and Section 6.5). Granule cell dispersion and mossy fiber sprouting are associated with abnormalities in adult hippocampal neurogenesis [73,74] (see also Section 2.5). Both acute and chronic seizure burden correlate with the degree of gliosis in HS [75]. Depth electrode recordings have shown that seizures originate in the sclerotic hippocampus, and brain microdialysis studies have demonstrated that the extracellular concentration of glutamate rises in the sclerotic hippocampus just before a seizure. A surgical removal of the sclerotic hippocampus results in excellent seizure control in ~85% of patients [76].

Mathern et al. compared case histories and histopathological data from hippocampal specimens of 572 TLE patients and 73 patients with extra-temporal seizures. HS was strongly associated with an initial precipitating injury (IPI) in both groups. IPI usually happened at an early age and may or may not involve a seizure. The authors propose that HS is most likely a consequence of IPI and a cause of TLE, while hippocampal neuron loss may be a consequence of TLE [77].

### 2.4. Pathological Changes of Astroglia and Microglia in Epilepsy on the Molecular Level: Mechanisms by Which Glial Cells May Promote Epileptogenesis

Reactive astrogliosis is a hallmark of the epileptic focus. Astrogliosis may be not just a consequence, but also a cause, of seizures. Acquired epilepsies result from brain insults like stroke, traumatic brain injury (TBI) or infections; these conditions trigger astrogliosis and may cause a formation of a glial scar. In some cases, a surgical removal of the glial scar alleviates the seizures [78]. In TBI-associated epilepsy, gliosis precedes the development of epilepsy and thus could be causative [4,79,80]. Seizures are one of the most common symptoms of Alexander disease, caused by heterozygous mutations in the *GFAP* gene. A special case of seizures caused by glia dysfunction is glioma, an astrocyte-derived malignant tumor [4]. Moreover, some experiments with animals show that artificially induced gliosis may be sufficient to cause neuronal hyperexcitability or seizures [4,81,82]. Rat strains commonly used as models of the absence epilepsy (WAG/Rij and GAERS) show increased numbers of GFAP+ astrocytes and increased GFAP protein expression levels in multiple brain regions [83].

The reactivity of astrocytes in epilepsy is accompanied by dynamic transcriptomic and molecular changes, reflecting complex combinations of compensatory and detrimental phenotypes. In astrocytes from the same brain area (sometimes even in the same cell), inflammatory and oxidative stress mediators may coexist with molecules participating in anti-inflammatory and detoxification mechanisms [15]. Studies of brain tissue from MTLE patients identified changes in multiple astrocytic channels, receptors and enzymes. GFAP, connexin 43, voltage-gated Na^+^ and Ca^2+^ channels, and GABA transporter GAT3 are upregulated; aquaporin 4 (AQP4) shows upregulation and mislocalization. Proteins with decreased expression in MTLE astrocytes are glutamate transporters EAAT1 (GLAST1) and GLT1, inwardly-rectifying K^+^ channels K_ir_4.1 and glutamine synthetase [84]. In a recent transcriptomics study, Li et al. checked expression levels of 1168 human astrocyte-specific genes in TLE datasets. In TLE samples, 122 of these genes were upregulated. A functional enrichment analysis of these genes identified multiple terms, including “cell morphogenesis”, “actin filament-based process” and “actin cytoskeleton”, suggesting a structural remodeling of astrocytes. Many genes were enriched in the PAP-related subcategories; genes encoding Ca^2+^-binding proteins were also upregulated, likely related to Ca^2+^-dependent PAP remodeling. Multiple upregulated genes in the “plasticity” category were enriched in the “ECM” component. Nine hub epilepsy-associated astrocyte-specific genes were identified in silico using a protein–protein interaction network tool: *YAP1*, *AQP4*, *GJA1*, *NOTCH2*, *GFAP*, *ERBB2*, *ITGAV*, *LRP1*, and *ABCA1*. The upregulation of all these genes in epilepsy was validated in the epileptic rat hippocampus (pilocarpine model) by RT-qPCR. Out of nine hub genes, their roles in epilepsy have been extensively investigated previously only for *AQP4*, *GJA1*, and *GFAP*. Interestingly, three of the hub genes (*ITGAV*, *LRP1*, *ABCA1*) are associated with astrocyte-mediated phagocytosis. For five of the hub genes, expression levels positively correlated with seizure frequency documented in the dataset [16].

#### 2.4.1. Neuroinflammation

The role of neuroinflammation in neuropathologies, including epilepsy, is a vast area of research. We will limit this section to a short summary, with references to reviews where molecular cascades involved in neuroinflammation are described in detail.

In neuroinflammation, the contribution of microglia is essential, but changes in astrocytes are tightly connected to those in microglia. Microglia are usually the first cell type to respond to a CNS insult; however, in some pathologies, the changes in astrocytes may happen first. By releasing signaling molecules, microglia and astrocytes establish a complex bidirectional communication and modulate each others’ functions during the CNS response to damage [45,66]. The secreted molecules involved in microglia–astrocyte crosstalk in neuroinflammation during epilepsy include proinflammatory cytokines (IL1α, TNFα, HMGB1, CXCL10), complement components (C1q, C3), antibacterial proteins (lipocalin 2) and others. The intracellular cascades they activate after binding their receptors include MAPK (ERK, JNK and p38 branches), JAK-STAT3, IKK/NF-kB and other signaling pathways [66].

Dysregulation in the glial immune-inflammatory function is a common factor that contributes to neuronal hyperexcitability and the generation of seizures. On the other hand, acute seizures cause neuronal injury, which releases ATP, glutamate, and reactive oxygen species (ROS) that activate microglia and astrocytes. Thus, seizures and neuroinflammation form a vicious positive feedback loop. Neuroinflammation itself is a self-supporting process: multiple signaling cascades involved in inflammation converge in cell death by pyroptosis, which itself is highly pro-inflammatory since dying cells release damage-associated molecular patterns (DAMPs) that, in turn, activate receptors triggering inflammatory responses in neighboring cells; some DAMPs, like IL1α and HMGB1, are also classified as pro-inflammatory cytokines [66,85]. Other forms of cell death are also involved in the pathogenesis of epilepsy. Ferroptosis is an important neuronal death mechanism in TLE. The mitochondrion is a key organelle involved in ferroptosis, and mitochondrial damage in neurons disrupts energy metabolism, which may affect excitatory/inhibitory balance in the brain, increasing the susceptibility to seizures [86]. A recent study by Liang et al. demonstrated that in pentylenetetrazole (PTZ)-induced epilepsy in mice, neuronal death by ferroptosis increases susceptibility to seizures; ferroptosis inhibitors decreased seizure score and prevented neuronal damage. Ferroptosis correlated with neuroinflammation, A1 astrocyte activation and the level of cytokine CXCL10 [87]. We should also note that oxidative stress triggering ferroptosis is an important factor limiting the survival of glial cells undergoing neuronal transdifferentiation (see Section 4.1). While the neuroinflammation/seizure feedback loop involves multiple types of cells, there are some direct confirmations of the specific role of astrocytes in this process. Experiments with the kainic acid (KA) epilepsy model in mice demonstrated that artificial seizure induction triggers an overexpression of SerpinA3N in astrocytes, which activates the NF-κB signaling pathway and works as a pro-inflammatory factor; this, in turn, promotes further seizure aggravation [88].

Some cytokines usually associated with inflammation, like IL1β, TNFα and IL6, have neuromodulatory properties and are involved in normal synaptic transmission and plasticity. In increased concentrations (in inflammatory conditions), these molecules can induce network hyperexcitability and reduce seizure threshold [89]. Moreover, these cytokines also affect astrocyte properties important for neuronal excitability, like astrocytic glutamate uptake [90,91] or glutamate release [92].

Complement components C1q and C3 play important roles in neuroinflammation [46]. At the same time, the complement-dependent microglial phagocytosis of synapses is a core mechanism for synapse elimination in the developing brain. Complement components are expressed by CNS cells; in mice, microglia are the predominant source of C1q, and astrocytes, C3. Synaptic pruning starts with C1q and C3 binding to synapses destined for elimination. The C3 receptor on microglia recognizes C3-tagged synapses and triggers their phagocytosis. Disruptions of this process, including underpruning and overpruning, are associated with neuropathologies that may be caused by circuitry miswiring, including epilepsy. Elevated levels of C1q and C3 have been shown in brain samples resected from patients with refractory epilepsy. Additionally, mutations in some complement inhibitor genes have been associated with epilepsy [93]. These observations were corroborated in a rat model of status epilepticus (SE) and acquired TLE; it was demonstrated that C1q, C3, and iC3b (a product of C3 cleavage) levels are increased in the hippocampus up to 5 weeks post-SE, and greater iC3b levels correlate with higher seizure frequency [94]. However, there are some arguments that altered microglial phagocytosis may also be anti-epileptogenic (reviewed in [50]). One example is the mouse strain with the *Pten* mutation; these mice have both heightened neuronal excitation and functionally enhanced microglia. An analysis of different brain regions showed that neuronal overexcitation is normalized, apparently by synapse phagocytosis, in the hippocampus but not the cortex of these mice [95].

Drugs that had been used to reduce microglia activation in animal models of epilepsy and alleviated seizures include cyclooxygenase 2 (COX2) inhibitor celecoxib [96] and P2X7 receptor antagonist JNJ-47965567 [97]. More importantly, some anti-inflammation drugs, including IL1β synthesis inhibitor VX09-765-401, COX1/COX2 inhibitor aspirin and IL6 receptor inhibitor tocilizumab, have shown promising results for some types of epilepsy in clinical trials. There are also multiple case reports describing seizure reduction by such drugs, including IL1 receptor antagonist anakinra and microglia activation inhibitor minocycline. The efficacy of these drugs against seizures was confirmed, even though there is no direct evidence that their anti-inflammatory action is the main mechanism here (reviewed in [85]). Conversely, anti-seizure drugs may show anti-inflammatory effects. A number of anti-seizure drugs, including sodium channel blockers cenobamate and GS967, reduced the expression of inflammation markers like IFNβ, TNFα or CXCL1 in cultured murine microglia [98]. Thus, anti-seizure and anti-inflammatory mechanisms are tightly connected, and anti-seizure properties may be found for other anti-inflammatory drugs (and vice versa) in the future. This is important for two different aspects of neuroregeneration therapies in epilepsy, including glia-to-neuron transdifferentiation. On one hand, anti-inflammatory treatment would increase the survival of preexisting neurons and aid the formation of new neurons by promoting the survival of NSCs that are vulnerable to an inflammatory microenvironment (see Section 2.5). We may speculate that reducing inflammation would be beneficial not only for NSCs in the canonical neurogenic niches, but also for parenchymal neurogenic cells that become activated upon brain injury (the classification of these cells as astrocytes or NSCs is unclear; see Section 2.5, Section 3.1.1 and Section 3.1.2). In particular, anti-inflammatory drugs with mechanisms involving the reduction of oxidative stress would promote the survival of transitioning cells if glia-to-neuron transdifferentiation is used (see Section 4.1). However, on the other hand, reducing inflammation may hinder specifically astrocyte-to-neuron conversion therapies by decreasing the number of reactive astrocytes that are more susceptible to reprogramming compared to resting astrocytes (see Section 4.4 and Section 6.1).

#### 2.4.2. Other Changes in Microglia in the Epileptic Brain

In animal models of epilepsy, the expression of M1 and M2 markers differs between models; however, in both pilocarpine and kainate models, M1 markers (including IL1β, CD16, CD11b) are upregulated shortly after the SE induction, while microglial activation is reduced mostly to control levels in the early chronic phase [52]. In at least one genetic epilepsy model, Cstb^−/−^ mice, the proportions of both M1 and M2 microglia are elevated; moreover, in this mouse strain, microglial activation precedes astroglial activation, neuronal loss, and the onset of myoclonus. The microglia of Cstb^−/−^ mice mostly express M2 markers at postnatal day 14 (P14), but shift towards M1 type at P30, the age associated with the onset of myoclonus [99].

Pascual et al. reported that the activation of microglia occurring at the earliest step of inflammation can modulate the activity of excitatory synapses. The activation of microglia involves a rapid production and release of ATP; extracellular ATP binds with P2Y1R receptors on astrocytes and triggers the astrocytic release of glutamate and more ATP. This glutamate activates neuronal group I mGluR receptors, which increases excitatory postsynaptic current frequency. If the balance between excitation and inhibition is impaired (like in genetic models of epilepsy), this microglial modulation of synaptic activity could be sufficient to increase the probability of epileptiform waves [100].

Zhang et al. found that the increased seizure susceptibility following acute alcohol exposure in mice is a result of microglial activation. The proposed mechanism is the dysregulation of synapse formation supervised by microglia. Unexpectedly, in the hippocampal CA1 area of alcohol-treated mice, the number of GAD67+ GABAergic neurons was increased, while the expression of CaMKII (glutamatergic neuron marker) decreased. The staining of synapses showed an increased number of puncta positive for vesicular GABA transporter (VGAT) and a decreased number of puncta positive for vesicular glutamate transporter 1 (VGlut1). Inhibitors of microglia activation blocked alcohol-dependent changes in seizure susceptibility and the number of excitatory and inhibitory synapses. Notably, alcohol did not induce significant changes in astrocyte reactivity assessed by GFAP and complement C3 levels [101].

#### 2.4.3. Astrocytic Glutamate Clearance Disruption

Normally, the astrocytic clearance of excess synaptic glutamate following synaptic transmission prevents the activation of extrasynaptic NMDARs coupled to proapoptotic pathways. Once taken up by astrocytes (via transporters EAAT1 and EAAT2), glutamate is converted to glutamine by glutamine synthetase (GS) and released into the interstitial space. Glutamine is then imported by both excitatory and inhibitory neurons and converted to glutamate by phosphate-activated glutaminase (PAG). In inhibitory neurons, glutamate is then converted to GABA by glutamate decarboxylase (GAD). Thus, astrocytes regulate the turnover of both glutamate and GABA, and a disruption of the glutamate–glutamine cycle may cause epileptogenesis [4,102]. Eid et al. showed that in brain samples surgically resected from MTLE patients (all with HS), GS immunoreactivity is decreased; the loss of GS is particularly pronounced in areas of the hippocampus with astroglial proliferation [76]. In a study by van der Hel et al., samples of TLE patients with or without HS were grouped separately. Samples with HS had reduced GS immunoreactivity, which was the most pronounced in regions with the most severe neuronal loss. However, the GS immunoreactivity in non-HS TLE samples was similar to that in control samples. Thus, GS downregulation is not simply a consequence of seizures, and it may contribute to the pathogenesis of HS [103].

In multiple studies with rodents, manipulations with RNA or protein levels of EAAT2 (or its murine analog GLT-1) showed the causal role of this glutamate transporter deficiency in seizures [4]. However, expression levels of EAAT1 and EAAT2 in human epileptic tissue are not necessarily decreased [76,104,105].

#### 2.4.4. Astrocytic Potassium Clearance and Water Transport Disruption

Due to the limited volume of the CNS extracellular space, even modest neuronal K^+^ efflux causes considerable changes in the extracellular K^+^ concentration ([K^+^]_o_). During an action potential (AP), [K^+^]_o_ increases several times and then gets quickly restored by Na^+^/K^+^-ATPases in neurons and glia, astrocytic Na–K–Cl co-transporters and astrocytic inwardly rectifying potassium channels (K_ir_). The conductance of K_ir_ channels increases when [K^+^]_o_ is raised. After uptake by an astrocyte, K^+^ is spatially buffered by redistribution within the syncytial network of gap junction-coupled astrocytes [106]. The downregulation or dysfunction of astrocytic K_ir_ channels (K_ir_4.1 in particular), as well as alterations in gap junction coupling, have been found in some types of epilepsy and some epilepsy models, and may be causative of epileptic activity [107,108,109]. Mice with a conditional knock-out of K_ir_4.1 directed to glia by the human GFAP promoter display a pronounced behavioral phenotype that includes seizures. However, this strain has K_ir_4.1 loss not only in astrocytes, but also in oligodendrocytes [110].

A downregulation of astrocytic K_ir_ channels has been observed in animal models of various neuropathologies, including posttraumatic epilepsy [59]. A correlation between decreased K_ir_4.1 expression in epilepsy-associated lesions and the local inflammatory environment, in particular an increased level of IL1β, was demonstrated in cell cultures, in the temporal cortex of rats with induced SE and in human astrocytic tumor specimens [111]. A link between albumin entering the brain after BBB damage, astrocytic K_ir_4.1 downregulation and neuronal hyperexcitability has been established in experiments with in vivo brain exposure to albumin in rats. It was also shown that albumin uptake by astrocytes is mediated by transforming growth factor β receptor (TGFβR) [109,112]. In a following study, it was shown that albumin/TGFβR signaling in astrocytes after BBB damage also leads to excitatory synaptogenesis [113].

Astrocyte gap junction coupling is inhibited by proinflammatory cytokines and lipopolysaccharide. In brain specimens from MTLE patients with HS, a complete lack of astrocyte coupling was found. In a mouse model of chronic MTLE with HS, it was shown that the impairment of astrocyte coupling starts very early, within 4 h after SE, and is probably mediated by the hyperactivity-induced release of proinflammatory cytokines. Astrocyte uncoupling not only impairs K^+^ homeostasis, but may also enhance extracellular glutamate levels [114]. However, there are also reports about increased astrocyte coupling associated with epilepsy. Dye coupling between astrocytes (dissipation of an intracellularly injected dye into adjacent cells) and the expression level of connexin 43 (a major gap junction protein in astrocytes) were increased in the hippocampus of KA-treated rats [115]. In addition, a surprisingly large capacity for K^+^ clearance was conserved in hippocampal slices of mice with coupling-deficient astrocytes (Cx30 and Cx43 double knock-out), indicating that gap junction-dependent processes only partially account for K^+^ buffering in the hippocampus [116].

Another process affecting extracellular space volume and extracellular ion concentrations is water transport through cell membranes. AQP4 is an aquaporin highly expressed by astrocytes. AQP4 and K_ir_4.1 colocalize in astrocyte end feet; it is suggested that AQP4 functions in concert with K_ir_4.1. Changes in AQP4 expression have been observed in animal and human epilepsy, and in experiments with a functional loss of either K_ir_4.1 or AQP4, seizure susceptibility was enhanced [4]. Even a mislocalization of AQP4 when its astrocytic expression is not lost causes a disruption of K^+^ clearance. Mice with knocked-out α-syntrophin show a loss of AQP4 only in perivascular membranes of astrocyte end-feet. In these animals, a delayed K^+^ clearance in hippocampal slices and an increased severity of induced seizures were demonstrated [117]. In KA-treated rats, it was shown that a redistribution of AQP4 from perivascular membranes to other astrocytic plasma membrane domains precedes the onset of chronic seizures [118].

Some proposed glia-targeting epilepsy treatments that aim to restore the disrupted excitation–inhibition balance alleviated seizures in rodent models. For example, to normalize glutamate clearance, Gao et al. used saikosaponin to increase astrocyte glutamate-uptaking capacity [119], while Sha et al. utilized a pharmacological inhibition of Hsp90 to prevent the proteolysis of glutamate transporter GLT-1 in astrocytes [120]. Zhao et al. expressed channelrhodopsin-2 in astrocytes and showed that their optogenetic activation inhibits high-firing pyramidal neurons during cortical seizure by a mechanism involving actrocytic Na^+^/K^+^-ATPase, which may potentially be used for gene therapy [121].

#### 2.4.5. Aberrations in Gliotransmission

One of the normal functions of astrocytes is glutamate release, through which astrocytes control the excitability, plasticity and synchronous activity of synaptic networks. A recent work using single-cell transcriptomics confirmed that glutamatergic astrocytes are a specialized astrocyte subtype; their contribution to seizures was also tested. Acute seizures were triggered by kainate in mice with astrocyte-specific *Vglut1* knock-out and control animals. Mutant mice underwent more seizure episodes, with individual episodes being longer. These data, unexpectedly, reveal an anti-seizure role of astrocyte VGlut1-dependent glutamate signaling [122]. More than 50% of glioma patients report seizures. It was shown that increased glutamate release from the tumor is caused by the overexpression of the catalytic subunit of the cystine/glutamate transporter System x_c_^−^ (encoded by the *SLC7A11* gene). An increased level of extracellular glutamate induces excitotoxicity in the surrounding neurons [123].

Héja et al. demonstrated a link between glutamate and GABA transport in astrocytes. It was found that glutamate uptake results in GABA release from astrocytes through the reversal of GABA transporters. This coupling between glutamate and GABA transport was abolished in the presence of non-transportable blockers of glial glutamate (EAAT1/2) or GABA (GAT3) transporters [124]. The same group then studied glutamate uptake-induced glial GABA release in rat hippocampal slices. It was found that, during sustained excitation, GABA released from astrocytes in response to glutamate transporter activation significantly contributes to the tonic inhibition of CA1 pyramidal cells. Since this glial glutamate/GABA exchange was observed only during enhanced network activity, it may be considered an adaptive neuroprotective mechanism. In an in vitro model of epilepsy (low-Mg^2+^-induced epileptiform activity in hippocampal slices), the blockade of the glutamate/GABA exchange increased the duration of seizure-like events [125]. Pandit et al. also demonstrated the neuroprotective role of astrocytic GABA release in an epileptic brain. They found that the GABA-permeable Bestrophin-1 (Best1) channel is responsible for tonic GABA release from astrocytes necessary to maintain the excitation/inhibition balance; GABA_A_R tonic current was increased in CA1 pyramidal neurons in hippocampal slices obtained from mice with epilepsy induced by KA or pilocarpine. A pharmacological inhibition of Best1 increased seizure susceptibility in these models. In mice with Best1 knockout, an ectopic expression of Best1 only in astrocytes was sufficient to rescue elevated seizure susceptibility in the KA model [126].

Another important gliotransmitter is adenosine, an endogenous anticonvulsant and seizure terminator. Adenosine level in the extracellular space is mostly controlled by astrocytic adenosine kinase (ADK). Adenosine homeostasis is disrupted in animal models of epilepsy and in human epilepsies. ADK undergoes biphasic expression change during epileptogenesis; acute brain insults lead to a surge in adenosine associated with a transient downregulation of ADK within hours after the injury; then, in the latent period of epileptogenesis, developing astrogliosis is associated with increases in ADK expression and adenosine deficiency. It was shown that seizures originate in areas of astrogliosis with an overexpression of ADK, and that an artificial overexpression of ADK in transgenic animals is sufficient to generate seizures. In addition, increased ADK expression drives increased DNA methylation, associated with epileptogenesis [127].

D-serine may be another gliotransmitter (it is not clear whether it is synthesized in astrocytes or in neurons). It is a co-agonist of conventional NMDA receptors and an antagonist of *t*-NMDARs (triheteromeric NMDA receptors). D-serine levels are depleted in the brains of epileptic rats in the pilocarpine model. A focal administration of D-serine to the medial entorhinal area (MEA) attenuated neuronal loss in this region and prevented epileptogenesis. Treatment with D-serine reduced astrocyte counts in the MEA, altered their reactive status, and attenuated the proliferation and/or infiltration of microglia to the region, alleviating neuroinflammation [128].

A correction of adenosine transmission by astrocytes to prevent seizures may be achieved with gene therapy; it was effective in some experiments with rodents. To alleviate ADK overexpression in astrocytes and normalize adenosine signaling, Young et al. used a vector expressing a microRNA targeting ADK transcript [129], while in the study by Theofilas et al., an antisense RNA was expressed for the same purpose [130].

#### 2.4.6. Extracellular Matrix Remodeling by Reactive Astrocytes

Major components of neural ECM, including chondroitin sulfate proteoglycans, tenascin-R and hyaluronic acid, are synthesized by both neurons and glia. The expression of these molecules is dynamically regulated during brain development [131]. The dynamic nature of the ECM depends on the constant remodeling by proteases, including matrix metalloproteinases (MMPs). In the mature brain, ECM stabilizes synapses, regulates synaptic plasticity and prevents abnormal synaptic remodeling. Perineuronal nets (PNNs) are ECM structures surrounding synapses of select neuronal subpopulations, mostly GABAergic interneurons [132]. In pathological conditions, reactive astrocytes overproduce some ECM molecules, which causes a growth-impermissive environment and impaired synaptic plasticity. These molecules are mainly responsible for limiting axonal elongation on the glial scar [131]. On the other hand, the degradation of ECM molecules leading to the destabilization of PNNs could cause uncontrolled sprouting and synaptogenesis, and aberrant synaptogenesis is characteristic for epilepsy [132]. In animal models of epilepsy and in human epileptic tissues, a pathological remodeling of the ECM and PNNs, as well as upregulated levels of MMPs, have been reported [4,133]. Moreover, in different animal models, a deficiency of ECM components caused altered neuronal activity and/or seizures [4]. For example, an enzymatic digestion of hyaluronic acid by application of hyaluronidase was sufficient for inducing seizure-like activity in vitro [134] and epileptogenesis in vivo [135]. Seizures cause an imbalance of MMPs and their inhibitors, which leads to the proteolysis of neurotrophins, proteoglycans, and integrins, associated with structural and functional changes in the cortex and the mesocorticolimbic system, which further increase seizure susceptibility [136]. One of the mechanisms linking glioma to seizures is matrix-degrading enzymes (MMPs and others) released by the tumor. They degrade PNNs around parvalbumin-positive (PV+) interneurons, resulting in increased membrane capacitance, reduced firing, decreased GABA release and eventually causing the death of these neurons. While there is an overall neuronal loss in the peritumoral cortex, PV+ interneurons are more affected compared to other neuron types [137]. In cases of seizures associated with abnormalities in PNNs causing the selective loss of interneurons, transdifferentiation therapy may prove useful; it is convenient that many existing transdifferentiation methods generate predominantly GABAergic interneurons (see Section 4.1, Section 4.7 and Section 6.2).

Another mechanism by which the ECM disruption may contribute to epileptogenesis is an impaired ion buffering function. Normally, chondroitin sulfate proteoglycans have a very high density of negative charges in their side chains. These stationary charges act as a buffering system for extracellular cations and repel anions. Damage to the ECM could change neuronal excitability by changing extracellular and intracellular ion concentrations [4].

It was shown that in kindled rats, PNN breakdown and seizure activity can be inhibited by treatment with doxycycline, a broad-spectrum inhibitor of MMPs [138].

#### 2.4.7. Alterations in Glucose and Lipid Metabolism in Astrocytes

Neurons have high oxidative activity, while astrocytes show a predominance of glycolysis and can store glycogen. Neurons use up to 90% of the total energy consumed by the brain; however, during brain activation, the increase in glucose uptake occurs mostly in astrocytes. Glycolysis and glycogenolysis in astrocytes produce lactate, which is transferred to neurons. Neurons use lactate (after its conversion to pyruvate) as their preferred mitochondrial energy substrate. It is supposed that the astrocyte–neuron lactate shuttle only operates at excitatory synapses; GABAergic neurons likely rely on their own glycolytic activity [139]. Considering glia-to-neuron transdifferentiation, a switch to oxidative phosphorylation and accompanying changes in the gene expression and morphology of mitochondria are among the most important processes happening in transitioning cells; a large fraction of cells die from oxidative stress before their mitochondria become neuron-like and less vulnerable to it (see Section 4.1).

In epilepsy, astrocytes are crucial to fueling epileptiform activity. In experiments where this activity was acutely provoked, glucose trafficking through the coupled astrocytic network was increased to match the enhanced energy need of the hyperactive neuronal circuitry. Increased levels of glycogen have been found in the hippocampus of TLE patients. Seizures are associated with an increase in extracellular glutamate, and glutamate uptake by astrocytes stimulates astrocytic glycolysis. Thus, excessive synaptic activity causes a rapid drop of glucose and a corresponding rise in lactate [140]. Some key proteins participating in the astrocyte–neuron lactate transfer are upregulated in epilepsy. One example is lactate dehydrogenase (LDH), which converts lactate to pyruvate. Sada et al. showed that seizures and epileptiform activity are reduced by LDH inhibitors [141]. Later, the same research group found that the LDH subunit LDHA is overexpressed in the hippocampus of mice with TLE induced by kainate [142]. Monocarboxylate transporter 1 (MCT1) facilitates the transmembrane transport of multiple molecules including lactate and pyruvate. Lauritzen et al. found that MCT1 is overexpressed on astrocytes in hippocampal formation in three different rat models of TLE. This is in agreement with some previous reports of increased extracellular lactate concentration in the hippocampus of patients with TLE [143].

Importantly, lactate in the brain also has a signaling function. Lactate transfer from astrocytes to neurons was found to be necessary for memory consolidation. The excitability of several populations of neurons is modulated by lactate, with most results indicating that lactate increases excitability [139]. One of the proposed mechanisms is lactate acting as a modulator of already activated NMDAR receptors, potentiating NMDAR-dependent inward current and Ca^2+^ influx evoked by the application of glutamate and glycine [144]. This role of lactate may, in theory, contribute to the excessive synaptic activity associated with epileptogenesis. However, there are experimental confirmations that increased extracellular lactate is, in fact, neuroprotective against excitotoxicity [139] and may even terminate seizure activity and contribute to postictal refractoriness in epilepsy via the acidification of the extracellular compartment [143,145]. More recent studies revealed a more specific mechanism of the anti-epileptogenic action of lactate. It was shown that lactate negatively modulates neuronal excitability by interacting with hydroxycarboxylic acid receptor 1 (HCAR1). This receptor is abundantly expressed in mossy cells, which are key components of the hippocampal excitatory circuit [146].

An increase in lipid droplet formation was observed in neurons and astrocytes in epileptic foci of patients with TLE and mice with KA-induced epilepsy. However, transcriptomic analysis showed that in TLE, pathways related to lipid transport and metabolism are activated in astrocytes much more prominently than in neurons. *APOE* (apolipoprotein E) was one of the most differentially expressed genes between TLE participants and controls in astrocytes. A distinct astrocyte subpopulation, named lipid-accumulated reactive astrocytes (LARAs), showed a particular enrichment of lipid transport-related pathways, and the highest level of *APOE* expression. Experiments in vitro have demonstrated that hyperactive neurons release fatty acids, which are taken up by astrocytes via an APOE-dependent mechanism. Experiments with KA-induced epilepsy in Apoe^−/−^ mice showed that APOE is required for lipid accumulation in astrocytes. Constitutive or astrocyte-specific *Apoe* knockout reduced seizure activity and rescued neuronal loss in the hippocampus of KA-treated mice [49].

There are some therapeutic approaches proposed to prevent neuronal hyperexcitability by normalizing energy metabolism in astrocytes. Drugs supporting mitochondrial metabolism, like acetyl-L-carnitine and triheptanoin, may be used; antiseizure mechanisms involving these substances partially overlap with the effects of ketogenic diet (reviewed in [147]).

### 2.5. Radial Glia-like Neural Stem Cells, Adult Neurogenesis and Epilepsy

Radial glia (RG) is a cell type that, in mammals, is found only in the developing nervous system. These cells have a characteristic morphology, with apical–basal polarity, and very long and thin filaments. RG originate from the early neural tube neuroepithelial cells, and eventually differentiate into neurons or glia [148,149,150]. In addition to their progenitor function, RG cells provide their filaments as a scaffold necessary for neuroblast migration in embryonic development [148,151]. RG differ from neuroepithelial cells (morphologically very similar) by showing some hallmarks of astrocytes, such as glycogen granules and the expression of some astrocyte-associated proteins [152]. Progenitors similar to RG (radial glia-like cells, or RGLCs), which function as neural stem cells (NSCs), are found in some regions of the adult mammalian CNS. NSCs resemble astrocytes by their phenotype, including not only the expression of certain proteins, but also ultrastructural characteristics like bundles of intermediate filaments, multiple processes intercalating between other cells, and gap junction complexes [149]. Moreover, NSCs may perform typical astrocytic functions of supporting neuronal and synaptic activity [148,150,153,154]. In addition, it was shown that when NSCs stop producing new neurons, they may terminally differentiate into astrocytes [155]. Such close similarity between NSCs and astrocytes may lead to confusion; indeed, in one of the early studies of adult neurogenesis, they are considered the same cell type [156]. However, more recent research, including transcriptomic studies, accumulated evidence that adult NSCs should not be classified as astrocytes, even though this view is still not universally accepted [157]. The confusion is made worse by the fact that the nomenclature of these cells is not clear; in different works, they may be called RGLCs, NSCs, radial astrocytes [148], astrocyte-like stem cells [158] and sometimes even simply “astrocytes” or “a type of astrocytes” [149,150,154,156,159,160,161,162,163]. In addition, there are specific terms for RGLCs or cells with similar properties depending on the region where they are found; for example, B1 cells in the ventricular-subventricular zone (V-SVZ), type-1 progenitors in the subgranular zone (SGZ) [161], radial ependymoglial cells in the spinal cord, Bergmann radial glia in the cerebellum and Müller glial cells in the retina [148]. Despite their shared properties, NSCs in different CNS regions differ in their phenotype and surroundings [161,164]. The identification of RGLCs is complicated by the fact that many immunoreactive markers of these cells are also expressed by other CNS cell types, especially astrocytes. The “classic” markers used to identify RG and RGLCs are EAAT1 (SCL1A3), glutamate synthase, S100β, tenascin C, brain lipid binding protein, SCO-spondin, GFAP, nestin, and vimentin [148,150,165]. However, different RGLC subtypes express these markers in different combinations [148]. For example, GFAP-positive type-1 progenitors are S100β-negative and thus may be distinguished from SGZ astrocytes [166,167]. Moreover, for both embryonic RG [148,150,163,168] and adult NSCs [150,169], it was shown that even within the same region these cells comprise heterogeneous populations, with subpopulations expressing different gene sets.

NSCs in mammals have been best described in rodents, in the “canonical” adult neurogenesis niches, SGZ and SVZ (also called subependymal zone, or SEZ [170]), even though adult neurogenesis in other brain regions was also observed in different mammalian species [171,172,173]. Importantly, in the human brain, the striatum is suggested to sustain neurogenesis [174]. NSCs in the canonical niches may receive proliferation-regulating signals from blood, CSF and neighboring cells; neurotransmitters released from projections from remote brain regions also affect NSC activity [161]. Normally, SVZ produces neuroblasts that migrate into the olfactory bulb, and SGZ gives rise to neuroblasts that are directed into the hippocampus. These neurogenic niches may also participate in compensatory neurogenesis in neuropathologies. In these cases, the neuronal precursors generated in these zones may migrate to the site of the injury, directed by the gradient of inflammatory mediators and/or growth factors [175]. A subset of NSCs in the SVZ are “dormant” and require triggering by interferon signaling associated with injury to enter the “primed quiescent” state followed by NSC activation [176]. In the damaged brain, the neurogenic zones also produce reactive astrocytes. Faiz et al. showed that in the stroke-injured mouse cortex, SVZ-derived cells differentiate into reactive astrocytes and contribute to astrocyte scar formation. It was also shown that these astrocytes may be converted into neurons by manipulations with *Ascl1* expression (described in detail in Section 4.3) [177].

Even though both SVZ and SGZ are known to respond to seizures by increased neurogenesis [178,179], SGZ is usually the zone of interest in epilepsy studies because it is located in the hippocampus, which is often involved in epileptogenesis. There is much evidence that seizures-induced aberrant neurogenesis in SGZ contributes to the anatomical reorganization of the hippocampus in TLE [178,180,181,182,183]. In rodent models of epilepsy, adult hippocampal neurogenesis is increased after acute and mild seizures, but decreased by chronic and severe epilepsy. A similar decrease is observed in patients with chronic epilepsy [182,183,184,185]. One possible explanation is the difference in long-term survival of newborn neurons depending on SE severity [186]. Pathways regulating neurogenesis in the healthy brain may be considered potential druggable targets for epilepsy, but for many of them direct evidence linking them to seizures is lacking (reviewed in [187]). One suggested mechanism connecting seizures to neurogenesis is activity-dependent DNA demethylation in mature neurons (regulated by GADD45B) causing an expression of paracrine neurogenic factors [188]. The sonic hedgehog (SHH) signaling pathway is also important for this process [189,190].

Seizures change NSC properties. Sasaki-Takahashi et al. compared these cells in the hippocampus of mice that experienced seizures with or without SE in an improved pilocarpine model. Gfap-Gfp transgenic mice were used to visualize GFAP-expressing cells, including NSCs. At 10 days after SE, the number of NSCs with an apical process decreased, the processes of NSCs extended inversely toward the hilus, and about a half of these cells started expressing S100β. These changes in NSCs may suggest a shift towards astrocyte differentiation. In this study only seizures without SE, but not seizures with SE, increased proliferation of NSCs [184]. New neurons born after seizures also show unusual properties. Various structural abnormalities, as well as aberrations in migration of these cells were reported [74,178,181,191,192]. Notably, heterotopic neurons are rare in other brain diseases, but are consistently reported in epilepsy [59]. Seizure-born neurons fail to integrate into hippocampal circuits important for memory formation [193]. Neurons born after seizures increase overall network excitability by different mechanisms, contributing to epileptogenesis [192,194,195,196]. However, there is also evidence that these neurons may promote a reduced overall excitability of the DG (importantly, in this study, seizure-born neurons were compared with those generated after voluntary running, a physiological trigger of neurogenesis in a healthy brain) [197].

Different methods of suppressing adult neurogenesis were used in studies linking neurogenesis to epilepsy. Irradiation had no effect on seizures, or exacerbated them [198,199], while administration of drugs suppressing cell proliferation alleviated seizures [96,200]. However, both these strategies have off-target effects that may seriously affect the results [187]. Cho et al. used a cell type-specific genetic approach: Nestin-TK transgenic mice, in which the administration of ganciclovir eliminates only dividing cells with an active nestin promotor. Ablation of neurogenesis by this method achieved a long-term reduction in spontaneous recurrent seizure frequency in pilocarpine model and rescued cognitive deficits in epileptic animals [201]. Leibowitz et al. used another subtle approach to reduce hippocampal neurogenesis: they ectopically expressed somatostatin in the hippocampus of electrically kindled rats using an adeno-associated virus (AAV) vector. In 5 out of 13 animals, this treatment blocked evoked seizures. Histological analysis showed that in the hippocampi of the animals who responded to the treatment (but not in the non-responders), the kindling-stimulated increase in type-1 progenitor proliferation was reversed [202].

Since NSCs naturally produce neurons, it seems tempting to use them for cell therapy of various neuropathologies, and simply transplanting them to a damaged brain region without additional manipulations may be sufficient. Another function of NSCs that makes them valuable for cell therapy is the secretion of multiple neuroprotective factors [203]. However, there are obstacles to using these cells in treatments. One problem is that obtaining autologous NSCs of the patient for manipulations in vitro would be complicated, so treatments with factors that promote NSC proliferation or the migration of their progeny to the damaged area in situ are much more preferable. An alternative is to generate NSCs from ESCs or iPCSs, but there is a known risk of tumorigenesis [204,205]. Another obstacle is the poor survival of grafted NSCs in a pro-inflammatory microenvironment. Nevertheless, there are some studies wherein NSCs have been successfully used to generate new neurons in disease models. Sakata et al. proposed a way to overcome the accelerated death of grafted NSCs by using minocycline, an antibiotic with cytoprotective properties. In a rat stroke model (transient middle cerebral artery occlusion, tMCAO), transplantation of the minocycline-preconditioned donor fetal NSCs attenuated infarct size and improved neurological performance [206]. Tang et al. worked with a different stroke model and used *Dlx2* overexpression to differentiate NSCs into GABAergic neurons and reduce the neurological damage [207]; this paper is described in detail in Section 4.7. Experiments with NSC transplantation in epilepsy models are described in Section 5.2.

### 2.6. Oligodendrocyte Precursor Cells and Their Pathological Changes in Epilepsy

Oligodendrocyte precursors express nerve/glial-antigen 2 (NG2) and are also called “NG2-positive glia”. These cells are also known as polydendrocytes or O-2A progenitor cells. OPCs are the fourth major glial cell type identified in the 1990s, much later than the classically recognized astrocytes, oligodendrocytes and microglia. In the CNS, OPCs comprise 2-8% of all cells, and are the main proliferating cell type [208,209,210,211]. OPCs are found throughout the gray and white matter, in both the developing and the mature CNS; in the mature brain, they are uniformly distributed [208,210,211]. A study using in vivo two-photon imaging demonstrated that OPCs in the murine cortex are highly dynamic and continuously migrate [212].

NG2 (chondroitin sulfate proteoglycan 4, also called CSPG4 [213,214]), is a molecule characteristic for these cells, but it is not expressed exclusively by them. Some pericytes [208,215] and activated macrophages or microglia observed in brain lesions [216,217] also express NG2; however, they are morphologically different from OPCs. Other markers expressed by OPCs are alpha receptor for platelet-derived growth factor (PDGFRα), the immature neural ganglioside recognized by the monoclonal antibody A2B5 [218,219], and oligodendrocyte transcription factor 2 (Olig2) [220,221]. OPCs are self-renewing resident progenitors that retain their proliferative ability throughout their whole life. The primary function of these cells is producing oligodendrocytes. However, they also produce astrocytes (mostly prenatally) [208,222]. Similar to stem cells, OPCs can divide asymmetrically, with one daughter cell keeping a progenitor phenotype and the other proceeding to differentiation [210]. There are controversial data regarding the ability of OPCs to produce neurons. This is discussed in detail below in Section 3.2.

In the intact adult brain, only a small fraction of OPCs are actively proliferating [210,211,223]. This suggests that these cells have other functions beside oligodendrogenesis. OPCs express multiple ion channels and neurotransmitter receptors and establish physical contacts with neurons (including synapses), other types of glial cells, endothelial cells and pericytes [210,224]. Moreover, OPCs receive glutamatergic and GABAergic synaptic contacts, which are remarkably similar to neuron-to-neuron synapses [210,224,225,226]. Alterations in neurotransmission affect the proliferation and differentiation of OPCs. Experiments with the selective ablation of OPCs showed that these cells provide trophic or structural support to dendrites and participate in glutamatergic neurotransmission (reviewed in [210]). Auguste et al. showed that OPCs participate in neural circuit refinement by the engulfment of presynaptic inputs, and are likely able to internalize synapses through phagocytosis [227]. Káradóttir et al., working with acute cerebellar slices from rat pups, discovered two distinct electrophysiological types of OPCs. Spiking and non-spiking OPCs have the same morphology and express similar markers, but only spiking OPCs express voltage-gated Na^+^ and K^+^ channels, receive synaptic input from neurons and, surprisingly, generate APs [221]. However, according to other authors, the depolarization-induced spikes generated by OPCs do not fit the criteria of proper APs [228,229]. Different scientists also had different opinions about the existence of electrophysiologically distinct OPC types [228,229,230]. It was later established that OPCs start out as a homogeneous population but become heterogeneous later, with ion channel expression, electrophysiological properties and differentiation potential varying between OPCs in different brain regions and at different ages [231].

OPCs cells respond to acute CNS insults (which may or may not involve demyelination) by migration towards the lesion site, increased proliferation, increased NG2 expression and dramatic morphological changes (swollen cell bodies and changes in the shape of processes) [208,210,211,213,232,233]. OPC response may be detected as early as 24 h after the lesion, similar to the microglia activation and earlier than the astrocyte activation [208,233].

There are some data indirectly implicating that OPCs may be involved in the pathogenesis of epilepsy. Among the injuries triggering OPC activation in animal models is KA-induced excitotoxicity [216,234]. Káradóttir et al. showed that spiking OPCs generate larger membrane currents in response to glutamate or NMDA compared to non-spiking OPCs. In slices exposed to ischemia (a condition involving increased extracellular glutamate level), increased cell death was observed only for the spiking OPC type; this could be prevented by glutamate receptor antagonists. Thus, spiking OPCs may be more vulnerable to glutamate-mediated damage [221].Studies where the changes in OPCs were addressed specifically in the epileptic brain are few. Wu et al. demonstrated that in pilocarpine-induced epilepsy in mice, the number of OPCs in CA1, CA3 and DG hippocampal regions is increased at 1 day–2 months after the SE. The differentiation of these cells into oligodendrocytes was not estimated, but immunostaining showed that these extra OPCs likely do not differentiate into neurons or astrocytes [235]. Similar results were reported by Wennström et al. for rats subjected to an electroconvulsive seizure: the number of OPCs in the DG was increased at 2 days and at 3 weeks after the seizure. None of the proliferating cells in this study expressed the oligodendrocyte marker CNPase [236]. Luo et al. registered a loss of myelin and oligodendrocytes in the hippocampus of rats with lithium–pilocarpine-induced epilepsy, as was shown by the decreased expression of myelin basic protein (MBP) and decreased number of cells positive for APC (adenomatosis polyposis coli, detected by CC1 antibody). The severity of myelin loss increased during epileptogenesis. At the same time, the number of OPCs increased; staining for Ki67 revealed their proliferation. An enlargement of OPC soma and processes and an increased NG2 expression were also observed. Both the number of OPCs and the fraction of proliferating OPCs peaked during the latent phase of epileptogenesis and decreased during the chronic phase. During the late chronic phase, epileptic animals had fewer OPCs and smaller percentages of Ki67+ OPCs in the hippocampus than the control group. Protein expression levels of Olig1 and Olig2, TFs crucial for the proliferation and differentiation of OPCs into oligodendrocytes, were upregulated during the acute phase, peaked during the latent phase, and sharply declined during the chronic phase of epileptogenesis [69]. Heinrich induced SE in NG2creER:YFP mice using pilocarpine, and performed an immunohistochemistry analysis of hilus samples. An increased number of OPCs at 2 days after SE was registered. However, there was no increase in the number of GFP+ cells (GFP in this strain marks all cells that had once expressed NG2). Staining for GFP and MBP showed that after the seizure activity, fewer OPC cells differentiate into oligodendrocytes. Thus, increased NG2 immunoreactivity is not a result of increased cell proliferation, but is caused by the accumulation of OPCs not proceeding to differentiation. Importantly, the author notes that NG2creER:YFP mice showed resistance to seizure induction and may not be a good model in epilepsy studies [237]. Hu et al. studied resected brain tissue from patients with intractable epilepsy (different brain regions). They observed decreased levels of MBP and other myelin-associated proteins in epileptic foci. Immunohistochemistry showed that the numbers of OPCs (A2B5+, PDGFRα+ cells) and pre-oligodendrocytes (O4+, PDGFRα+) were increased, while the numbers of immature (O4+, MBP−) and mature oligodendrocytes (MBP+) were decreased [238]. However, You et al. did not register any change in OPC number in the hippocampus or cortex of rats with PTZ-induced epilepsy. They showed that myelin sheath damage in this model is mediated by autoantibodies against MBP [239]. Taken together, these reports suggest that epileptogenesis may provoke OPC proliferation, impairing their differentiation into mature oligodendrocytes at the same time, which may be one of the reasons for the demyelination sometimes observed in epilepsy. Demyelination, in turn, may contribute to hyperexcitability and seizure generation [68].

**Figure 2:**
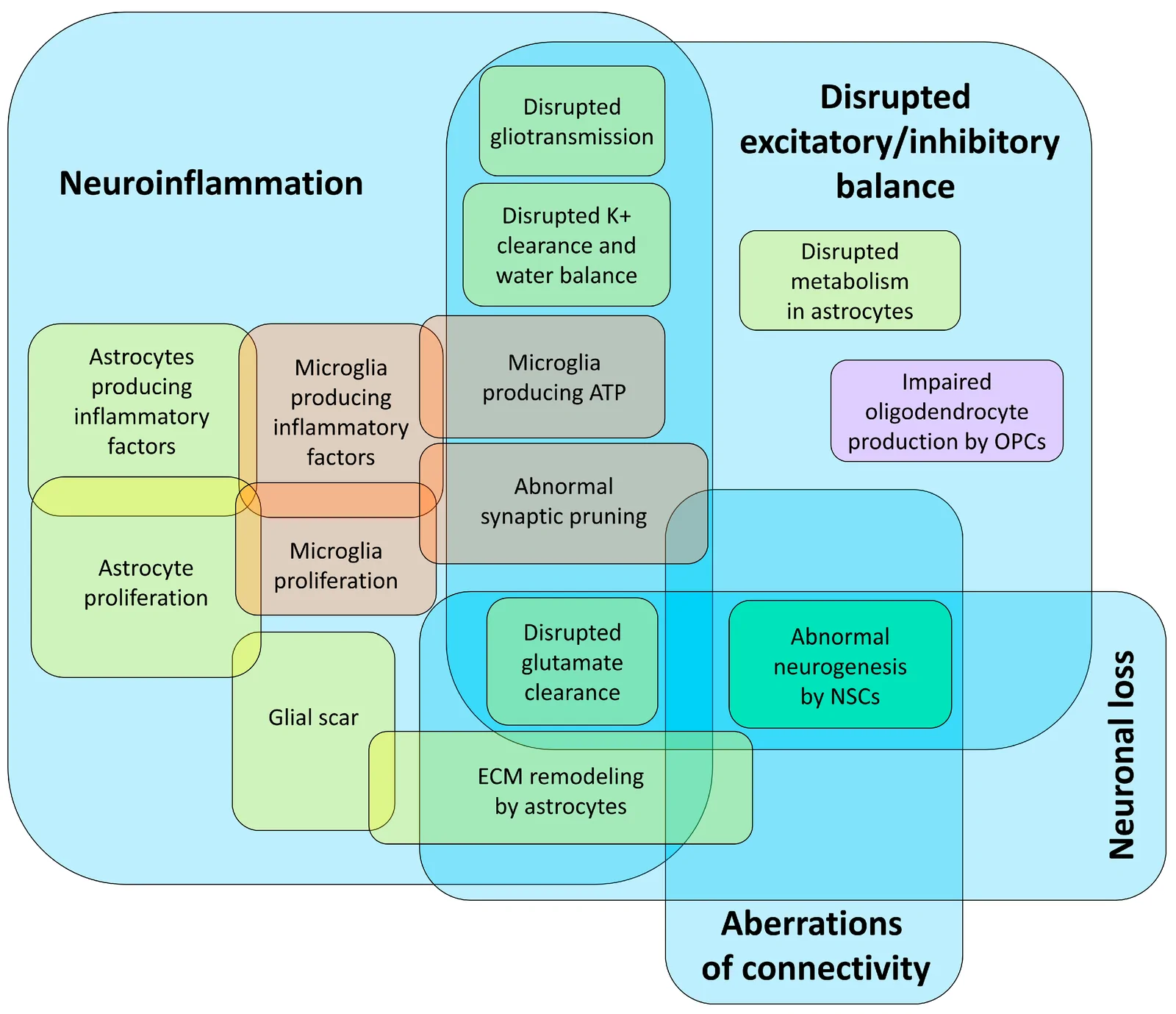
A schematic representation of pathological changes in glial cell functions observed in the epileptic brain and associated changes in neurons, as described in the papers cited in Section 2. Color denotes the cell type involved in the mechanism: astrocyte (yellow), microglia (red), NSC (green), OPC (purple), neuron (blue). Intersections between bubbles mean that these mechanisms are frequently observed together; in many cases, it is hard to establish a causation, since a disruption of a glial mechanism may be both a consequence of seizures and a part of epileptogenesis.

The changes in glial cells observed in epilepsy are summarized in Figure 2.

## 3. Neurogenic Potential of Glial Cells; Arguments for Using Glia to Generate Neurons and Methods of Tracing the Origin of New Neurons

In this section, we will describe similarities between glial cells and NSCs and their natural ability to generate neurons in the developing or injured brain (or in experiments in vitro aimed at reproducing neurogenesis in these conditions, including neurosphere culture). This does not include transdifferentiation experiments where glial cells were exposed to some specific factor known to induce neurogenesis; such experiments are described separately in Section 4.

It should be noted that even the earliest progenitor cells in the developing CNS, namely, neuroepithelial cells and RG, in vivo and in primary cultures normally generate only a single type of progeny, with a small fraction generating two different cell types. Multipotency in these embryonic cell types and in NSCs isolated from adult neurogenic niches was observed only in vitro upon growth factor exposure, such as in neurosphere culture conditions. No cells are known to be capable of producing neurons, astrocytes and oligodendrocytes at the same time in vivo. Thus, the definition of a “neural stem cell” is rather vague [153]. Moreover, the ability to produce neurospheres in vitro was also shown for some secondary progenitors that are not considered stem cells, like OPCs or type C cells in SVZ [149].

While the potential ability of different cell types to produce neurons is more easily demonstrated in vitro, in vivo the microenvironment in the brain parenchyma outside of the neurogenic niches inhibits neurogenesis and favors gliogenesis, as was shown in experiments with the transplantation of NSCs into different CNS regions [240,241]. However, a number of studies confirmed that new neurons can be generated locally in the brain parenchyma, at least in some areas and conditions. Earlier works excluded the contribution from the NSCs in the adult neurogenic niches but did not clarify the cellular source of the newborn neurons. Subsequent studies investigated astrocytes and OPCs as the most likely suspects of local neurogenesis (reviewed in [242]).

Cells with neurosphere-forming capacity in the brain parenchyma outside of the neurogenic niches are rare: only 2–5 out of 10,000 cells dissociated from the adult murine cortex can form neurospheres at all. In most cases, such neurospheres are not multipotent and have limited self-renewal capacity [153]. Multiple studies, including the ones described below, have demonstrated that brain lesions induce compensatory neurogenesis by different mechanisms. However, these mechanisms are ineffective, and mammalian brain regeneration capacity is very limited; an important factor here is that most newly generated neuroblasts do not survive. For example, it was estimated that 6 weeks after stroke in rats, the fraction of dead striatal neurons that has been replaced (by neurons generated in both SVZ and striatum parenchyma) is only about 0.2% [243]. Thus, the ongoing research in the neuroregeneration field is focused on the ways to boost the inherent brain regeneration mechanisms by artificial stimulation (including factors described in Section 4).

One of the key methodic concepts in studies of cell differentiation or transdifferentiation is lineage tracing (the terms “cell fate tracing” and “fate mapping” are also used). It includes different approaches to labeling individual cells while they belong to the starting cell type, and keep them labeled after their transformation and possible change of morphology and/or proliferation. One way to do this is to use transgenic mouse strains that only express the marker in a specific cell type (genetic tracing). With wild-type (WT) animals, viral tracing may be used instead; for example, to trace the fate of astrocytes that are supposed to become neurons, a virus expressing the reporter under control of an astrocyte-specific promoter may be administered together with the neurogenic treatment. The labeling virus may infect different cell types, but should express the reporter only in astrocytes. However, during the conversion, astrocyte-specific gene expression is downregulated, and so the expression of the reporter from the astrocyte-specific promoter in the virus also decreases. To overcome this, a two-vector Cre-mediated flip-excision (Cre-FLEX) system may be used, where one virus expresses Cre recombinase from a cell-type specific promoter, while the expression of the reporter transgene from the other virus with a universal promoter must be “switched on” by Cre-dependent recombination once, and then remains stable. Transgenic mice and viruses with cell type-specific promoters may be combined: a reporter gene in the mouse genome may need switching on by Cre expressed from a virus [244,245,246]. Cre-mediated recombination may be induced at a specific time point. For this, CreER, a fusion between Cre and a mutated form of estrogen receptor activated by a metabolite of tamoxifen, is used. After tamoxifen is given to mice, CreER binds with its ligand and translocates from the cytoplasm to the nucleus, where it can perform genomic recombination [244,245,247]. Cell fate tracing using gene expression may be combined with other types of labeling, like bromodeoxyuridine (BrdU) incorporation by proliferating cells or the incorporation of PKH26 lipophilic dye by cells capable of phagocytosis [244,248]. In experiments with cell transplantation, donor cells may be taken from fluorescent reporter-expressing animals [240,249] or from another species, which allows their identification by species-specific proteins [219,250].

### 3.1. Neurogenic Potential of Astrocytes and Their Similarities to Neural Stem Cells

In cell reprogramming research, astrocytes may be considered the ideal starting cell type to generate new neurons in situ because of their ability to divide, their abundance in the brain (astrocytes constitute ~10–20% of CNS cells in mice [251]), and their proximity in lineage distance to neurons [252]. Moreover, there is evidence that astrocytes (or cells identified as astrocytes) are able to generate neurons in vitro and in vivo even without specific manipulations. In some conditions, astrocytes may express a neurogenic program in which they undergo phases typical of NSCs of the adult neurogenic niches: after activation they generate ASCL+ transit-amplifying progenitors (TAPs), which in turn give rise to DCX+ neuroblasts (reviewed in [242]).

#### 3.1.1. Neurogenesis by Astrocytes in the Healthy Brain

There are many similarities between astroglia (resting or activated), proliferating RG in the developing brain, and adult NSCs, as had been described in Section 2.5. In fact, the degree of similarity between astrocytes and NSCs is so high that it provides the technical challenge of accurately identifying astrocytes in experiments aimed to manipulate astrocytes with the purpose of facilitating CNS regeneration [253]. Single-cell transcriptomic analyses showed that astrocytes and NSCs at different stages of activation belong to the same continuum, and that quiescent (not proliferating) NSCs are very similar to astrocytes in their gene expression profile [254,255]. Multiple key pathways involved in NSC quiescence (including Notch and GABA signaling pathways) are strongly active in parenchymal astrocytes, and some of these pathways also regulate astrocyte morphogenesis (reviewed in [154]).

In mice, during embryonic and early postnatal development, astrocytes from different CNS regions possess some stem cell properties: in vitro, up to 10% of these astrocytes can form neurospheres that give rise to both neurons and glia. For the regions that are not adult neurogenic niches, astrocytes’ ability to form neurospheres disappears near the end of the second postnatal week [256].

Even fully differentiated astrocytes possess remarkable transcriptomic and physiological plasticity. They are equipped with multiple receptors and intracellular signaling cascades to respond quickly to changes in their environment [34]. Astrocytes are potentially able to express virtually all types of known neurotransmitter receptors [149]. Multiple studies have demonstrated that astrocytes, upon different stimuli, are able to considerably change their morphology and alter the expression of proteins involved in all aspects of cellular activity. Astrocyte plasticity and heterogeneity have been demonstrated not only in vitro or in reactive astrogliosis conditions, but also in the healthy brain [36,257,258,259]. Such plasticity suggests that astrocytes may be relatively easily manipulated to acquire desired properties, and is a strong argument for using astrocytes as cell targets in neuroregeneration therapies.

#### 3.1.2. Neurogenesis by Astrocytes in Neuropathologies

Astrocytes obtained from the CNS parenchyma after traumatic or ischemic brain injury form multipotent neurospheres much more efficiently compared with astrocytes from uninjured parenchyma [43,153,260,261,262]. However, usually only about 1/3 of neurospheres generated from reactive astrocytes of the adult mouse cortex are able to produce neurons [43,153]. Moreover, compared to neurospheres from NSCs, neurospheres originating from reactive astrocytes are less numerous, smaller in size and grow slower [262]. The fraction of neuron-generating neurospheres produced by astrocytes from an injured cortex was much higher (80%) in a study where very young mice were used for the injury model (perinatal hypoxia) [261]. Unexpectedly, the fraction of tripotent (producing astrocytes, neurons, and oligodendrocytes) spheres was found to be much higher in reactive astrocyte-derived cultures than in cultures from NSCs [262].

Notably, some NSC and RG markers are absent in resting astroglial cells in the adult brain, but are expressed in reactive astroglia [153,165]. It has long been proposed that in pathological conditions, some reactive astrocytes may undergo partial dedifferentiation and/or return to an earlier developmental state, based on the upregulation of some particular factors in these cells; namely, nestin [260,262,263,264], RC2 antigen [262] (which had been shown to be a posttranslational modification of nestin [265]), GFAP, vimentin, tenascin-C [260], Olig2 [266], SOX2 [262], or DCX [267]. Genome-wide expression analysis comparing embryonic RG, adult NSCs, and reactive and quiescent astrocytes showed that some genes related to neurogenesis (*CD24*, *Sox4*, *Sox11*, *Ptbp2*) become re-expressed in astrocytes as a result of their activation. However, in reactive astrocytes, these genes are still expressed at much lower levels compared with RG and NSCs [153].

A number of studies showed that the neurogenic program in astrocytes is activated specifically by invasive brain injuries. It seems like brain hemorrhage is an important factor for this. Buffo et al. used genetic or viral lineage tracing of mature astrocytes in the adult mouse cerebral cortex and showed that some astrocytes acquire stem cell properties after stab wound injury. After the injury, more than 1/3 of labeled astrocytes incorporated BrdU, indicating their proliferation. Most proliferating astrocytes remained within their lineage and continued expressing astrocyte-specific markers in vivo. In vitro, about 5% of labeled astrocytes that were successfully isolated by FACS from the injury site (but not from the intact cortex) formed self-renewing neurospheres that generated neurons, astrocytes, and oligodendrocytes in differentiation conditions. The authors could also show that astrocytes proliferating after the injury are not derived from the neurogenic SVZ [260]. Shimada et al. used GFAP-CreER^TM^;tdRFP mice, expressing the tdRFP marker in cells in which the GFAP promoter was active at the time of Cre induction, to confirm that at least a part of cortical cells that produce neurospheres after stroke are derived from reactive astrocytes (Rad-NSCs). The SVZ origin of these cells was excluded by showing that at the studied time point, 3 days post-stroke, SVZ-derived neuroblasts are absent in the peri-infarct area; brain regions adjacent to the neurogenic niches were excluded during the preparation of primary cultures. Cultured Rad-NSCs were able to produce neurons. However, upon transplantation into different brain regions (including neurogenic niches) of healthy adult, early postnatal or embryonic (E15) mice, the neuronal differentiation of Rad-NSCs in vivo was not observed. Thus, the environment in the brain, even in the neurogenic regions, does not support the neuronal differentiation of Rad-NSCs. However, transplanted Rad-NSCs proliferated and could produce both astrocytes and oligodendrocytes. In situ, tdRFP+ reactive astrocytes were shown to remain within their lineage after stroke; if these cells proliferated, they produced exclusively astrocytes [262]. Sirko et al. compared different neuropathology models in mice and showed that only invasive injuries (stab wound or cerebral ischemia) cause reactive astrocytes to become able to form self-renewing multipotent neurospheres in vitro; noninvasive damaging factors (chronic amyloidosis or induced neuronal death) do not cause a similar increase in astrocyte plasticity. SHH signaling was shown to be necessary and sufficient to elicit this stem cell response in astrocytes in vitro and in vivo [43]. More recently, the same research group compared different neuropathologies in humans. It was found that cerebral cavernoma (CCM, a condition with intracerebral hemorrhage), but not intracranial meningioma (ICM, not involving hemorrhage), induces astrocyte proliferation and increases their stem cell potential (proportion of astrocytes capable of neurosphere formation). CSF isolated from patients with CCM, but not ICM, increased neurosphere formation rate and the proportion of multipotent neurospheres when added to the medium, suggesting that astrocyte proliferation in the CCM is regulated by secreted factors. A proteomic analysis of CSF samples showed that multiple proteins enriched in CCM are involved in proliferation, neurogenesis and nervous system development. Galectin-3 and galectin-3-binding protein were identified as key components of a signaling pathway mediating astrocyte proliferation and neurosphere formation. Increased galectin-3 expression in astrocytes and their proliferation were also demonstrated in samples from different pathologies involving intracerebral hemorrhage: CCM, TBI, stroke and epilepsy with intraparenchymal hemorrhage (IPH). Importantly, in this study, astrocytes isolated from different parts of epilepsy brain samples were compared for their neurosphere-forming capacity. Cells from gliotic regions with IPH generated more neurospheres in total and more multipotent neurospheres as compared with cells from non-gliotic regions and cells from gliotic regions without IPH [268].

The striatum seems to be a brain region where injury-induced neurogenesis by astrocytes is triggered particularly easily. Magnusson et al. showed in vivo that stroke elicits a latent neurogenic program in striatal astrocytes in mice [269] (this paper is described in detail in Section 4.8). Similar observations were made by Duan et al., who traced the fate of local reactive astrocytes by injecting the pGfa2-eGFP plasmid into the rat striatum immediately following stroke induction. After 3 days, 47% of GFP+ cells bordering the ischemic core stained positive for nestin. Some of these cells expressed stem cell markers SOX2 or PAX6. An analysis of further time points showed that labeled cells showed time-dependent morphological changes along with the expression of proteins specific for neuronal lineage: NeuN, beta-tubulin class III (Tubb3), and microtubule-associated protein 2 (MAP2). GFP+ cells showing neuron-like morphology could be observed as early as 7 days post-stroke. At later stages (2–4 weeks), up to 14% of GFP+ cells co-expressed MAP2. At 13 weeks, the expression of cholinergic (ChAT) or GABAergic (GAD67) neuron markers was registered in GFP+ cells; these cells also stained positive for D2L dopamine receptor and NR2 glutamate receptor subunit, suggesting their ability to receive presynaptic inputs. At the same time, synapse formation by GFP-labeled neurons was shown by electron microscopy, and their integration into circuits was confirmed by patch-clamp recordings [270]. More recently, Kremer at al. reported that the gain of stemness in striatal astrocytes following ischemic injury involves the reprogramming of the astrocyte methylome to a stem cell methylome; this response is blocked by the absence of de novo methyltransferase DNMT3A [255]. A few studies of striatal neurogenesis were performed using the quinolinic acid (QA) excitotoxic lesion mouse model of HD. Nato et al. reported Ki67+/ASCL1+/SOX9+ TAPs and DCX+ neuroblasts that were present in the striatum as early as 2 weeks post-QA lesion and could be still observed at 6 months post-lesion. These cells were initially organized in clusters (neurogenic foci) that later dispersed as the cells underwent neuronal differentiation. Genetic lineage tracing confirmed that these cells originate from EAAT1 (GLAST)-expressing astrocytes; in GLAST-CreER^T2^ mice, the expression of the recombination marker YFP in striatal neuroblasts appearing after the QA lesion was observed if Cre induction was performed either before or after the lesion. In Nestin-CreER^T2^ animals, YFP labeling in these neuroblasts was much higher if Cre induction was performed after the lesion, suggesting that in response to injury, striatal astrocytes upregulate the expression of nestin, similarly to NSCs when they become activated. In addition, viral tracing was performed with injections of an AV vector, expressing Cre recombinase under GFAP promoter, into the striatum of Cre-reporter mice. These experiments confirm that QA-induced neurogenesis by GFAP+ astrocytes happens locally in the striatum. When the same virus was injected into SVZ, very few QA-induced neuroblasts were labeled [159]. Using the same injury model and 3D reconstruction techniques, Fogli et al. showed that the neurogenic foci forming in the lesioned striatum are transient structures continuously generated by the stochastic activation of neurogenic astrocytes. The potential of neurogenic foci induction is widespread in the striatum, with a higher probability in its medial domain. At least in the first weeks after the injury, the number of neurogenic foci remains stable, and the genesis of new foci is balanced by the exhaustion of old ones. An estimated lifetime of one focus is 10.6 days. Maturation profiles of different foci are spatially independent [271]. The same research group used the QA model to thoroughly study newborn neurons (neuroblasts) generated by murine striatal astrocytes following excitotoxic lesions (STR-nbl). It was found that these cells are transient (the number of BrdU+/DCX+ cells in the striatum dropped by about 80% between 6 weeks and 9 weeks post-lesion, while the number of BrdU+/DCX− cells remained stable). STR-nbl keep expressing DCX throughout their entire life; they can attain complex morphologies and have dendritic spines, but do not show distinguishable axons. Patch-clamp experiments with slices showed that these cells exhibit electrophysiological features typical of newborn neurons and, despite their short lifespan, integrate into brain circuits where they preferentially receive excitatory inputs. Monosynaptic retrograde tracing demonstrated that STR-nbl receive long-distance glutamatergic inputs. Most inputs originated from regions projecting to the medial striatum, consistent with the position of STR-nbl. scRNA-Seq revealed that these cells are not related to adult striatal neuron types and belong to the LGE-MEIS2/PAX6 GABAergic interneuron class, existing during embryonic and early postnatal development. The only adult neuron type belonging to this class is olfactory bulb interneurons generated in the V-SVZ. Indeed, in the scRNA-Seq data, STR-nbl clustered together with immature GABAergic neurons from the olfactory bulb [272]. Another disease model in which striatal neurogenesis has been studied is the slow progressive degeneration of striatal neurons (Creb1^Camkcre4^Crem^−/−^ mice, which also show slow neurodegeneration in the hippocampus). Similarly to acute striatum lesions, in this model, new neuroblasts, originating both in SVZ and in the striatum, were registered. The migration of SVZ-derived neuroblasts into the striatum was shown with intraventricular tracer injections. Neuroblasts generated locally in the striatum were associated with proliferating parenchymal cells with features of intermediate neuronal progenitors, such as clustering, the expression of EGFR and the expression of some other markers. Newborn neurons had a short lifespan, and their survival and maturation were severely impaired: between 15 and 28 days after BrDU injections start, the number of neuroblasts co-labeled with BrdU and at least one tested neuronal marker decreased by more than 90%. Only 3.8% of neuroblasts labeled at 15 d reached a relatively mature stage of differentiation (CaMKII-Cre+/NeuN+/DCX−) by 28 d. These neurons did not replace medium spiny neurons or express transcription factors involved in their differentiation. This failure to differentiate was cell-autonomous and not a result of environmental cues; in the same mutant mice, transplanted embryonic striatal precursors differentiated into DARPP32+ medium spiny neurons [273].

Taken together, these studies demonstrate that astrocytes may acquire NSC-like properties and produce neurons in some specific conditions; it happens after brain lesions caused by invasive injuries accompanied with hemorrhage. It was observed both in the cortex and in the striatum; however, in the striatum, the neurogenic response of astrocytes to injuries is much more prominent.

### 3.2. Neurogenic Potential of Oligodendrocyte Precursors

There is much controversy about OPC ablity to produce neurons. It has been confirmed that these cells may be artificially induced to revert to multipotential NSC-like cells that produce neurons in vitro [274,275,276] (see also Section 4). However, the possibility of such transformation actually happening in the brain is disputed.

Tracing the fate of OPCs in vivo is difficult, because NG2 expression is downregulated as the cells undergo terminal differentiation [277]. It also should be noted that some subpopulations of astrocytes [278] and microglia/macrophages [216,279] express NG2, while pericytes express both NG2 and PDGFαR [247,280], which leaves the possibility for a wrong identification of the cell type. Different markers were used to label or isolate OPCs and study their neurogenic potential in early postnatal or adult age, in the intact or damaged brain, with varying results. It may be possible that different ways of labeling OPCs produce overlapping but different cell populations, and only a fraction of these cells are capable of producing neurons. However, among the papers cited below, some studies using the same reporter system, and in some cases even exactly the same transgenic strain, obtained opposing results. Thus, the ability of OPCs to produce neurons in vivo is not conclusively proven.

#### 3.2.1. Neurogenesis by OPCs in the Healthy Brain

A few studies using *Ng2* [222,281,282] or *Olig2* [283] gene promoters as Cre recombinase drivers showed no evidence for neurogenesis by OPCs. However, Robins et al. performed NG2-CreER-based fate tracing (using the mouse strain generated by Zhu et al. [282]) and observed some reporter-positive neurons in the hypothalamus [284], even though Nishiyama et al. later interpreted this result as a spurious activation of Cre (due to transient *Ng2* gene expression) in mature neurons [208]. In their paper, Robins et al. argued that the results of their tracing are not an artifact because there were no labeled cells 2 days after the start of tamoxifen induction of the CreER, and the number of labeled neurons steadily increased over time between 7 and 60 days after the first tamoxifen injection [284]. Huang et al. generated a different NG2-CreERT2 mouse line and assessed labeled cells in mice of different ages. They registered the reporter expression in a small population of cortical neurons starting from the second postnatal week. However, they excluded the origin of these neurons from OPCs based on the very early appearance of the reporter in these neurons and the absence of BrdU in them. Spurious Cre expression in neurons was suggested as an explanation of this result [285].

The promoter of the *Pdgfra* gene (encoding PDGFαR) was also used as a Cre expression driver in OPCs. In the work by Rivers et al., who used this promoter, reporter-positive neurons were observed [286]. However, later, the same research group showed that these neurons have been wrongly attributed as the OPC progeny. Clarke et al. used the same PDGFαR-CreER mouse strain together with the 5-Ethynyl-2′-deoxyuridine (EdU) labeling of proliferating cells, and showed that OPCs within the postnatal forebrain do not produce neurons. It was found that all OPCs in the forebrain are mitotically active and incorporate EdU, while all neurons labeled by the Cre recombination reporter were EdU-negative and so could not originate from EdU+ OPCs [287]. Kang et al. used an independently generated strain of PDGFαR-CreER transgenic mice, and also did not observe neurogenesis by OPCs (a small number of neurons expressed the reporter, but the expression started too early and the number of labeled neurons did not increase with time) [288].

Guo et al. used the *Plp* gene promoter to drive Cre expression in OPCs; this gene encodes the myelin proteolipid protein, which in the postnatal mouse brain is expressed only in oligodendroglial lineage cells. The authors showed that in the early postnatal period, labeled cells generated small numbers of immature neurons in a few brain regions [289]. In a following study by the same group with the same labeling strategy, it was shown that OPCs of adult mice differentiate into pyramidal glutamatergic neurons in the piriform cortex [290].

A few studies where OPCs were isolated using a reporter expression driven by the promoter of the *Cnp* gene (encoding CNPase, an early oligodendrocytic transcript) showed that these cells may differentiate into neurons both in vitro and in vivo. Nunes et al. used the CNP-GFP reporter system in a plasmid and transfected with it cells from human brain samples to identify and sort OPCs. An independent method of OPC isolation in this work was sorting A2B5-positive cells; these cells were xenografted to fetal rat brains in experiments in vivo, where their progeny was identified by the expression of human nuclear antigen (HNA). In the rat brains analyzed at the age of P30, the transplanted cells gave rise to neurons, oligodendrocytes and astrocytes in a site-specific manner. It should be noted that A2B5+ cells in this study were considered fully identical to cells with an active *Cnp* promoter, while in fact these cell populations may only partially overlap [219]. Belachew et al. used the CNP-GFP reporter system in transgenic mice, studying the differentiation of OPCs in situ in the hippocampus of these animals. They demonstrated that the progeny of OPCs may differentiate into functional GABAergic neurons. Importantly, in this work, it was shown that most, but not all, cells expressing GFP also express NG2 [291]. Later, the same research group showed that early postnatal NG2+/EGFP+ cells in the SVZ of CNP-EGFP mice are mitotically active and express markers of multipotent precursor cells of the adult SVZ. When these cells were isolated by FACS and injected i.c.v. to WT mice of the same age (P2), EGFP-labeled cells were shown to migrate to the hippocampus and give rise to DLX+ GABAergic interneurons [292]. However, later, Tognatta et al. showed that the *Cnp* promoter may not be exclusively active in the oligodendrocytic lineage, because with CNP-Cre mice, false positive results may be observed depending on the reporter mouse line used. When CNP-Cre mice were crossed with the ROSA26/YFP Cre-reporter line, multiple reporter-expressing pyramidal neurons in the piriform and dorsal cortex were registered. In contrast, when Z/EG reporter mice were used, only oligodendroglial cells were found to be reporter-positive. Using the BrdU pulse-chase method to identify the birth time of the labeled cells, the authors showed that YFP+ neurons in CNP-Cre:ROSA26/YFP mice originate from embryonic RG cells. It was suggested that RG transiently upregulate the expression of some oligodendroglial genes, including *Cnp*; it may be sufficient to recombine the Cre-inducible reporter gene in highly sensitive reporter lines. This conclusion was corroborated by the results obtained with NG2creER^TM^BAC:ROSA26/YFP mice. It was shown that in this mouse strain, if Cre induction is performed at E16.5, transient *Ng2* promoter activation in RG results in the YFP labeling of RG-derived neurons. When tamoxifen administration and Cre induction were performed at P6, no YFP+ cells with neuronal morphology were found in the adult brains of these mice [293].

#### 3.2.2. Neurogenesis by OPCs in Neuropathologies

Even if OPCs do not normally produce neurons, it is conceivable that they may switch to neuronal fate in pathological conditions. Again, different studies reported opposing results related to this hypothesis. It is possible that the neuronal populations generated by OPCs after an injury are very small and/or transient.

Buffo et al. studied the mechanisms of reactive gliosis after a stab wound in mouse cortex. To test the capacity of OPCs near the injury site to become multipotent, these cells were isolated by FACS (using NG2 or PDGFRα surface antigens) and tested for neurosphere formation. No multipotent neurospheres were obtained from OPCs (in contrast to astrocytes, as described in Section 3.1.2) [260]. This is consistent with the results of Dimou et al., who used the Olig2::CreER^TM^ reporter system and did not register any cells with neuroblast or neuronal antigens among reporter-positive OPC progeny after a stab wound injury [283]. Similarly, Nato et al., who studied induced neurogenesis in the striatum in a model of HD (see Section 3.1.2), did not register neuroblasts originating from OPCs (the NG2-CreER^T2^ line was used to trace the progeny of these cells) [159]. In a stroke model, Magnusson et al. also did not observe neurogenesis by OPCs using the NG2-CreER; R26R-YFP mouse line [269].

However, other studies showed that upon CNS lesions, OPCs may become neurogenic, at least to some extent; some of these studies show that OPCs initiate but do not complete the neurogenic program, or that the neurons that they produce do not survive. Tai et al. confirmed that DCX+ cells appearing near the lesion sites in a mouse model of spinal cord injury (SCI) are derived from OPCs, using the Pdgfra-CreER™;Rosa-YFP mouse strain for lineage tracing. In addition, most of these cells stained positive for Olig2 or NG2. In total, 28.6% of these DCX+ cells also expressed ASCL1. However, the genetic tracing of ASCL1+ cells in Ascl1-CreER^T2^;Rosa-tdT knock-in mice after SCI showed that these cells failed to generate mature neurons. Very few tdT-labeled cells survived at 4 weeks post-injury, and these were predominantly co-labeled with NG2 (85.2%) or Olig2 (86.9%). Further experiments showed that an overexpression of SOX2 may be used to promote neurogenesis by OPCs in the damaged spinal cord (described in Section 4.5) [294]. Honsa et al. used a model of focal cerebral ischemia in NG2creBAC:ZEG double transgenic mice that express EGFP in OPCs and their progeny. The phenotype of EGFP+ cells was analyzed at different time points after the injury. In sham-operated mice, EGFP+ cells in the cortex were identified only as OPCs or oligodendrocytes. In the injured cortex, EGFP+ cells that proliferated early after the injury gradually decreased their expression level of NG2; by 14 days post-injury, most of them were NG2-. Some of these cells started expressing astrocyte (GFAP, EAAT1/GLAST) or immature neuron (DCX) markers. At 7 and 14 days post-injury, a small percentage of EGFP+ cells displayed passive membrane properties and current densities of voltage-activated channels typical for neural precursors; these cells were also DCX+. However, it seems that neural precursors generated by OPCs did not survive long. In brain sections obtained one month post-injury, very few cells coexpressed EGFP with markers of mature neurons, including NeuN. Also, at 14 days post-injury, a few DCX+/EGFP+ cells expressing the apoptotic marker caspase-3 were found [295]. Using Pdgfra-CreER^T2^:Rosa26R-YFP mice and a model of multiple sclerosis (experimental autoimmune encephalomyelitis), Tripathi et al. showed that in this pathology, >90% of YFP+ cells in the spinal cord belong to the oligodendrocyte lineage. However, small fractions of GFAP+ astrocytes (1–2% of all YFP+ cells) and NeuN+ neurons (<0.02%) were also registered. In addition, 2–10% of YFP+ cells could not be identified by the set of antibodies used in the study (targeting markers of neurons, glia, neural progenitors, vascular and immune cells) [296]. Patil et al. studied cell therapy in a model of chronic spinal cord contusion injury. Human iPSC-derived spinal pre-OPCs were injected into injured spinal cords of athymic nude rats. At 10 weeks post-injury, glial scar ablation was performed, and 9 days after that pre-OPCs were transplanted. Transplanted human cells and their progeny were identified in rat spinal cords with antibodies against SC121 (human cytoplasmic marker) or HNA. At 12 weeks post-transplantation, pre-OPCs differentiated into neurons, oligodendrocytes and astrocytes. About 40% of transplanted pre-OPCs became NeuN+ neurons; this number was similar in groups with and without glial scar ablation. Moreover, some transplanted cells stained positive for markers of neuronal types regionally specific to the spinal cord—CHX10, a marker of V2a neurons, or Islet1, a marker of motor neurons. When the glial scar was not ablated, a higher percentage of the transplanted cells differentiated into CHX10+ or Islet1+ neurons; when the scar was ablated, more of the transplanted cells became oligodendrocytes (APC+ or SOX10+). However, no functional improvement was observed at 12 weeks after the treatment; the authors explain this by the shortness of the observation period and/or the low level of remyelination (estimated by measuring the expression of MBP) performed by the transplanted pre-OPCs [250].

In addition to neurogenesis, it was shown that acute brain injury stimulates the differentiation of OPCs into astrocytes [220,295,297,298]. Interestingly, after focal cerebral ischemia, some OPCs differentiate directly into reactive astrocytes without preceding proliferation [214]. More recently, single-cell analysis methods showed that after focal cerebral ischemia, a specific, transient subpopulation of “astrocyte-like NG2 glia” expressing genes typical for both OPCs and astrocytes emerges and contributes to glial scar formation [299].

### 3.3. The Role of Microglia in Neurogenesis

Most neuroblasts generated in the adult neurogenic niches undergo apoptosis soon after their birth. Microglia perform an important neurogenesis-related function of removing these apoptotic cells via phagocytosis. Notably, this role is performed by unchallenged (not reactive) microglia [55].

Microglia may promote neurogenesis after brain lesions by modifying the microenvironment of NSCs. One of the key mechanisms of that is the microglial secretion of IGF-1, which mitigates apoptosis and promotes the proliferation and differentiation of NSCs; microglia are also known to produce other neurotrophic factors [300,301]. In vitro, Butovsky et al. showed that activated microglia co-cultured with neural progenitors from mouse SVZ affected their proliferation depending on the microglia polarization phenotype. Microglial cells activated by IL4 or IFNγ stimulated neurogenesis and oligodendrogenesis, while inflammation-associated microglia activated by lipopolysaccharide blocked the production of neurons and oligodendrocytes. Interestingly, there was a difference in the morphology of neurons whose generation was stimulated by microglia activated by IL4 or IFNγ. It was also shown that in the case of IL4-activated microglia, an application of the anti-IGF-1 antibody is sufficient to block its effects on oligodendrogenesis, but not on neurogenesis [300]. In vivo, Thored et al. observed increased numbers of activated microglia in SVZ during neuroblast migration into the striatum a few weeks after stroke induced by tMCAO in rats. It was shown that some of these microglial cells expressed IGF-1 [301].

Microglial cells are capable of dedifferentiation and neurogenesis in vitro. In 2004, Yokoyama et al. reported that some cells in pure rat microglia cultures express markers of CNS precursor cells—nestin, A2B5 or O4—and that these cultures may produce cells resembling neurons, astrocytes or oligodendrocytes after manipulations with serum concentration in the medium. The high purity of starting microglial cultures was confirmed by flow cytometry detecting IB4 or CD11b microglia markers; in addition, vital staining with microglia-specific agents was performed to trace cell fate. Cell types generated by microglia in these conditions were identified by their morphology and the expression of specific proteins. Importantly, the result varied substantially depending on the lot of fetal bovine serum (FBS) used for culturing, suggesting the role of unidentified growth factors from the serum in this process. It was also observed that microglia-derived dedifferentiated cells often formed aggregates resembling neurospheres. However, in contrast to bona fide neurospheres obtained from cultured NSCs, these aggregates did not proliferate in response to epidermal growth factor (EGF) and did not express some proteins typical for NSCs [248]. Later, the same research group obtained similar results in vitro with NG2-expressing microglial cells extracted from either neonatal rat brain or from a brain injury site after a stab wound [302].

The generation of neurons by microglia in situ is very unlikely because microglia have a distinct embryonic origin [303]; they do not arise from NSCs and are classified as a part of the immune system, so the lineage distance from microglia to neurons is much further compared to other glial cells. We did not find any reports about microglia being naturally able to produce neurons in either healthy or damaged CNS where the neuronal identity and the microglial origin of the new cells were thoroughly confirmed. Kitamura et al., who studied the neuroprotection provided by transplanted microglia after stroke, observed very few cells that were labeled by the dye marking the injected cells and immunopositive for MAP2 at the same time; however, the authors did not analyze these cells by any other methods [304]. Wohl et al. showed that in adult rat retina, a specific subpopulation of microglia expresses markers of CNS precursor or immature cells (nestin, vimentin, NG2) and responds to injury by proliferation. However, within 8 weeks after the injury, no neuronal markers were detected in BrdU-labeled proliferating microglia [279].

Nevertheless, artificial microglia-to-neuron conversion in vivo is possible. Some examples of this are described in Section 4.4 and Section 4.6. An argument for this strategy as a potential treatment is that different brain lesions often lead to increased numbers of microglial cells in the damaged region.

## 4. Strategies and Factors Used for Neuronal Conversion of Different Cell Types

The studies referenced in the previous section imply that even if glial cells usually fail to generate neurons or transform into neurons upon injury, artificial manipulations to achieve these outcomes may be quite simple. Indeed, an inactivation or an overexpression of just one gene may be enough for such purposes.

While neurogenesis by astrocytes observed naturally in the brain involves their dedifferentiation into a stem-cell-like state (as described in Section 3.1), artificial manipulations may allow a direct reprogramming of astrocytes into neurons, without transitioning through a pluripotent cell state (reviewed in [252]). For designing therapeutic interventions, both astrocyte dedifferentiation into neural progenitors and their direct conversion into neurons may be considered useful.

A number of genes related to neurogenesis have been selected for cell reprogramming based on comparisons between mature glial cells and various cell types in adult or developing CNS known to produce neurons [242], early experiments with fibroblasts and stem cells [252,305], and astrocytes [306]. Reprogramming factors are often “pioneer” TFs that have the ability to access closed chromatin, recruit facilitating factors, and initiate downstream gene expression programs for a new cell identity [307]. In some cases, one-step reprogramming is possible; nevertheless, it should be noted that the overexpression of pioneer TFs often causes the generation of cells that remain in an immature or hybrid state, and additional steps may be needed to fully differentiate the cells toward the target cell type [308].

Different starting cell types and combinations of factors to generate neurons have been tested in experiments in vitro; some of these are summarized in Table 1. In the text below, we mostly describe experiments in vivo, where the starting cell type was resident astrocytes or OPCs. Currently, the most commonly used strategies of inducing neurogenesis in vivo are the overexpression of *Ascl1*, *NeuroD1*, *Sox2*, *Ngn2* or *Dlx2*, and the downregulation of *Ptbp1* or *Notch1* genes (described in corresponding Section 4.3, Section 4.4, Section 4.5, Section 4.6, Section 4.7, Section 4.8 and Section 4.9). Some other factors have been reported to trigger neurogenesis in vivo. For example, Buffo et al. were able to induce neurogenesis by suppressing *Olig2* expression after brain injury (even though the cell type that produced new neurons was not clearly identified) [266].

Factors that promote NSC-like cell proliferation do not necessarily promote neurogenesis; they may favor self-renewal and/or gliogenesis by these cells instead, as was shown for Notch1 [309,310] or Olig2 [266]. In some cases, a two-step strategy may be required where the first stage proliferation and/or first steps of neuronal differentiation are stimulated by one factor, and then the maturation of iNs is aided by other factors [311]. Secreted neurotrophic factors like BDNF, EGF or FGF may be added to the medium [312,313], injected into the brain [314] or co-expressed with TFs [207,311] to increase cell survival and neuronal differentiation efficiency; these factors have been effective not only in transdifferentiation experiments, but also in experiments with NSCs. Another way to improve the transformation outcome is the co-expression of *Bcl2*, apoptosis and ferroptosis inhibitor [315]. It was also shown that astrocyte-to-neuron conversion may be facilitated by the upregulation of some genes encoding neuron-enriched mitochondrial proteins [316].

Combinations of small-molecule drugs that target intracellular signaling pathways or epigenetic mechanisms may be used instead of TFs to induce neuronal transdifferentiation; however, there are not many studies where this approach was used in neuropathology models [317], so we will not describe these methods in detail. Drugs may also be used to aid glia-to-neuron conversion initiated by TFs. For example, valproic acid (VPA) was demonstrated to promote the generation of iNs initiated by different protocols from both astrocytes [311] and OPCs [276].

So far, the reprogramming of glial cells in neuropathology has been mostly explored in models of stroke, PD, TBI or SCI, with at least one study [318] using an AD model (reviewed in [319,320]). Studies of this approach in models of epilepsy are still few, even though promising (described in Section 5).

**Table 1:** Selected studies where neuronal transdifferentiation was induced in vitro using different starting cell types and transformation strategies. The table is sorted by the starting cell type.

Starting Cell Type	Resulting Cell Type; Neuronal Subtype Markers	Reprogramming Factor	Expression Vector	Transformation Efficiency *	Proofs of the Neuronal/NPC Identity of Manipulated Cells	Comments	Reference
Human or mouse established cell lines: HeLa, NT2, N2A, ARPE19 Primary mouse embryonic fibroblasts (MEF)	Neurons (subtype not specified)	*PTBP1* knockdown by shRNA	LV (pLKO.1 with U6 promoter)	~20–25% for N2A, ~10–15% for MEF	Neurite outgrowth, expression of Tubb3 in all transduced cell lines (even HeLa) MAP2, NeuN, synapsin I, VGlut1 expression in N2A-iNs and MEF-iNs Neuron-like ion channel currents, APs in N2A-iNs and MEF-iNs; EPSCs/IPSCs in MEF-iNs	A slow growth phenotype of transduced cells was noted. The efficiency of two different shRNAs was different.	[321]
Human iPSCs	Neurons (no high transcriptomic similarity to reference subtypes) Most expressed CUX1 and VGlut1, but not GAD1/2	Ngn2, different dosages	Stable cell lines with Dox-inducible *NGN2* expression were generated using LV	Not specified	Expression of Tubb3, PRPH Neuron-like gene expression profiles	Four transcriptionally distinct cell populations were identified, one of them not neuronal. The resulting cell type depended on *NGN2* expression level and duration.	[322]
Mouse primary embryonic, early postnatal and adult fibroblasts	Neurons (subtype not specified) Some expressed VGlut1, GABA, or calretinin	ASCL1 alone or in combination with other factors	LV (19F-pool), Tet-On system	Up to 19.5% for ASCL1 + BRN2 + MYT1L	Expression of Tubb3, MAP2, NeuN, synapsin Expression of TauEGFP (in the used mouse strain only neurons express this reporter) Neuron-like ion channel currents, EPSCs/IPSCs		[305]
Mouse primary embryonic fibroblasts; primary postnatal cortical astrocytes	Midbrain dopaminergic neurons expressing TH, Pitx3, Lmx1a, Engrailed-1, Aldh1a7, Foxa2, Vmat2, Msx1, Girk2, Slc6a3 (protein or mRNA expression was shown)	ASCL1 + LMX1B + NURR1	LV with CMV promoter and tetracycline response element	14.9% for fibroblasts, 35.1% for astrocytes	For fibroblast-iNs: expression of Tubb3, large Na^+^ currents, APs For astrocyte-iNs: expression of Tubb3, synaptophysin; neuron-like ion channel currents, passive membrane properties, EPSCs/IPSCs, APs, Ca^2+^ oscillations; dopamine release in response to depolarization	Expression of multiple cell-type-specific transcripts in iNs was confirmed by RT-qPCR. Dopamine release was quantified by HPLC. Efficiency of transformation for cell type-specific marker TH is also specified: 9.1% for fibroblasts, 18.2% for astrocytes.	[323]
Rat primary neonatal cortical astrocytes	NPCs; mature neurons not generated	Oct4 + SOX2 + KLF4	LV, Tet-On system	Not specified	Downregulation of GFAP, S100b, Sox9 Expression of synapsin I, nestin, NeuN, Tubb3	Many cells remained in a transitional state co-expressing astrocytic, neuronal, and progenitor markers.	[324]
Primary dorsal midbrain astrocytes from P5–P7 mice	Glutamatergic (VGlut2+) or GABAergic (GABA+, GAD67+, VGAT+) neurons	ASCL1	LV	76.8%	Neuron-like morphology Expression of Tubb3, MAP2, synapsin I Neuron-like ion channel currents, passive membrane properties, EPSCs/IPSCs, APs		[325]
Mouse primary astrocytes from neonatal cortex	Neurons, predominantly GABAergic (GAD67+, GABA+)	NeuroD1	AAVShH19 serotype (high astrocyte transduction) with Cre-FLEX system AAV-CMV-NeuroD1-T2A-GFP	>90%	Neuron-like morphology Decrease in GFAP expression Expression of Tubb3, MAP2	Generation of mostly GABAergic iNs contrasts with many previous reports about NeuroD1; the developmental stage of astrocytes may be the key difference	[326]
Primary astrocytes from neonatal or adult mouse cortex, cerebellum or spinal cord	Neurons (mostly glutamatergic VGlut1+ from Ngn2-induced cells, mostly GABA+ GABAergic from ASCL1-induced cells)	Ngn2 ASCL1	LV	For neonatal astrocytes: 50% for cortex, 30% for cerebellum, 20% for spinal cord For adult astrocytes: <15% for all regions	Neuron-like morphology Expression of Tubb3, MAP2, NeuN		[327]
Mouse primary astrocytes cultured without growth factors to minimize their proliferative potential	Neurons (subtype not specified)	NeuroD1	LV with CAG promoter	1%	Minimal Tubb3 expression	Very limited efficiency compared to reactive/proliferating astrocytes	[28]
Commercially available line of human cortical astrocytes (whole culture or sorted CD44+ cells)	NSCs/neural progenitors that may produce neurons, astrocytes and oligodendrocytes	Oct4, SOX2, or Nanog; ASCL1 to promote the neuronal phenotype	LV (pSIN with EF1a promoter)	Up to 90% of transduced cells expressed NSC markers. When NSCs derived from astrocytes were cultured in neuronal differentiation medium, 41.4% expressed neuronal markers	For NSC: typical cell and colony morphology; response to EGF, FGF; expression of Sox2, Pax6, CD15, CD133, nestin; decreased expression of astrocytic and pluripotency markers CD44, SSEA-3, Tra-1–60; clones could be passaged; NSC-like gene expression (microarray analysis) For neurons generated by NSCs: neuron-like morphology, immunopositivity for Tubb3, Map2, NF, synapsin, GABA, GAD67, response to NMDA and kainate, decreased response to ATP	Expression of each TF was sufficient to convert astrocytes into NSCs without passing through a pluripotent stage. Nanog was the most efficient TF.	[312]
Human fetal astrocytes, fetal NPCs (commercially available lines)	No success in generating iNs	NeuroD1	AAV-5 Cre-GFAP-FLEX NeuroD1-GFP	Not applicable	Not applicable	The authors observed unexpectedly heterogeneous cells in the starting cultures	[328]
Rat primary OPCs from adult neocortical gray matter	Glutamatergic pyramidal neurons (subtype was defined based on morphology and electrophysiological properties)	Ngn2, NeuroD1, ASCL1 + DLX2 or PAX6	Retrovirus with CAG promoter	50% for Ngn2, 50% for NeuroD1, 15% for ASCL1 + DLX2; PAX6 did not induce neuronal conversion	Neuron-like morphology Expression of Tubb3, MAP2, NeuN, synaptophysin Upregulation of neuronal transcripts (DCX, Grin1, NeuroD1), downregulation of Olig2 and O4 Presence of neuron-like ion channel currents, single and repetitive APs	Comparison of transduction efficiency and neuronal conversion efficiency suggests that only fully reprogrammed cells survive. Experiments in vivo additionally confirmed the glutamatergic identity of iNs by marker (CaMKII, Tbr1) expression	[329]
Primary microglia, astrocytes or oligodendrocytes from neonatal mouse cortex (oligodendrocytes were differentiated in vitro from primary OPCs)	Neurons, 74.2% VGlut1+ glutamatergic and 25.7% GAD67+ GABAergic	8 TFs (ASCL1, BRN2, MYT1L, Olig2, ZIC1, SOX2, Ngn2, NeuroD1), miR124 and shRNA targeting epigenetic factor MBD3 were screened; NeuroD1 was selected for further experiments	LV with tetracycline response element for factor screening For targeted NeuroD1 expression in microglia, LV with Iba1 promoter was used	>30% for NeuroD1 in either microglia or oligodendrocytes	Expression of Tubb3 Neuron-like transcriptome (RNA-Seq) Presence of neuron-like ion channel currents, passive membrane properties, EPSCs/IPSCs, single and repetitive APs, Ca^2+^ oscillations Integration of iNs into existing neural circuits in primary neuronal cultures	Resting astrocytes cultured without EGF/FGF-2 could not be efficiently converted by NeuroD1. The authors showed that the presence of bivalent chromatin domains at neuronal genes predicts cell type susceptibility to NeuroD1-mediated reprogramming.	[330]
Primary microglia or astrocytes from neonatal mice	No success in generating iNs	NeuroD1	LV with CAG promoter	Not applicable	Absence of neuronal markers; microglia-specific gene expression did not change Microglia expressing NeuroD1 did not transform into neurons but instead underwent cell death (confirmed by live-cell imaging and apoptosis assays)	Results reported in [330] were not reproduced	[331]
Primary microglia or resting astrocytes from neonatal mouse cortex	Neurons (subtype not specified)	NeuroD1 (different expression levels) alone or with ASCL1 and BRN2	LV, Tet-On system	>60% for microglia or resting astrocytes with the highest NeuroD1 level (repeated infections)	Expression of Tubb3	Increasing NeuroD1 expression dramatically improved conversion efficiency even in resting astrocytes. The addition of ASCL1 and BRN2 facilitated the conversion of both microglia and astrocytes when NeuroD1 expression level was low.	[332]
Primary microglia from neonatal (P4) cortex of Hexb-CreERT2::Stop-YFP mice	Neurons (subtype not specified)	NeuroD1	LV, Tet-On system	<20% for NeuroD1; >50% for NeuroD1 with the drug cocktail	Expression of Tubb3, MAP2	The used mouse strain allowed the specific labeling of microglia. The drug cocktail used to facilitate the conversion: VPA, forskolin, and Y-27632.	[333]
Microglial lineage cells generated from human ESC	Neurons (subtype not specified; transcriptomic profile resembled excitatory neurons)	Ngn2	Stable cell lines with Dox-inducible *NGN2* expression were generated using CRISPR–Cas9 gene editing	>40%	Neuron-like morphology Expression of Tubb3, DCX, MAP2, NeuN, synapsin I Loss of CD235, SOX9, GFAP, Olig2 Neuron-like gene expression profiles Neuron-like ion channel currents, passive membrane properties, APs	<10% transformation efficiency in vivo (in cell grafting experiments)	[334]

* There is no strict definition of transformation efficiency. In the studies referenced here, it was calculated as the percentage of cells expressing either neuroblast or pan-neuronal markers among all surviving treated (labeled) cells; in some cases among all surviving cells including non-transduced ones (when universal staining of nuclei was used for cell counting). Different neuroblast/neuron markers were used in different studies (as specified in the column “Proofs of the neuronal/NPC identity of manipulated cells”); in many cases, multiple markers were estimated, and only one of them was arbitrarily used for quantification. In some cases, marker expression was quantified but the wording “transformation efficiency” was not used for these numbers. The efficiency number does not say anything about how many cells survived. It is known that transitioning cells have a poorer survival rate than iNs. Thus, the transformation efficiency may be estimated higher if markers for more mature stages of neuronal differentiation are used. In conclusion, transformation efficiency numbers from different studies in this table cannot be directly compared.

### 4.1. Transcriptomic, Proteomic and Metabolic Changes in Glial Cells Subjected to Neuronal Conversion

Artificial neuronal reprogramming has its own patterns and stages, and understanding this process may help to make it more efficient. Studies cited below were performed for neuronal conversion starting with different cell types and induced by different factors, and in some cases comparisons with natural embryonal or adult neurogenesis were made. Importantly, natural neurogenesis always includes a proliferation stage, while a forced conversion of another cell type into neurons may or may not include proliferation.

Lin et al. started with human iPSCs and used Ngn2 to transform them into neurons. scRNA-Seq was performed at different time points, 0–28 days from the induction of the transformation. Four transcriptionally distinct cell populations were present among differentiating cells at day 28—three neuronal clusters and a mesenchymal cell cluster (off-target differentiation). Two clusters of iNs (Ngn2-iN) had a high expression of *PRPH*, which is characteristic of peripheral nervous system (PNS) neurons. The third neuronal cluster was marked by the expression of *GPM6A*, expressed in the developing CNS. Additional experiments with different monoclonal or polyclonal iPSC lines confirmed that the result of Ngn2-based neural reprogramming of iPSCs is intrinsically heterogeneous; the same four clusters were observed for each line. Transcriptome trajectories of Ngn2-iNs were compared with published transcriptomic data describing the development of neurons in brain organoids. Ngn2-iNs transitioned from pluripotency directly into neural precursor stages, skipping the intermediate stages observed in organoids. Unlike neurons in the iPSC-derived organoids, Ngn2-iNs did not show high transcriptomic similarity to any known neuron subtypes. Moreover, it was found that all Ngn2-iNs have mixed signatures of CNS- and PNS-derived neurons [322].

Metabolic and proteomic changes have been assessed in astrocyte-to-neuron transdifferentiation. Gascón et al. used continuous single-cell live imaging to monitor the neuronal reprogramming of mouse fibroblasts and astrocytes induced by an *Ascl1*-expressing vector. They found that 94% of transfected cells that did not acquire a neuronal morphology died, while among cells with neuronal morphology the death rate was only 48%. Further experiments showed that the metabolic change during neuronal reprogramming causes oxidative stress. The most important result of this work is the identification of a critical time point after which cells either successfully convert into neurons or succumb to cell death. Inhibitors of apoptosis or necroptosis increased cell survival but did not affect the proportion of iNs among the initially transfected cells. However, the inhibition of lipid peroxidation and ferroptosis by different methods, including treatment with antioxidants or overexpression of *Bcl2*, potently increased reprogramming efficiency. Bcl-2 accelerated the transition, which increased overall cell survival because in this model astrocytes have a higher death rate than iNs. Transcriptome analysis confirmed that treatments promoting neuronal reprogramming elicit an anti-oxidative stress response [315]. This finding was used later by some other scientists who added the overexpression of *Bcl2* to their chosen neuronal transformation methods to increase the transformation efficiency [335,336,337]. Russo et al. studied the process of astrocyte-to-neuron transformation induced by ASCL1, with particular attention paid to mitochondria. Primary astrocyte cultures were prepared from the cortex of P5 mouse pups and transduced with a retrovirus encoding mitochondrion-targeted GFP (*mitoGFP*) with or without *Ascl1*. It was shown that an elongated and ramified mitochondrial network in astrocytes changes into smaller mitochondria with shorter and rounder morphology in successfully reprogrammed iNs by 7 days post-infection (dpi). Liquid chromatography–tandem mass spectrometry was then performed to identify proteins enriched in the mitochondria of bona fide astrocytic or neuronal cultures, and a number of differentially enriched proteins were selected to be assessed by immunohistochemistry in cells at different stages of astrocyte-to-neuron transition. It was shown that mitochondrial proteins enriched in astrocytes (Sfxn5, CPOX) or neurons (Prdx2, GLS) change their expression relatively late during neuronal reprogramming, at 5–7 dpi. In subsequent experiments, the effect of an early artificial upregulation of genes encoding proteins enriched in neuronal mitochondria was assessed in the context of ASCL1-induced transformation. *Prdx2* and *Sod1* were identified as the genes whose induction improves reprogramming efficiency the most. *Prdx2* and *Sod1* co-activation sped up the conversion process and increased the lifespan of the converted neurons, but not of astrocytes failing to reprogram, consistent with the cell-type-specific role of antioxidants. Remarkably, the induction of genes encoding astrocyte-enriched mitochondrial proteins, even with antioxidant function (*Mgst1*, *Prdx6*), was not beneficial for reprogramming [316].

Interestingly, it has been shown that the mutant form of ASCL1, ASCL1SA6 (but not WT ASCL1), induces interneuron-specific hallmarks. Cooper et al. explored the mechanisms of this difference. MMLV-based retroviruses expressing *Ascl1* or *Ascl1SA6* with or without *Bcl2* were injected into the somatosensory cortex of early postnatal (P5) mice. Cells were isolated for scRNA-Seq at 4 dpi; at this stage some *Ascl1SA6/Bcl2* co-expressing cells already had more neuron-like morphologies. It was shown that transduced astrocytes and OPCs transitioned toward neuron-like cells by the sequential induction of neural progenitor genes (*Hey1*, *Hes5*, *Notch1*), markers of early neurogenesis (*Dll1*, *Sox11*, *Tubb3*), and markers of more mature neurons (*Rbfox3*, *Srrm4*, *Myt1l*). Astroglial transcriptomes were more effectively remodeled than those of OPC; transduced microglia did not upregulate neuronal markers. Interestingly, in some astrocytes ASCL1 induced partial reprogramming towards an OPC fate. Neuronal progression was most pronounced in *Ascl1SA6*-expressing cells, as compared with *Ascl1*-expressing populations; the co-expression of *Bcl2* increased conversion efficiency. Unexpectedly, *Ascl1SA6*, but not *Ascl1* expressors, lacked the expression of Dlx family genes, indicating that ASCL1SA6-iNs only partially recapitulate interneuron programs, failing to activate a key gene expression module. The co-expression of *Dlx2* with *Ascl1SA6* restored a more canonical interneuron-like transcriptome. At 4 dpi, *Ascl1SA6/Dlx2* co-expressors expressed DLX2 transcriptional targets, including genes important for interneuron migration (*Zeb2*, *Arx*). Microscopy performed at 2–4 weeks post-infection (wpi) showed that ASCL1SA6/Bcl-2-iNs accumulated in cortical layer 1, occupying positions atypical for cortical interneurons, while *Ascl1SA6/Dlx2* coexpression redirected iNs to more canonical positions in layers 2/3. In this study, DLX2 failed to induce neurogenesis on its own [337].

However, DLX2 was sufficient for neuronal transformation in the adult mouse striatum in a study by Zhang et al. They used a lentivirus (LV) vector to express *Dlx2*. Astrocyte-to-neuron transformation was confirmed by immunohistochemistry at different time points. The ratio of neurogenesis to gliogenesis by DLX2-induced tdT+ cells decreased with time (between 8 and 12 wpi); almost all iNs were GABA+. In a separate experiment, brain samples were prepared for scRNA-seq at 4 wpi. The sequence of transition stages was suggested by pseudotime analysis, with immunohistochemistry data as a reference. It was shown that Lenti-DLX2 astrocytes transition toward neuroblasts (expressing *Dcx* and *Calb2*) through a proliferating neural progenitor (NPC) intermediate stage (expressing *Cdk1*, *Mki67*, and *Ascl1*). The gene cluster that included neurogenesis genes showed the enrichment of putative DLX1/2 targets in the developing mouse brain. Astrocyte genes showed enrichment for metabolic Gene Ontology (GO) terms, suggesting a metabolic shift during the conversion. “Lenti-astrocyte” cluster were enriched for immune responses, a reaction to the LV infection. “Transition” genes were enriched for terms related to metabolism in mitochondria, indicating a switch to oxidative phosphorylation. NPC-enriched genes were dominated by genes involved in the cell cycle. Neurogenesis genes were enriched in terms related to neural development. A comparison with previously published transcriptomic data showed that DLX2-induced reprogramming resembles endogenous neurogenesis in both adult and embryonic stages; like adult neurogenesis, DLX2-induced neurogenesis starts from a stable quiescent cell state. Early downstream effects of DLX2 overexpression—upregulation of *Dlx1* and *Dlx5*, downregulation of *Notch1*—were confirmed to induce astrocyte-to-neuron transformation by themselves in separate experiments in vivo [338].

Ma et al., working with cultured human astrocytes and a *Neurod1*-expressing retroviral vector, described the transcriptomic changes throughout the 2-week astrocyte-to-neuron conversion process. GO analysis identified three distinct categories of genes highly upregulated during the reprogramming. The first group was related to virus infection, highly expressed at 1 dpi. The second cluster had genes involved in cell cycle and telencephalon development and was turned at 3–5 dpi. The third group contained genes expressed at the late stage of reprogramming; their GO categories showed neuronal gene clusters including “neuron projection” and “synapse assembly”. A network of TFs was activated within 24 h after the infection; these factors included many known NeuroD1 targets in embryonic stem cells. The upregulated TFs included the NeuroD family and factors involved in neurogenesis. At 3 dpi, the expression of NeuroD1 itself decreased. The downregulation of glial genes happened within 3 days of infection. Among the earliest downregulated genes were genes encoding non-voltage-dependent sodium channel subunits and a protein involved in Ca^2+^ signaling. This may reflect the switch to the predominant use of voltage-gated sodium and calcium channels characteristic for neurons. Curiously, some neurotransmitter receptor genes were only transiently upregulated at the early stage of conversion [339].

Papadimitriou et al. performed RNA-Seq of iNs derived from cultured astrocytes by the microRNA miR-124 at 7 days after the conversion initiation. It was shown that miR-124 alone downregulated multiple astrocytic genes (including some TFs) and upregulated multiple neuron-specific genes (including a number of TFs related to telencephalic development). Many genes downregulated by miR-124 were related to cell cycle, gliogenesis, and astrocyte differentiation. In the cells that were treated with both miR-124 and the neurogenic compound ISX9, many of the neuron-specific genes that responded to miR-124 were further upregulated by ISX9. GO enrichment analysis showed that miR-124 alone upregulated genes related to neuronal differentiation as well as genes related to neuronal functions (mostly associated with synaptic transmission), while ISX9 increased the number of upregulated genes related to more mature neuronal functions (like APs, axon development, and subtype-specific synaptic transmission). The addition of ISX9 increased the number of upregulated TFs implicated in telencephalic development and upregulated TFs related to other brain regions. A comparison of these results with publicly available scRNA-Seq data from an embryonic mouse cortex revealed that miR-124 initiates a transcriptional program characteristic of endogenous intermediate progenitor cells, while ISX9 enhances the neuronal differentiation of miR-124-iNs toward layer-specific neuronal gene signatures [340].

Zamboni et al. performed scRNA-Seq with WT and *Rbpj*-depleted (Notch signaling-deficient) astrocytes from the intact or injured mouse cortex. *Rbpj* depletion in astrocytes induced the upregulation of TFs such as *Ascl1*, *Ngn1*, and *Olig2* that promote neuronal specification during corticogenesis. Mutant astrocytes were also enriched in genes that are direct targets of ASCL1 and expressed anti-proliferative gene *Btg2*, a marker for neuron-generating progenitors in the developing CNS. The deletion of *Rbpj* in astrocytes also led to the downregulation of genes associated with NSC quiescence in adult neurogenic niches. This transcriptional profile resembles primed but still quiescent NSCs. The authors demonstrated that the neurogenic program in these cells only activates upon injury; *Rbpj*-depleted astrocytes close to the lesion do not transdifferentiate into neurons directly, but form TAPs before generating proliferative clusters of neuroblasts. Neurogenesis by these cells involved the downregulation of genes related to the astroglial functions (lipid and carbohydrate biosynthesis, glutamate and water transport) followed by the upregulation of ribosomal genes, genes implicated in chromatin modification, as well as genes implicated in neurodevelopment (*Dlx1*, *Dlx2*, *Sox4*). After this, the transient expression of cell cycle genes was followed by the induction of transcripts related to neuronal fate specification and axonogenesis. Post-mitotic neuroblasts generated by transformed astrocytes expressed TFs indicative of the GABAergic phenotype. At the latest maturation stages, they upregulated key GABAergic signaling components and genes implicated in synapse formation and maintenance. Comparison with previously published data showed that this process recapitulates SVZ neurogenesis [341].

We may conclude that different ways of inducing astrocyte-to-neuron transdifferentiation lead to mostly similar changes in gene expression, starting with the loss of astrocyte-specific transcripts and followed by the upregulation of genes associated with embryonal or adult neurogenesis, and later genes involved in neuronal function. It seems like the default neuronal type induced by artificial reprogramming by most key neurogenic TFs is GABAergic interneurons; however, in the case of TF overexpression without additional manipulations, their differentiation may be incomplete. The artificial overexpression of one of these TF would cause the upregulation of other such factors at early stages of transformation, so different strategies converge. At the same time, there are nuances in exact molecular mechanisms that may affect the end result, both the number and the type of iNs. Some reprogramming factors involve a TAP phase with additional proliferation, which may be useful in situations where direct transdifferentiation methods may lead to the local depletion of glia.

We should also mention the transdifferentiation of microglial lineage cells. In a recent work by Jin et al., these cells were used as a starting cell type for neuronal transformation by the overexpression of *Ngn2*. The authors performed snRNA-Seq of cells undergoing the transition and defined three main stages of gene expression changes: (1) suppression of immune lineage programs, (2) engagement of ECM remodeling and structural reorganization pathways, and (3) emergence of transcriptional networks supporting neuronal signaling, calcium handling, axonogenesis, and synaptic function. Interestingly, they identified a specific group of genes that represents a reprogramming barrier characterized by transcriptional conflict between myeloid and neuronal programs. It is enriched for stress-adaptive factors, neuronal repressors, chromatin remodelers associated with plasticity, and pro-neuronal and transcriptional organizing factors, suggesting that neuronal differentiation is temporarily restrained to allow for chromatin stabilization at the intermediate stages. Some cells enriched for this gene group reverted towards the primitive macrophage progenitor state, while others progressed toward neuronal commitment [334]. These new results are particularly interesting because microglia are rarely used in such experiments, even though microglia-to-neuron transformation is possible and may be important for some clinical applications.

### 4.2. Overexpression of Transcription Factor Paired Box 6 (Pax6)

The similarities between embryonic RG and astrocytes (discussed in Section 2.5 and Section 3.1) inspired the idea that astrocyte-to-neuron conversion recapitulating neurogenesis by RG is possible. In one of the earliest transdifferentiation studies, in 2002, Heins et al. identified PAX6, a TF that is expressed in RG and is crucial for patterning the telencephalon in embryogenesis, as a factor that may be used for astrocyte transformation. The authors confirmed the importance of this factor for the developmental neurogenesis by experiments with *Pax6* mutant mice. Then, they overexpressed *Pax6* in postnatal astrocytes in vitro using a retroviral vector. More than half of the transduced astrocytes generated clones containing exclusively neuron-like cells that stained positive for Tubb3, NeuN, GABA or glutamate. Notably, it was shown that the overexpression of *Pax6* caused the upregulation of Ngn2 and the downregulation of MASH1 (ASCL1) protein expression [342]; these proteins are important neurogenic TFs that were used for neuronal reprogramming in later studies.

### 4.3. Overexpression of Transcription Factor Achaete Scute Complex Like-1 (ASCL1)

ASCL1, also known as Mammalian Achaete-Scute Homolog-1 (MASH1), is a pioneer TF and one of the best-characterized proneural TFs. In mammals, *ASCL1* is expressed in progenitor cells already displaying neuronal commitment and functions as a modulator of subtype specification. ASCL1 drives the differentiation of the GABAergic lineage during development. It also participates in the maintenance of the NSC pool in adult neurogenic niches [343].

In 2010, Vierbuchen et al. tested the potential of fibroblasts to be transformed into neurons. The authors screened 19 different TFs that could be important for neuronal differentiation and made LV vectors expressing these factors. The combination of three factors—ASCL1, BRN2 and MYT1L (BAM cocktail)—was shown to transform fibroblasts into functional neurons. The expression of *Ascl1* alone was sufficient for the formation of neurons, but in this case iNs had a less complex morphology [305]. Torper et al. showed that a lentiviral overexpression of the BAM cocktail causes the transformation of murine striatum astrocytes into neurons in vivo [26].

Astrocytes are closer in lineage distance to neurons compared to fibroblasts; based on that, Heinrich et al. experimented with reducing the number of factors. A viral overexpression of *Ascl1* caused neuronal differentiation of 33.5% of postnatal cortical astrocytes in vitro. A coexpression of *Dlx2* increased this ratio to 93%; 6 out of 15 recorded cells showed specific interneuronal firing patterns [344]. Liu et al. showed that *Ascl1* overexpression by the AAV-GFAP-Ascl1_mCherry viral vector in the midbrain of mice aged 12–15 days leads to 93% colocalization of the mCherry marker with NeuN staining at 28–32 dpi. At 45 dpi, 13.2% of mCherry+ cells expressed the GABAergic neuron marker GAD67 (GAD1), while 6.5% expressed glutamatergic neuron marker VGlut2. The expression of neuronal progenitor markers (*En1*, *En2*, *Pax3*, *Pax7*) was not detected in mCherry+ cells by RT-qPCR at 4–30 dpi. At 30 dpi, iNs were able to generate APs and were integrated into neuronal circuits, showing the presence of excitatory and inhibitory spontaneous postsynaptic potentials. The authors also showed the colocalization of mCherry with NeuN after the virus was injected into the striatum or cortex of adult mice (the markers of neuronal types were not estimated) [325].

Torper et al. tried to generate dopaminergic neurons in murine striatum using the combination of AAV vectors expressing *Ascl1*, *Lmx1a*, and *Nurr1* (ALN cocktail), as had been proposed in previous studies [323,345]. However, this combination of factors did not induce the generation of dopaminergic neurons, but instead caused the transformation of both OPCs and astrocytes into glutamatergic or GABAergic neurons. Among iNs derived from astrocytes, 16% expressed VGlut1, and 68% expressed GAD65/67 (for OPC-derived iNs, these fractions are not reported). No iNs expressed tyrosine hydroxylase (TH), a dopaminergic neuron marker [346]. Pereira et al. also failed to transform OPCs into dopaminergic neurons by the ALN cocktail. The AAV-driven expression of this TF combination in the murine striatum caused OPCs to generate neurons, but mostly (41.27%) PV+. Infected (GFP+) cells expressing other markers (ChAT for cholinergic, NPY for persistent and low-threshold spiking or DARPP32 for striatal projection neurons) comprised less than 10% of all iNs each. At 12 wpi, the electrophysiological properties of most GFP+ cells were similar to those of fast-spiking PV+ interneurons. Interestingly, subjecting striatum OPCs to other reprogramming factor combinations also mostly yielded PV+ neurons (30–45% of GFP+ cells). GAD65/67+ cells comprised 9–27% of iNs in different conversion strategies; no VGlut1+ iNs were detected for any strategy [347]. A successful generation of dopaminergic neurons from astrocytes in vitro and in vivo in a murine PD model was demonstrated in the study by Rivetti di Val Cervo et al., where the TF cocktail of NeuroD1, ASCL1 and LMX1A was supplemented by microRNA miR218. The transformation efficiency was further improved by small molecules that promote chromatin remodeling and activate the TGFβ, SHH and Wnt signaling pathways [348].

A retroviral overexpression of *Ascl1* + *Sox2* was shown to induce the formation of neurons in the mouse cortex in a stab wound model. Heinrich et al. used GIN transgenic mice in which GFP is expressed in GABAergic neurons. At 23 dpi, processes of neurons apparently generated from infected glial cells (DsRed+) were surrounded by GFP+ endogenous inhibitory neurons. The colocalization of DsRed with GFP in the same cells was not assessed [349]. Faiz et al. performed a lineage tracing of SVZ-derived cells using Nestin-CreER^T2^;YFP^fl^ mice. They showed that after stroke, migratory SVZ cells give rise to reactive astrocytes at the lesion site. To convert these cells into neurons, the authors used AAV5-Gfap-Ascl1-T2A-mCherry virus injected 7 days post-stroke. In vitro, the conversion rate varied greatly between different cultures, being a little below 50% on average. In vivo, the conversion efficiency was much lower, reaching about 3% by 48 days post-stroke. Neuronal transformation occurred in cells close to the lesion core. The authors suggest that the low conversion efficiency may be the result of the cortical microenvironment [177].

It is known that some TFs may be subjected to posttranslational modifications limiting their activity, for example, phosphorylation. To overcome this, Ghazale et al. made a mutant version of Ascl1 with a few serines in phospho-acceptor sites substituted to alanines (ASCL1SA6). AAV8 vectors expressing either WT or mutant ASCL1 were injected into the mouse motor cortex. Glia-to-neuron conversion measured at 14 dpi was higher for ASCL1SA6: 80%, compared to 58% for ASCL1. Immunohistochemistry for MAP2, a dendritic marker, showed that compared to *Ascl1*-expressing cells, *Ascl1SA6*-expressing cells have more elaborate dendritic arbors and their morphology is closer to that of mature neurons. It should be noted that in this study, a control virus expressing no TFs also labeled NeuN+ neurons (14% of all labeled cells), which could mean leaky expression from the chosen promoter in preexisting neurons [350]. A more recent study that used ASCL1SA6 showed that the mutant factor exhibited increased neurogenic activity (compared to that of ASCL1) in the glia of the early postnatal (P5) mouse cortex when expressed from a retroviral vector. An additional increase in neuronal conversion was achieved by the coexpression of Bcl-2, which prevented cell death by ferroptosis. Immunohistochemistry at 28 dpi showed that among the cells that were double-transduced with *Ascl1SA6* and *Bcl2*, ~25% were GABA+ and ~20% were PV+ [336]. The same research group later used the coexpression of *Ascl1SA6* with *Dlx2* to increase the efficiency of astrocyte conversion into GABAergic interneurons. At 12 dpi, 37% of labeled infected cells were GABA+ [351].

### 4.4. Overexpression of Transcription Factor Neuronal Differentiation 1 (NeuroD1)

NeuroD1 is a pioneer TF able to activate silent genes to induce the neurogenic program [352]. It is involved in glutamatergic neuron differentiation both in the developing and in the adult brain [353].

To test the transdifferentiation potential of astrocytes in an intact brain, Brulet et al. used a systemic administration of the AAV9 vector known to be able to cross the BBB and predominantly target astrocytes in adult mice [354]. The authors confirmed that an i.v. injection of this virus expressing GFP from a universal promoter causes mostly astrocytes to be infected; the fractions of NeuN+ among GFP+ cells were 0.79% in the cortex and 1.07% in the striatum. The expression of *Neurod1* from the virus did not transform astrocytes into neurons in the cortex; in the striatum, a small number of iNs was registered. The authors suggest that astrocytes in an intact brain may not be well-responsive to NeuroD1-induced transformation; it is likely that reactive astrocytes located at brain lesions would be reprogrammed more effectively [28]. Another study addressing the differences of neuronal transformation between reactive and resting astroglia used the inducible and lineage-traceable *Neurod1* knock-in mouse strain. Tamoxifen-dependent Cre recombinase was expressed from the astrocyte-specific *Aldh1l1* gene promoter, and the NeuroD1-P2A-EGFP construct was put under the universal CAG promoter, but a floxed STOP sequence prevented its expression. Upon tamoxifen administration, Cre in astrocytes removed the STOP sequence, switching on a stable expression of NeuroD1-P2A-EGFP. Using this strain, the authors demonstrated iNs generated from astrocytes (NeuN+, GFP+ cells) in the lesion core of either SCI, focal stroke, or stab wound brain injury, but not in peri-injury distal regions in all lesion models. Tamoxifen-induced *Neurod1* expression in astrocytes was only effective for transformation if it started at the early post-injury phase (5–8 days); delayed *Neurod1* expression start at 14–16 days after the SCI failed to induce the transformation. These data confirm that reactive proliferating astrocytes are more susceptible to NeuroD-induced transformation compared to resting astroglia in undamaged brain regions [355].

In some works, the subtypes of NeuroD1-iNs were identified. For example, Guo et al. used *Neurod1* expression from a retroviral vector to transform astrocytes and OPCs into neurons in the mouse cortex after a stab wound. The GFAP promoter was used in the vector designed for astrocytes, and the NG2 promoter was used for expression in OPCs. The overexpression of NeuroD1 caused astrocyte conversion mostly into glutamatergic neurons (VGlut1+), while OPCs produced both VGlut1+ and GAD67+ cells (VGlut1+ still comprised the majority of iNs) [318]. Puls et al. used an AAV vector expressing *Neurod1* in a model of SCI; the virus was injected directly after the injury. Infected cells showed a progressive increase in the percentage of iNs (NeuN^+^/GFAP^−^), reaching ~95% at 8 wpi. Most iNs in the dorsal horn were positive for TLX3, a marker of spinal cord glutamatergic neurons [356]. Liu et al. expressed *Neurod1* from an ssAAV vector with GFAP promoter (GFAP::NeuroD1-P2A-GFP) in the cortex, striatum and corpus callosum of intact mouse brain. NeuroD1-induced astrocyte-to-neuron transformation was not effective in the white matter of corpus callosum; very few GFP+ cells found there were negative for most tested neuronal subtype markers. At 5 wpi, about 27% of GFP+ cells in both the cortex and the striatum were immunopositive for GABA. In the cortex, 11% of the labeled cells were PV+. Other markers identified in some GFP+ cells in the cortex were TBR1 (a marker of cortical neurons), CTIP2 (a marker for deep-layer neurons and striatal projection neurons), and SATB2 (a marker for superficial layer neurons). In the striatum, some GFP+ iNs were positive for GABA, CTIP2, or TBR1, but iNs expressing PV or SATB2 were rare. These data show that *Neurod1* expression from the GFAP promoter may cause the formation of both excitatory and inhibitory neurons, and that the microenvironment in different brain regions affects the proportions of iN subtypes [357].

Chen et al. used a Cre-FLEX system where the first AAV vector expressed Cre from the GFAP promoter, and the second vector was FLEX-CAG::NeuroD1-P2A-GFP. They demonstrated that after a chemically induced stroke in the mouse motor cortex, iNs replenished about one-third of the lost neurons. Immunohistochemistry showed that iNs mostly expressed cortical pyramidal neuron markers, while about 10% of these cells were GABA+, and about 10% were PV+. It was confirmed that the iNs send out long-range axon projections to the thalamus and partially restored stroke-induced motor deficits in mice [246]. A similar Cre-FLEX system for *Neurod1* expression was used by Zhang et al. who studied the conversion of reactive astrocytes into neurons in an established glial scar in the stab-injured mouse cortex. They found that reactive astrocytes were efficiently converted into neurons both before and after glial scar formation (virus injection as late as 21 days after the injury was effective). iNs were highly functional, capable of firing APs and establishing synaptic connections with other neurons. Unexpectedly, the expression of *Neurod1* in reactive astrocytes resulted in a significant reduction in A1 astrocytes, and a decrease in reactive microglia and neuroinflammation [358]. NeuroD1 expression with a Cre-FLEX system was also studied in non-human primates. Ge et al. used GFAP:Cre and FLEX-CAG::NeuroD1-P2A-mCherry vectors with a cortical stroke model in monkeys. They achieved very high neuronal conversion efficiency (94.4%) with this combination of viruses in the intact monkey cortex. Then, they modeled a stroke by cortical injections of endothelin-1 (ET1) and showed that the virus encoding NeuroD1 (administered 10–30 days after the stroke) alleviated tissue damage and microgliosis and increased the number of NeuN+ cells. The number of PV+ cells near the virus injection site was also increased. While the majority of NeuroD1-converted neurons were PV-, a small number of PV+ neurons in the NeuroD1-infected areas expressed the mCherry marker from the virus. The authors conclude that NeuroD1-induced neuronal transformation also somehow protected preexisting PV+ GABAergic interneurons from the stroke-related damage, which may have important clinical implications [359]. Other experiments with NeuroD1 overexpression after stroke also showed therapeutic effects beyond the formation of new neurons. Jiang et al. injected an LV vector expressing *Neurod1* into the murine cortex 7 days after the focal stroke induction. This treatment not only generated iNs, but also increased synaptic transmission in the peri-infarct region (measured as the amplitude of the first excitatory postsynaptic potential (EPSP) after a stimulation) as compared to the group treated with a control virus. The authors observed abnormally increased pair-pulse facilitation (the ratio of the second to first EPSP when paired electric stimuli are applied) in the mouse cortex after stroke; a similar phenomenon in stroke patients is considered a type of pathological plasticity. Notably, the NeuroD1-expressing virus prevented increased pair-pulse facilitation after stroke, suggesting that this kind of gene therapy may restore normal synaptic plasticity [27]. This result is important to consider for epilepsy research as well, because the changes in synaptic plasticity after an ischemic lesion may be one of the reasons for epileptiform discharges developing later [360]. One specific type of ischemic pathology known to contribute to epileptogenesis is neonatal hypoxic-ischemic brain injury (HIBI). Kim et al. used a mouse model of this pathology and 3 days after the injury delivered NeuroD1 to the injury site using AAV vectors and a Cre-FLEX system with Cre expressed under the GFAP promoter. High astrocyte-to-neuron conversion was achieved: more than 80% by 30 dpi, estimated by the loss of GFAP expression and the gain of NeuN expression. About a half of the iNs were identified as GABAergic (GAD67+). The viral treatment after the injury not only generated new neurons, but also protected preexisting neurons and decreased the numbers of A1 reactive astrocytes and reactive microglia. NeuroD1-treated HIBI mice exhibited improved motor functions [326].

Another cell type that may be considered for NeuroD1-induced neuronal transformation is microglia. Matsuda et al. induced *Neurod1* overexpression in mouse microglia in vitro and in vivo using lentiviral vectors with *CD68* gene promoter (specific for immune cells including microglia). The experiments confirmed the transformation of transduced cells into neurons and their functional maturation [330]. However, Rao et al. showed that NeuroD1 actually causes the apoptosis of microglia and does not induce microglia-to-neuron conversion in mice. Even with anti-apoptosis rescue, microglia-to-neuron conversion was not observed. A stringent lineage tracing showed that no labeled neurons were derived from microglia [331]. These two research groups also disagreed about whether their conflicting results may be explained by the difference in NeuroD1 expression level [332,361]. Finally, the group that claimed successful microglia-to-neuron conversion by NeuroD1 used Hexb-Cre^ERT2^::Stop-YFP mice that selectively express YFP in microglial cells to confirm the lineage of the transformed neurons. In the same study they showed that a cocktail of drugs, namely, VPA, forskolin, and Y-27632 (Rho-associated protein kinase inhibitor), increases the efficiency of NeuroD1-induced microglia-to-neuron conversion [333]. They continued studying the potential of microglia for transdifferentiation in a damaged brain in the tMCAO model of stroke in mice. It was demonstrated that after the stroke, microglia/macrophages accumulate at the ischemic core in the striatum. An overexpression of *Neurod1* by an LV vector with CD68 promoter in the ischemic core increased the number of Tubb3+ and MAP2+ cells. Moreover, NeuroD1-transduced cells stained positive for NeuN and DARPP32. In patch-clamp experiments, these cells showed spontaneous AP firing following depolarization and spontaneous excitatory and inhibitory postsynaptic currents. *Neurod1*-expressing virus treatment after the stroke rescued neurological dysfunctions in mice (asymmetric body movement in behavioral tests) [362].

There is also some evidence suggesting that NeuroD1-induced neuronal transformation may not actually be effective. Wang et al. tried to reproduce some previously reported results with more stringent lineage-mapping strategies. They obtained reporter-labeled neurons after the AAV-mediated co-expression of *Neurod1* and a reporter in the mouse brain. However, they could not trace the reporter-labeled neurons retrospectively to resting or reactive astrocytes. They conclude that the labeled neurons are actually pre-existing neurons that were infected by the virus, even though the AAV5 serotype used for the infection targets astrocytes with high specificity and the hGFAP astrocyte-specific promoter was used. One proposed mechanism of this is the upregulation of the hGFAP promoter activity by NeuroD1 itself [363]. Possible caveats of this research pointed out later by Zheng et al. are the high dose of the virus and the low expression level of NeuroD1 [364]. Chen et al. reported a different problem with the NeuroD1-based transdifferentiation strategy. They showed that iNs are not physiologically functional; their morphology, electrophysiological properties and ability to form synaptic connections were not resembling mature neurons even at 16 weeks post-NeuroD1 induction. This result contradicts many previous reports, and the authors suggest that NeuroD1 expression levels and timing may be the factors affecting the functionality of the iNs [355].

### 4.5. Overexpression of SRY-Box Transcription Factor 2 (SOX2)

SOX2 is an important transcriptional regulator in stem cells. SOX2 is active in NSCs and regulates their self-renewal, suppressing premature neuronal differentiation [365]. The differentiation of neurons and glia normally involves SOX2 downregulation. However, some types of fully differentiated neurons and glia retain high SOX2 expression, which is important for their function [366].

Niu et al. showed that the overexpression of *Sox2* alone is sufficient to reprogram resident astrocytes of the adult mouse brain into proliferating DCX+ cells they termed “induced adult neuroblasts (iANBs)”. However, to differentiate into electrophysiologically mature neurons, these iANBs then required additional factors (BDNF and Nog, or VPA) [311]. In a following study by this group, this method of astrocyte reprogramming was studied in more detail. It was established that the iANB stage is preceded by the ASCL1+ neural progenitor stage. The fate of iANBs differentiated into neurons by neurotrophic factors or VPA was also delineated; in experiments in vivo where iANBs were generated by *Sox2* overexpression in mouse striatum, it was found that they predominantly give rise to calretinin+ interneurons [367]. Later, the same group screened multiple transcription factors and signaling molecules to improve the production of iANBs in the adult spinal cord. It was found that the p53 pathway is a critical checkpoint that controls the cell cycle exit of iANBs, and the downregulation of this pathway leads to a significant increase in iANB production [368].

Tai et al. showed that after SCI, OPCs initiate but do not complete a neurogenic program (see Section 3.2.2). It was shown that *Sox2* is upregulated during this process; in Pdgfra-CreER^TM^;Sox2^f/f^;Rosa-tdT mice with conditional *Sox2* knock-out in OPCs, the number of injury-induced DCX+ cells was reduced by 76.5%. The overexpression of *Sox2* in the spinal cord by an LV vector with the NG2 promoter caused a more than 15-fold increase in DCX+ cells around the injection area at 4 wpi. The further analysis of these cells showed that many of them pass through an ASCL1+ proliferating progenitor stage and become NeuN+ neurons. The number of neurons induced by SOX2 was greatly increased by the coexpression of neurotrophic factors: BDNF and Nog, or p75-2 (a mutant form of NT3). Among these iNs, an approximately equal ratio of excitatory (VGlut2+) and inhibitory (GAD65+ or VGAT+) neurons was shown; some inhibitory neurons coexpressed GlyT2, a marker for glycinergic neurons. Synaptic tracing confirmed that iNs form connections with propriospinal neurons, neurons in dorsal root ganglia and the brain stem. When a virus encoding SOX2 and p75-2 was injected 1 week after the injury, glial scar volume and surface area (determined by GFAP staining, 26 weeks post-injury) were significantly reduced. Mice treated with the virus showed functional recovery in the grid-walking test [294]. The retroviral expression of either *Ascl1*+*Sox2* combination or *Sox2* alone caused a conversion of OPCs into DCX+ immature neurons in the mouse cortex following stab wound injury. However, the expression of *Sox2* from an LV vector in an intact brain did not induce such conversion. The conversion was also not observed in the damaged brain when *Ascl1* was expressed alone [349].

Importantly, endogenous *Sox2* upregulation in astrocytes contributes to their activation, and this worsens the outcome of injury. It was shown that the astrocyte-specific deletion of *Sox2* in mice results in much reduced lesion size and significantly improves behavioral recovery after TBI [369]. Thus, it should be considered that the artificial overexpression of *Sox2* in glia may have both beneficial and detrimental effects.

### 4.6. Overexpression of Transcription Factor Neurogenin 2 (Ngn2)

The neurogenin family, comprising Ngn1, Ngn2, and Ngn3, is a class of proneural transcription factors. Ngns are expressed throughout the developing nervous system. These factors have major roles in the commitment of progenitors to neurons by inhibiting glial fate, inducing a cascade of pan-neuronal genes, and promoting cell cycle exit, neuronal migration, and the expression of neuron subtype-specific genes. Ngn2 is transiently expressed in NPCs and is downregulated during the final differentiation stages. Ngn2 is able to rapidly induce neuronal differentiation from different starting cell types in experiments (reviewed in [370]). An alternative name for this factor is Neurog2.

Zhang et al. considered Ngn2 as one of the possible TFs whose overexpression may be sufficient for neuronal transformation without additional factors. They expressed *NGN2* in human embryonic stem cells (ESCs) or induced pluripotent stem cells (iPSCs) in vitro using an LV vector, and showed that in 2 weeks, functionally active excitatory neurons are formed. iNs expressed VGlut2 and markers characteristic of cortical layer 2/3 neurons (BRN2, CUX1, and FOXG1) [371]. However, further research showed that the Ngn2-induced reprogramming of iPSCs may produce diverse neuron types. Lin et al. observed three different iN populations, two of them expressing genes associated with PNS neurons [322] (see Section 4.1). Galiakberova et al. obtained iNs expressing transcripts characteristic for glutamatergic, cholinergic, or peripheral sensory neurons [372].

For astrocytes as the starting cell type, Hu et al. demonstrated that Ngn2-induced neuronal transformation is less efficient if astrocytes are taken from the adult brain, as compared to early postnatal astrocytes; moreover, the CNS region from which astrocytes were taken was also an important factor [327]. An et al. used a complex approach to overexpress *Ngn2* in astrocytes in a mouse model of SCI. They developed a combination of split-Cre under two separate promoters specific for reactive astrocytes and the Cre/DIO-dependent inversion of *Ngn2* under universal EF1α promoter (TRANsCre-DIONE). In a preprint, the authors claim 87% efficiency and 96% specificity of transformation of scar-forming reactive astrocytes into neurons by this approach [373]. However, we could not find any follow-up study where this method was used.

Bazarek et al. used a retroviral vector to express *Ngn2* in OPCs in the rat cortex. This virus infects only dividing cells, thus providing selective OPC targeting in the intact adult brain. The authors demonstrated a high efficiency of the virus for the transformation of OPCs into NeuN+ neurons. The type of iNs was not defined [329].

Ngn2 was also used in neuronal transformation experiments where the starting cell type was microglial lineage cells. In a recent work by Jin et al., cultured human microglial populations derived from ESCs were shown to transition toward a neuronal identity following the activation of *NGN2*. The acquisition of neuronal morphology, expression of neuronal markers, and functional excitability characterized by AP firing were demonstrated in the iNs. Moreover, in experiments where the authors transplanted human ESC-derived primitive macrophage progenitors with Dox-inducible *NGN2* into the brains of newborn mice, they provided proof-of-concept evidence that xenografted microglia can be converted into neurons in the brain [334].

Neuronal reprogramming by Ngn2 in vivo was shown to meet some obstacles, including the significant influence of microenvironment on the efficacy of reprogramming [314] and inflammatory conditions hindering the reprogramming in a model of intracerebral hemorrhage [374]. To optimize in vivo transdifferentiation by this TF, additional factors may be used. For example, Mattugini et al. demonstrated that the overexpression of *Ngn2* together with the nuclear receptor-related 1 protein (*Nurr1*) gene improved the efficiency of reactive astrocyte reprogramming in an injured mouse cortex. The role of Nurr1 in this process is supposedly linked to its function of inhibiting inflammatory signals in microglia and astrocytes, since neuronal reprogramming is vulnerable to ROS [375].

### 4.7. Overexpression of Transcription Factor Distal-Less Homeobox 2 (DLX2)

DLX2 is a TF important for forebrain GABAergic neuron differentiation [376], so it was used in a number of studies aimed to generate this type of neurons, alone or in combination with other neurogenic factors.

In the study by Liu et al., it was observed that astrocytes may be converted into DARPP32+, CTIP2+ medium spiny neurons under the influence of DLX2 (expressed from an AAV vector injected in the striatum) in the intact mouse brain. The transformation was observed in both gray matter (striatum) and in the neighboring white matter (corpus callosum). However, in white matter, only partial conversion of the astrocytes to an intermediate state happened; this was accompanied by enhanced inflammation. The co-expression of *Neurod1* together with *Dlx2* reduced this undesired side effect [377]. Cooper et al. showed that the co-expression of *Dlx2* causes iNs generated by *Ascl1SA6* expression in astrocytes to become more similar to the GABAergic interneuron phenotype [337] (see Section 4.1). Zhang et al. used DLX2 to transform astrocytes into GABAergic neurons in the mouse striatum. These authors did not combine DLX2 with any additional TFs and chose an LV vector. Aldh1l1-CreER^T2^;R26R-tdTomato mice were used to trace astrocyte fate. It was demonstrated that the expression of *Dlx2* alone is sufficient for a high conversion rate; among tdT+/NeuN+ cells, more than 80% were positive for GABA. However, none or very few cells positive for markers of different GABAergic neuron subtypes: PV, SST, calbindin or DARPP32, were observed. A small fraction of tdT+/NeuN+ cells expressed calretinin. In separate experiments, DLX1 and DLX5, other TFs of the DLX family that are upregulated by *Dlx2* overexpression in astrocytes, were tested for their ability to induce neuronal transformation. LV vectors expressing *Dlx1* or *Dlx5* were able to transform astrocytes into DCX+ immature neurons; DLX5 was more efficient for reprogramming [338].

Wu et al. developed a method of increasing the fraction of GABAergic neurons among iNs transformed from sriatal astrocytes in two mouse models of HD. The authors combined *Neurod1* and *Dlx2* expressed from AAV2/5 vectors; a selective expression in astrocytes was achieved by a Cre-FLEX system with the GFAP::Cre virus. At 30 dpi more than 80% of transduced cells stained positive for GABAergic neuron markers, GAD67 or GABA. Markers of GABAergic neuron subtypes were also estimated: 55.7% of transduced cells expressed DARPP32, 9.6% were PV+, and the fractions of neurons expressing somatostatin, NPY, or calretinin were lower [378]. The same group later used a combination of *Neurod1* and *Dlx2* expressed from AAV vectors in a model of SCI; while transformation by NeuroD1 alone mostly induced glutamatergic neurons, the addition of DLX2 led to a 5-fold increase in the number of PAX2+ GABAergic spinal cord neurons among iNs [356].

DLX2 was also successfully used for OPC-to-neuron conversion. A subpopulation of OPCs in the neocortex arises from progenitor cells in the embryonic ganglionic eminences that also produce inhibitory neurons, so the lineage distance between these cell types is small. Boshans et al. showed that the overexpression of *Dlx2* in cultured OPCs from early postnatal mouse neocortex induces these cells to acquire an interneuron phenotype. They started expressing markers of interneurons, including synaptic proteins. Additionally, some iNs were able to generate APs [379].

Another cell type used for DLX2-induced neuronal differentiation is NSCs. Tang et al. worked with a stroke model with ET1 injected into the mouse brainstem. The brainstem mostly contains GABAergic neurons, and immunostaining confirmed that the loss of GABAergic neurons in this stroke model is more prominent than the loss of other neuron types. The absence of endogenous NSCs within the infarct area in this model was confirmed by staining for Ki67 and nestin. Embryonic donor NSCs used in this study underwent different manipulations in vitro before transplantation. NSCs transfected with a control LV vector showed the lowest survival rate after the transplantation. A vector encoding BDNF increased their survival, but later most of these cells differentiated into ChAT+ cholinergic neurons. Finally, when *Dlx2* was co-expressed with *Bdnf* in the same virus, the majority of grafted NSCs differentiated into GABAergic neurons (labeled by a GAD65+67 antibody). In animals who received the treatment, induced GABAergic neurons reduced infarct volume and improved motor and sensory recovery. Transsynaptic virus tracking, immunohistochemistry and patch clamp experiments confirmed the functional integration of the iNs into the existing neural circuits [207].

### 4.8. Downregulation of Notch1 Signaling

Notch is a highly conserved signaling pathway involved in cell–cell interactions. It is necessary for multiple processes in embryonal development and tissue homeostasis. In mammals, there are four Notch receptors (Notch1–Notch4) interacting with Notch ligands on the surface of adjacent cells [380,381,382]. In the mammalian CNS, Notch signaling maintains the undifferentiated state of NSCs [309], but at the same time enhances astrogliogenesis by NSCs [310]. Notch1 and presenilin 1 (the catalytic subunit for γ-secretase that activates Notch1 by cleavage) are involved in the generation and proliferation of neurospheres from reactive astrocytes isolated from cortical peri-infarct tissues [262].

Tan et al. induced neuronal reprogramming in primary astrocyte cultures by either ASCL1 or Ngn2 and performed Western blotting for key proteins of different signaling pathways. Both reprogramming methods decreased the expression of Notch1-ICD (Notch1 intracellular domain). The authors then tested whether the downregulation of Notch1 is sufficient for the conversion. A knockdown of *Notch1* by shRNA or a pharmacological inhibition of the Notch1 signaling pathway by DAPT (γ-secretase inhibitor) in cultured astrocytes increased the expression of genes encoding neurogenic TFs: *Neurod1*, *Neurod2*, *Pax6*, *Lmx1a* and *Lhx6*. Astrocytes infected with an LV expressing the Notch1 shRNA adopted a typical neuronal morphology and became positive for the pan-neuronal marker Tubb3 at 12 dpi. DAPT treatment had similar effects. In experiments in vivo, the authors first showed that in the adult mouse spinal cord, *Notch1* expression in astrocytes increases after SCI. Then, they injected LV expressing Notch1 shRNA into the spinal cord immediately after the injury. At 7 dpi, DCX+ cells co-labeled with GFP expressed from the virus were detected around the injection site. These cells incorporated BrdU, suggesting their origin from proliferating astrocytes. I.p. injections of DAPT for 8 days starting at the day of injury had similar results. For DAPT, the astrocytic origin of newly generated neurons was confirmed by viral tracing and genetic lineage tracing. The cellular fate of DAPT-induced neurons was further evaluated by immunostaining. At 4 dpi, BrdU-incorporated MAP2+ or NeuN+ cells were detected. DAPT-iNs (BrdU+/Tubb3+) were positive for GABA and GAD65/67, but not ChAT or VGlut1. The authors emphasize that DAPT, a small-molecule drug that may be preferable to gene therapy in clinical applications, had effects similar to the viral vector treatment [32]. Hu et al. also screened different signaling pathway proteins in astrocytes being subjected to neuronal conversion in vitro. They used LV vectors expressing *Ngn2* or *Ascl1* and showed that murine astrocytes from different brain regions and from animals of different ages have different susceptibilities to these conversion factors (see Section 6.3). In search of the mechanisms behind this difference, they found a correlation between a higher *Notch1* expression and a lower transdifferentiation efficiency. Some other genes involved in Notch1 signaling also were differentially expressed between astrocyte groups, consistent with the idea that the Notch1 pathway is downregulated in astrocyte populations showing more efficient neuronal conversion. To confirm the causal role of Notch1 downregulation in conversion rate difference, the authors used DAPT treatment before the administration of LV vectors to the cultures. The inhibition of Notch1 pathway with DAPT resulted in an increase in the Ngn2- or ASCL1-mediated neuronal reprogramming efficiency of astrocytes from all studied regions. DAPT treatment did not affect the maturation and cell subtype of the iNs. An alternative method to inhibit Notch1 signaling in astrocytes, a knockdown of *Notch1* by a shRNA, also increased the yield of iNs, similarly to DAPT treatment [327].

As was mentioned in Section 3.1.2, Magnusson et al. found that stroke may start a neurogenic program in astrocytes. Further experiments showed that attenuated Notch1 signaling is necessary for that program. Moreover, the artificial blockade of Notch1 signaling triggered this program even in the absence of stroke. The astrocyte-specific deletion of *Rbpj* (encoding an obligatory TF for canonical Notch signaling) with the CreER method triggered neuroblast production by astrocytes in the murine striatum and medial cortex. This continued for a long time; even 34 weeks after tamoxifen administration, ASCL1+ astrocytes and DCX+ cells were present in the striatum. A fraction of cells positive for the Cre recombination marker YFP developed elaborate processes and neuronal morphology, expressed NeuN and nNOS (a marker for GABAergic medium-sized striatal interneurons), and some of these cells had formed synaptic connections [269]. The same research group later used scRNA-Seq to characterize Notch-depleted astrocytes undergoing neurogenesis in vivo. It was found that the neurogenic program of striatal astrocytes is very similar to that of neural stem cells in the SVZ, but not in the DG. In the non-neurogenic cortex, Notch-depleted astrocytes also initiated neurogenesis, but stalled before entering transit-amplifying divisions. Some striatal astrocytes also stalled, and it was found that EGF may stimulate these cells to resume neurogenesis. Notably, in this study, proliferating astrocytes never showed signs of reactive gliosis [160].

Another study by this group using scRNA-Seq clarified that after stab wound injury in mice with Notch-depleted astrocytes, astrocyte neurogenesis and reactive gliosis happen independently; these processes are induced in parallel by different subsets of astrocytes [341] (see also Section 4.1). Since this was observed specifically in the mouse strain where all the astrocytes are Notch-depleted, it is possible that these results may not be readily transferred to WT astrocytes, because normally astrocyte activation includes Notch1 upregulation [32], and in the Notch-depleted astrocytes the mechanisms of activation may be seriously altered. Indeed, scRNA-Seq showed considerable molecular differences between WT and *Rbpj*-depleted astrocytes, which clustered separately from each other; moreover, mutant astrocytes from injured and healthy cortex showed less differences from each other than from WT astrocytes. No neurogenesis by astrocytes as a response to injury in WT mice was registered in this study [341].

scRNA-Seq data from the studies [160,341] were later reanalyzed by Nato et al. who studied the properties of lesion-born striatal neurons (see Section 3.1.2). It was shown that *Rbpj* mutation in both striatal and cortical astrocytes causes them to produce neurons of the same LGE_MEIS2/PAX6 interneuron class (mostly found in the embryonic CNS), even though in the case of the cortex most cells mapped to more immature, proliferating or early post-mitotic classes [272].

### 4.9. Downregulation of Polypyrimidine Tract Binding Protein 1 (PTBP1) Expression

During neuronal development in mammals, the alternative splicing of multiple exons is reprogrammed by a switch in expression between two polypyrimidine tract-binding proteins, PTBP1 (also known as PTB) and PTBP2 (neural PTB, or nPTB). PTBP1 expression is restricted to nonneuronal lineages while PTBP2 is found only in post-mitotic neurons. PTBP1 downregulates PTBP2 expression on transcriptional and post-transcriptional levels. In the cells undergoing neuronal differentiation, a neuron-specific microRNA miR-124 reduces PTBP1 translation, which allows the high expression of PTBP2 protein, eventually causing the transition to neuron-specific splicing patterns [383,384].

Xue et al. performed *PTBP1* knockdown in vitro in various cell cultures using shRNA. They reported a decrease in the growth rate and the emergence of MAP2+ and Tubb3+ cells even in HeLa, an immortalized cell line with a severely rearranged genome. Cultures of primary mouse embryonic fibroblasts and N2A (mouse neuroblastoma line), when subjected to *Ptbp1* downregulation, started expressing NeuN and VGlut1. These cells also developed some neuron-like electrophysiological properties, as was shown with patch-clamp recording. The authors also studied the mechanisms by which *PTBP1* downregulation causes these changes, and revealed multiple ways in which PTBP1 regulates miRNA–mRNA interactions [321].

In vivo, one of the pathologies wherein glial *Ptbp1* downregulation was tested is stroke. Yuan et al. expressed an shRNA targeting *Ptbp1* transcript by an AAV vector with the GFAP promoter and the GFP reporter, injected in the mouse cortex 10 days after stroke induction by ET1. At 60 dpi, astrocyte-to-neuron conversion was observed: almost 80% of GFP-labeled cells expressed NeuN, while in the group infected with a control virus most GFP+ cells were colabeled with GFAP. Some iNs were positive for VGlut1 or CTIP2 (marker of neurons in deep cortical layers). The functional rescue of stroke consequences in shPTB-treated mice was confirmed by gait analysis [385]. Fukui et al. used a different model of stroke, tMCAO, and a CRISPR-CasRx-mediated *Ptbp1* knockdown. An AAV vector with the GFAP promoter expressing CasRx nuclease and sgRNA targeting *Ptbp1* was injected i.v. 3 days after stroke. At 56 days after stroke, hippocampal neurogenesis was robustly increased and showed significantly recovered hippocampal recognition memory (novel object recognition test). The authors suggest that the observed results may be explained by the virus stimulating neurogenesis by both NSCs and hippocampal astrocytes [30].

A number of studies addressed *PTBP1* downregulation in glia in models of PD. Qian et al. showed an efficient conversion of mouse and human astrocytes in vitro into functional neurons by depleting PTBP1 with an LV vector expressing shRNA against *PTBP1*. The same shRNA in an AAV vector injected into mouse substantia nigra converted astrocytes into dopaminergic neurons in situ in a PD model. The authors demonstrated the reconstruction of the nigrostriatal circuit and the rescue of motor deficits [386]. However, a different research group could not reproduce the results reported by Qian et al. using a conditional knock-out of *Ptbp1* in a mouse line with genetically traced astrocytes [387]. Zhou et al. employed the CasRx method to target *Ptbp1* and showed its efficiency in two mouse models of neuropathologies: PD and retinal injury. In the PD model, sgRNAs targeting *Ptbp1* were expressed under the GFAP promoter from an AAV virus injected into the striatum 3 weeks after PD induction, together with AAV-GFAP-mCherry for viral lineage tracing. Induced dopaminergic neurons (mCherry+, TH+ cells) were observed 1–3 months post-infection (mpi). The differentiation of some iNs towards a mature dopaminergic phenotype was confirmed by staining for midbrain dopaminergic neuron markers DAT, DDC and FOXA2. The expression of area-specific dopaminergic neuron markers was also found. Importantly, in this study the ability of iNs to release dopamine was confirmed in functional tests with a fluorescent dopamine derivative. Experiments with striatal slices showed that iNs were capable of generating APs and received functional synaptic inputs; behavioral experiments showed that iNs alleviated motor dysfunctions [388].

Liu et al. used a highly astrocyte-specific AAV vector to express shRNA-downregulating *Ptbp1* in astrocytes in a mouse model of TBI with glial scar. They report successful astrocyte-to-neuron conversion; however, no data related to the scar reduction are present [29]. Yang et al. also studied a condition with a glial scar. They used an shRNA expressed from the GFAP promoter by AAV to knock down *Ptbp1* expression in reactive astrocytes. Another treatment was an antisense oligonucleotide targeting *Ptbp1* transcript (PTB-ASO). The treatments were injected into the mouse spinal cord near the lesion site 7 days after SCI. Both treatments produced iNs, as was shown at 12 wpi by staining for NeuN, MAP2 and ChAT, a motor neuron marker. Both treatments caused motor function recovery 5–12 weeks after the SCI. It was also shown that PTB-ASO prevents the apoptosis of injured spinal cord cells (estimated by expression levels of caspase-3 and caspase-9) and reduces the density of the glial scar (estimated by GFAP staining). The authors emphasize that the scar density was decreased without disrupting its overall structure, which is preferable to an exhaustive glial scar ablation [389].

Maimon et al. worked with aged mice. They used a PTB-ASO, injected i.c.v. to GFAP-Cre^ERT2^, CAG-lox-stop-lox-tdTomato mice (stably expressing tdT in cells with the GFAP promoter being active at the time of Cre induction), aged 3 months or 1 year. In young mice, a tenfold increase in NeuN+/tdT+ cells was shown in the first and fourth layers of the cortex and in the CA1 region of the hippocampus at 2 mpi. In the DG, a striking 37.5-fold increase of NeuN+/tdT+ cells was observed. iNs comprised 7.8% of all neurons of the granule cell layer in the young DG. In the DG of aged mice, new NeuN+/tdT+ neurons comprised 4.5% of all neurons at 2 mpi. PTB-ASO treatment appeared to trigger the typical neuronal maturation process. Ki67 was elevated in the DG of both young and aged PTB-ASO-treated mice, indicating increased cell proliferation. Gradual changes in marker expression (DCX, NeuN, MAP2) and cell morphology in tdT+ cells corresponded to canonical neuronal development; electrophysiological recordings from tdT+ neurons in the DG were indistinguishable from preexisting tdT- neurons. A very important finding in this study is that the main GFAP+ cell population generating new hippocampal neurons after transient *Ptbp1* downregulation is not astrocytes, but NSCs, based on their morphology and position. Many transitioning tdT+ cells were GFAP+, but GFAP was not located in bundles in the cell body, like in astrocytes, but only in dendrites. PTB-ASO-treated aged mice showed an improvement in hippocampal function in the Barnes maze test and novel object recognition test [31].

We must note that in a few cases, more recent studies using stringent lineage-tracing strategies did not reproduce the *Ptbp1*-downregulation-based glia-to-neuron conversion demonstrated earlier in the brain or retina. Some examples of these studies challenging previous reports are [363,387,390,391,392]. A possible explanation (mentioned in some of the papers cited here) may be the leaky neuronal expression of AAV vectors with the GFAP promoter (see Section 6.4). It is also possible that such vectors stimulated neurogenesis by *Gfap*-expressing NSCs, as described in [31], which could have been misinterpreted as astrocyte transformation.

### 4.10. Neuron-Specific microRNA miR-124 Used in Neuronal Conversion

miR-124 is one of the most abundant microRNAs in the brain. It downregulates the translation of multiple mRNA targets, including those encoding TFs. miR-124 is an important regulator of neurogenesis that orchestrates TF networks involved in neuroprotection and neuronal subtype differentiation [393]. miR-124 also drives metabolic changes at the initiating stage of human neurogenesis [394]. miR-124 and PTBP1 form a genetic regulatory circuit, inhibiting each other [383,395].

Papadimitriou et al. tested whether miR-124 alone is sufficient to drive direct astrocyte-to-neuron reprogramming. They found that while miR-124 is able to induce the transformation alone, the addition of the neurogenic compound ISX9 greatly enhances miR-124-induced reprogramming efficiency and promotes iN maturation in vitro. Without ISX9, miR-124 overexpression produced immature neurons, positive for precursor cell markers ASCL1 and TBR2. Interestingly, miR-124 reduced NeuroD1 mRNA levels, which likely contributed to the low differentiation capacity of miR-124-iNs. With the addition of ISX9, iNs matured, exhibited neuronal morphology and stained positive for Tubb3, MAP2 and synapsin I. Most of these iN were identified as glutamatergic (VGlut1+), with a small fraction being GABA+. The authors identified ZFP36L1, a protein implicated in mRNA decay, as a novel direct target of miR-124. ZFP36L1 downregulation happened very early during reprogramming and may play a key role in it, since many ZFP36L1 mRNA targets encode neuron-specific proteins. In experiments in vivo, LV or AAV9 vectors expressing miR-124 were injected into injured mouse cortex 4 days post-injury. At 3 wpi, the fraction of NeuN+ neurons among marker-labeled infected cells was much higher than at 7 dpi for both viruses. However, the authors note that the used viruses may also infect resident neurons. They provided indirect confirmations that at least some labeled neurons were induced by glial reprogramming: BrdU incorporation showed that some neurons labeled with the AAV9 infection marker originated from proliferating cells, and some marker-labeled cells expressed DCX. The addition of ISX9 aided reprogramming in vivo by improving iN survival, but in contrast to the in vitro observations, the miR-124/ISX9 synergistic action was not sufficient to drive the full maturation of iNs [340].

Mseis-Jackson et al. proposed an original use for miR-124 in refining *Neurod1*-induced neuronal transdifferentiation. They suggested that a continuously high NeuroD1 expression forces the reprogrammed neurons into the glutamatergic subtype; the generation of other neuron types may require reducing NeuroD1 expression at the next stages of transformation. To perform this, the authors added miR-124 binding sites to the construct expressing mouse *Neurod1* gene. This way, when cells start neuronal differentiation, they begin expressing endogenous miR-124, which binds with the vector and blocks the *Neurod1* expression from it. The authors compared the transformation efficiency of Retro-CAG-ND1-124T-GFP and Retro-CAG-ND1-GFP retroviral vectors. At 14 dpi, human astrocytes infected with these two viruses showed similar progress in transformation. In the cells transduced with Retro-CAG-ND1-124T-GFP, NeuroD1 downregulation during the maturation of iNs was confirmed. ND1-124T-GFP-reprogrammed neurons showed the higher expression of GABAergic neuron markers and lower levels of glutamatergic neuron markers compared to ND1-GFP-reprogrammed neurons [396].

## 5. Glia-to-Neuron Reprogramming and NSC Transplantation Strategies for Treating Epilepsy

### 5.1. Glia-to-Neuron Reprogramming Used in Experiments with Epilepsy Models

The growing body of glia-to-neuron transdifferentiation research suggests the idea of using this method in clinical applications to treat neuropathologies, including epilepsy. So far, there are only two experimental papers wherein astrocytes were used to generate iNs in animal models of epilepsy. In the first study, Zheng et al. used the pilocarpine model of epilepsy in Sprague-Dawley rats. The authors utilized an AAV9 vector to express *Neurod1* under the control of the GFAP promoter in reactive astrocytes. One week post-SE, they bilaterally injected a viral vector into the hippocampus. By the 6th week post-SE, they observed the transformation of infected astrocytes into neurons. Using immunohistochemical staining for GAD67, the authors demonstrated that the resulting neurons were predominantly GABAergic. Subsets of iNs were immunopositive for GABAergic interneuron subtype markers: PV, neuronal nitric oxide synthase (nNOS), SST, calretinin, calbindin, or cholecystokinin. Quantitative analysis revealed that the pool of lost interneurons due to epilepsy was replenished through astrocyte conversion. It was shown that the pool of reactive astrocytes remained unchanged during experimental manipulation, suggesting no disruption of their physiological function. Whole-cell patch-clamp recordings showed APs and synaptic events in generated interneurons. Chronic EEG recordings indicated a 90% reduction in seizures in rats receiving the viral injection. Cognitive impairments associated with epilepsy were assessed, and improved cognitive performance was noticed following virus administration. Furthermore, even in established chronic epilepsy (three weeks post-SE), viral therapy effectively reduced spontaneous seizure frequency and increased survival [364]. In the other study, Lentini et al. used the kainate model of MTLE-HS. KA was unilaterally administered into the mouse hippocampus. A retroviral vector encoding ASCL1, DLX2 and reporter protein DsRed was injected into the formed epileptic locus. The combination of ASCL1 and DLX2 was selected because previous studies of this group showed that it is effective for producing specifically GABAergic neurons from astrocytes [344,397]. The MoMLV-based retrovirus used by Lentini et al. transduces only proliferative cells. Employing the BrdU labeling method, the authors have proven the cessation of endogenous neurogenesis by the 5th day after KA injection, so only reactive glia could be infected. BrdU+ cells expressed glial markers such as Olig2, GFAP and IBA1, corresponding to OPCs, reactive astrocytes and microglia, respectively. The authors demonstrated that by 7 dpi, infected cells exhibited the morphology of immature neurons and expressed DCX. By 6 wpi, DCX was no longer detected, but transdifferentiated cells had a mature neuron morphology, formed dense networks throughout the DG, and expressed NeuN. To confirm the GABAergic nature of induced neurons, the authors repeated experiments using GAD67-GFP transgenic mice and showed the colocalization of DsRed and GFP fluorescence in the same cells. Using rabies virus-mediated retrograde monosynaptic tracing, it was shown that by 7 wpi, iNs received inputs primarily from granule layer cells, with minor contributions from entorhinal cortex neurons, septal nuclei, and the medial septum-diagonal band of Broca region. By 2 mpi, these interneurons extended efferent fibers with synapse-like structures across the entire DG within its boundaries. Thus, induced interneurons established bidirectional connections similar to native interneurons. Whole-cell patch clamp with optogenetic activation demonstrated functional inhibitory GABAergic synapses between iNs and the granule cells. Spontaneous synaptic inputs were recorded, indicating robust synaptic connectivity. Chronic EEG data from mice at 6–8 wpi and 3–4 mpi revealed approximately a twofold decrease in seizure number and duration at both time points. Enhancing the activity of iNs via chemogenetic constructs reduced seizures by nearly 90%. In a parallel branch of experiments, Lentini et al. demonstrated effective seizure suppression by grafting iNs produced in vitro into the epileptic hippocampus. Hence, this study showed the efficiency of glia-to-neuron reprogramming for epilepsy treatment by both gene therapy and cell therapy approaches [25]. This research group also published the detailed reprogramming protocol employed in their work [398]. The research of glia-to-neuron transdifferentiation in epilepsy models continues. The approach used by Lentini et al. was later modified by Marichal et al. They used the mutant ASCL1SA6 version of ASCL1, as described in [336]. Their preliminary results obtained using the ASCL1 mutant in the MTLE-HS mouse model have been recently presented at the Epilepsy Research Institute Symposium, BNA2025 [399]; these results are not published yet.

### 5.2. NSC Transplantation Used in Experiments with Epilepsy Models

We should also mention studies wherein NSCs were transplanted into an epileptic brain. While this strategy does not involve cell transdifferentiation, NSCs and neurogenic astrocytes have very similar properties (as was described in Section 2.5 and Section 3.1), so experiments using astrocytes or NSCs to produce iNs in epilepsy models may be compared. Miltiadous et al. worked with the mouse KA model of TLE. In their study, the source of donor NSCs was the SVZ of 5-day-old mice from the strain expressing GFP in all cells. In one experimental group, NSCs were transduced with an LV vector expressing insulin-like growth factor 1 (IGF-1) that has neuroprotective properties. NSC transplantation 4 days after the KA injection partially rescued neuronal loss, even in some parts of the contralateral hippocampus. This may be explained by NSCs secreting neuroprotective factors, increasing host neuron survival. Indeed, the neuroprotective effects were more pronounced in the group where NSCs overexpressed IGF-1. The transplantation of non-transduced NSCs also elevated levels of IGF-1 in the hippocampus; it was not established which cells (transplanted or host cells) increased their IGF-1 production. NSC transplantation reduced the number of reactive astrocytes in the hippocampus and partially restored the cognitive abilities measured in the Morris water maze. Most interestingly, abnormally increased endogenous neurogenesis, measured by the number of cells positive for Ki67 (marker of proliferation Kiel 67), in KA-treated mice was reduced to almost normal levels at 30 days post-transplantation. Immunohistochemistry showed that most GFP+ donor NSCs differentiated into the neuronal lineage: they stained positive for doublecortin (DCX), a marker of immature neurons. Unexpectedly, the fraction of DCX+ transplanted cells did not decrease between 30 and 60 days post-transplantation. At 60 days, ~35% of the grafted cells were positive for mature neuronal marker NeuN; more than 10% differentiated into glutamatergic neurons (EAAC1+). Surprisingly, no differentiation into the GABAergic phenotype (GAD+) was detected. No results related to seizure activity in experimental animals are reported in this paper [249]. Hattiangady et al. also used NSC transplantation in a rodent epilepsy model (KA-induced SE in rats). The source of donor NSCs was the hippocampi of E19 rat embryos; before grafting, cultured NSCs were labeled with 5′-Chloro-2′-deoxyuridine (CldU) and treated with brain-derived neurotrophic factor (BDNF). The grafting was performed 6 days after the SE. It was shown that NSC transplantation reduced spontaneous recurrent seizures observed at 3–8 months post-SE. It also improved recognition memory in the novel object recognition test and reduced depressive-like behavior in the forced swimming test 6 months post-SE. Immunohistochemistry at ~9 months post-SE showed that grafted CldU-labeled cells survived in the hippocampus, mostly staying at the place of injection. About 20% of CldU+ cells were NeuN+, and ~17% were GABA+ interneurons. However, most grafted cells differentiated into glial cell types: ~40% of CldU+ cells were identified as S100β+ astrocytes, 20% as NG2+ OPCs, and ~13% as O4+ oligodendrocytes. Some grafted cells stained positive for neurotrophic factors: FGF-2, IGF-1, BDNF, or GDNF. The neuroprotective effect of NSC grafts after SE was demonstrated as a less prominent loss of NPY+ or PV+ interneurons in the treated group. Importantly, NSC grafting promoted normal hippocampal neurogenesis (estimated by the number of DCX+ cells), reduced aberrant neurogenesis (migration of newborn neurons into the dentate hilus and the number of PROX1+ granule cells in the hilus) and reduced aberrant mossy fiber sprouting [313].

The studies described in Section 5.1 and Section 5.2 used different rodent species, different epilepsy models and different approaches to produce iNs, including the starting cell type. However, a decrease in epileptic activity and/or improvement of behavioral comorbidities associated with epilepsy was reported for all these differing protocols. On one hand, this highlights the high potential of direct neuronal reprogramming as a therapy strategy and the importance of its further research. On the other hand, it may be possible that some part of the observed effect is caused by mechanisms other than the replacement of lost interneurons. The idea of NSCs secreting neuroprotective factors as one of such mechanisms was proposed and partially experimentally confirmed in the study by Miltiadous et al. [249]. Moreover, it is possible that the neurogenic treatments used in the experiments with transdifferentiation in fact also affected resident NSCs. Neither the GFAP promoter used by Zheng et al. [364] nor the specific targeting of proliferating cells used by Lentini et al. [25] can reliably discriminate reactive astrocytes from NSCs (a more detailed description of such methodological problems is provided in Section 6.4). To exclude NSC contribution, Lentini et al. employed BrdU labeling to confirm that endogenous neurogenesis in their model is stopped, and proliferating cells targeted by their virus only express glial markers; however, GFAP (also expressed by NSCs) is among those markers. This strategy leaves the possibility of NSCs proliferating while undergoing self-renewal, or switching from neurogenesis to gliogenesis (this switch has actually been confirmed in the KA epilepsy model [400]) being still targeted.

**Figure 3:**
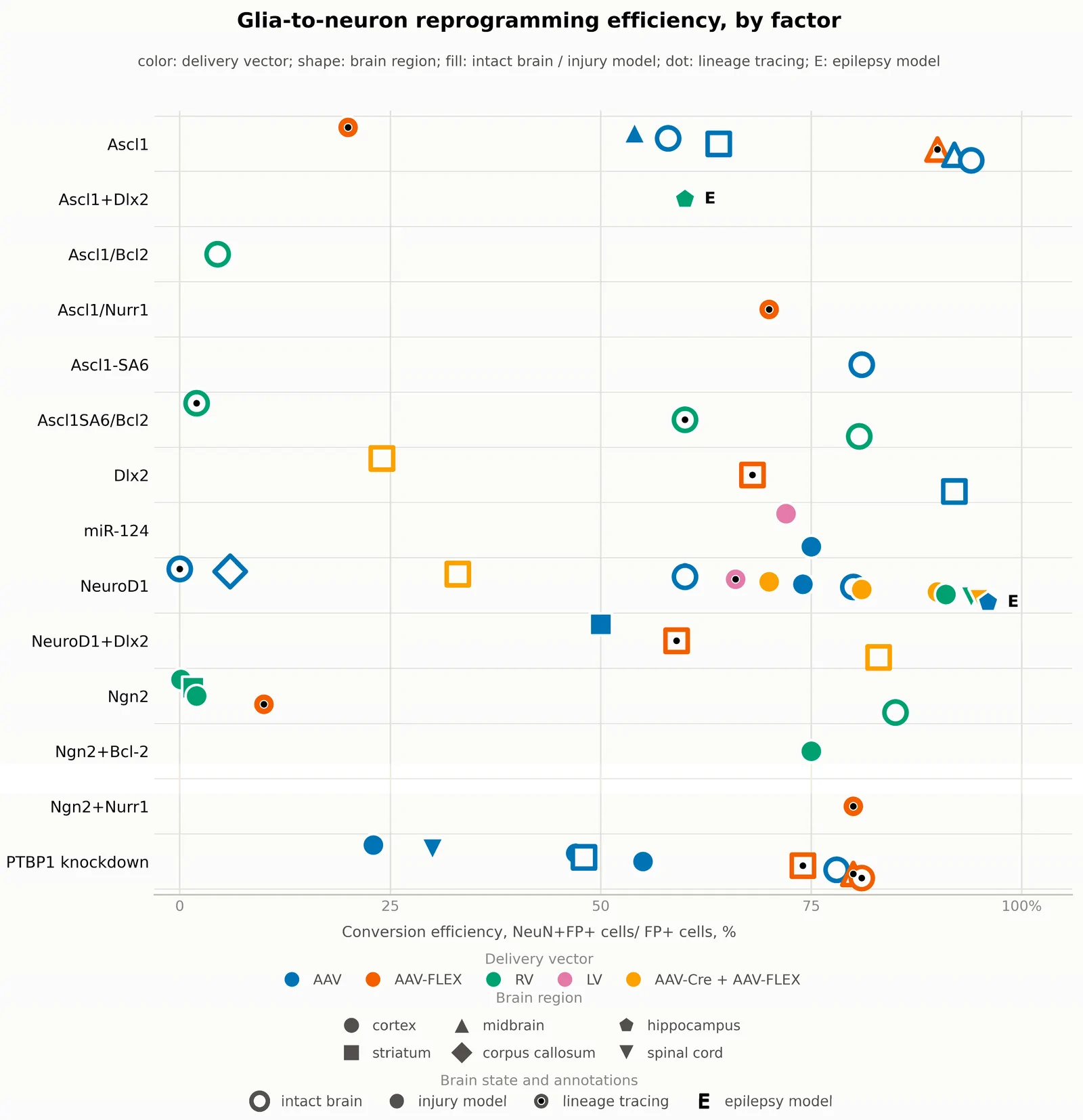
A chart of reprogramming efficiencies in experiments in vivo from the papers described in Section 4 and Section 5. Each point represents one experiment. Efficiency is calculated as the ratio of NeuN+, FP+ cell number to FP+ cell number, where FP is the fluorescent protein used as the marker of transduction in the corresponding study. The symbol color denotes the type of the viral vector, and the symbol shape denotes the virus injection region. Empty shapes represent experiments with intact brain, filled shapes represents experiments with neuropathology models. Dots inside symbols signify that a lineage tracing method was used; “E” near a symbol marks studies with models of epilepsy. The data shown on the graph and described in the legend are available in Supplementary Table S1, which also contains some additional information (the reference, the type of injury, and the time after infection); Supplementary Code S1 was used to plot the graph. Table S1 was compiled through a manual review of the cited articles, including their figures and Supplementary Materials. Studies in which the ratio of NeuN+, FP+/FP+ cells could not be determined were excluded from the analysis. We must note that efficiency numbers plotted here are not directly comparable, since different studies used different vectors, brain regions, time points and lineage tracing strategies. FP expression in resident neurons that may happen with less stringent lineage tracing would increase the observed number of NeuN+, FP+ cells.

Table 2 and Figure 3 summarize the information described in Section 4 and Section 5.

## 6. Discussion: Considerations for Future Development and Clinical Applications of Epilepsy Therapy with Glia-to-Neuron Transformation

The studies cited in previous sections show that a few types of glial cells may be used for neuronal conversion. The remarkable plasticity of astrocytes, their similarity to NSCs and their inherent neurogenic capacity make them the most promising cell target for neuroregeneration therapy. OPCs are less commonly used for neuronal conversion, both because they are less well-studied than astrocytes and because they most likely do not produce neurons naturally. However, they may be easily manipulated to either produce or become neurons. Microglia may also be used for artificial transdifferentiation; one argument for this is the abundance of microglial cells in brain lesions, but so far there are only a few studies of microglia-to-neuron conversion. NSCs or similar cell types may be transplanted into damaged brain regions with or without their forced neuronal differentiation to replenish the neuronal loss.

### 6.1. Specific Properties of the Epileptic Brain in the Context of Glia-to-Neuron Conversion

Importantly, while brain lesions in epilepsy may be very different, reactive gliosis is always observed in the epileptic brain [4,59,60], and many studies of astrocyte-to-neuron transformation used specifically reactive astrocytes as the starting cell type [25,177,262,318,340,358,364,401]. There is evidence that reactive astrocytes are easier to transform compared to resting astrocytes, both in vitro and in vivo [28,330,355]. Some authors also specify that reactive astrocytes have to be actively proliferating for a more successful transformation [318,355]. Other common properties of reactive astrocytes in epilepsy that may be exploited for making them cell targets for therapy, including transdifferentiation-based therapy, are their abundance [129] and their location close to the BBB [59], which arguably would make systemically injected treatments (like the ones used in [28,32]) more effective.

Since the inherent neurogenic potential of astrocytes in both intact [256] and injured [43,261] brain parenchyma, as well as astrocyte susceptibility to induced neuronal conversion [327], were shown to sharply decrease with age in rodents, it may be assumed that the efficiency of glia-to-neuron transformation in epilepsy would be higher in very young patients.

Brain injuries that may cause epilepsy, as well as histopathological findings in epilepsy, are diverse, so there are conditions that may facilitate glia-to-neuron transformation but are present only in some epilepsy cases. Since astrocyte neurogenic potential was shown to differ between brain lesion types [43,268], it is possible to assume that artificially induced glia-to-neuron conversion would be more effective for epilepsy caused by an invasive brain injury, such as TBI or stroke. However, this consideration is applicable only in the hypothetical case of seizures starting shortly after the injury, and being so severe that they quickly cause neurodegeneration. Often, the development of epilepsy after TBI or stroke takes a long time [4], and the loss of neurons as a result of seizures usually happens much later [183,402], so by the point where neuroregeneration therapy is considered, the microenvironment in the affected region would not be the same as recently after the injury. In conditions with slow chronic neurodegeneration, factors modulating neurogenesis, including inflammation and astrogliosis, differ from acute injuries when multiple neurons die synchronously [34,273].

While seizures may not be immediately triggered by TBI or stroke, both these injuries cause the formation of a long-lasting glial scar which, in turn, may become the cause of seizures [78]. Neuronal transdifferentiation induced by NeuroD1 [358], SOX2 [294], Ngn2 [373] or PTBP1 downregulation [29,389] was reported to be effective in conditions with a prominent glial scar. Interestingly, the glial scar formed by astrocytes was reduced in the study where neurogenesis was induced not in astrocytes but in OPCs, suggesting complex mechanisms regulating astrogliosis during the changes in the surrounding tissue [294]. In the case of epilepsy caused by a glial scar, the transformation of astrocytes within the scar into neurons (or scar reduction happening as a “side effect” of OPC transformation) may be a better alternative to a scar-resection surgery. Even though a glial scar is a known cause of seizures, it is a relatively rare finding in epilepsy. On the other hand, hippocampal sclerosis, a condition that also involves extensive astrocyte proliferation, is much more typical for epileptic brain [60]. We may speculate that glia-to-neuron conversion may also be effective in a sclerotic hippocampus; however, currently, experimental data from using transdifferentiation methods in this condition are lacking.

Hemorrhage is another condition that may be present but is not universally found in epilepsy. A recent work by Sirko et al. demonstrated that epileptic brain samples have a particularly high number of neurosphere-forming cells in regions with reactive gliosis combined with intraparenchymal hemorrhage [268].

The epileptic focus may be located in different brain regions; however, there is very little evidence for epileptogenesis in the striatum [403]. In a rare case like this, transdifferentiation-based therapy very likely would be more effective due to the neurogenesis-favoring microenvironment in the striatum [160]. The typical locations of the epileptic focus are the hippocampus or the cortex. Out of these, the hippocampus is likely more permissivive to neurogenesis since it contains a neurogenic niche, SGZ, which may provide a gradient of neurogenic factors to the neighboring regions (see also Section 6.3).

Importantly, some of the clinically used or currently researched anti-seizure drugs may also facilitate glia-to-neuron conversion. VPA, a histone deacetylase inhibitor and a common anti-convulsant, promotes normal neurogenesis and also facilitates the generation of iNs from different types of glia [276,311,333,367]. The inhibition of oxidative stress and ferroptosis is beneficial for both reducing neuronal damage in epilepsy [86,87] and for increasing glia-to-neuron transformation efficiency [315]. It should also be noted that some anti-seizure drugs may have an additional effect of reducing neuroinflammation, which would affect neuroregenerative therapies of epilepsy in different ways (see Section 2.4.1).

It seems logical that any approach to convert reactive astrocytes into neurons in the epileptic brain would serve a dual purpose: it would not only generate new neurons, but also decrease the number of reactive astrocytes and thus diminish their contribution to epileptogenesis by the mechanisms described in Section 2. However, it seems like beneficial effects not directly related to replenishing lost neurons have been observed only for some neuroregeneration strategies. Different authors pointed out that NeuroD1-induced astrocyte-to-neuron conversion in models of different pathologies had additional positive effects, like the decrease of A1 astroglia, reactive microglia and neuroinflammation in models of TBI [358] or ischemic brain damage [326], or the increased survival of preexisting neurons in models of ischemic brain damage [326,359]. The exact mechanisms of these effects are yet to be studied. Another important “side effect” reported for NeuroD1 transdifferentiation therapy is the normalization of synaptic plasticity after stroke [27], which is crucial for possible applications of such therapy in epilepsy. NSC transplantation early after the onset of epilepsy also increased the survival of preexisting neurons, and normalized learning and memory in rodent models, with the likely mechanism being the secretion of neuroprotective factors by the grafted cells. Notably, NSC grafting in epilepsy models not only promoted normal neurogenesis, but also reduced aberrant neurogenesis [249,313].

To conclude, if the transdifferentiation strategy is eventually approved for clinical use in epilepsy, its applicability should be estimated on a case-by-case basis, considering the individual history of the patient, including their age, the initial injury that triggered seizures and the presence of findings such as hippocampal sclerosis, glial scar or hemorrhage. It should also be considered that anti-seizure drugs may increase the transdifferentiation efficiency, and that neuroregeneration treatments may have beneficial effects beyond generating new neurons.

### 6.2. Different Neuronal Subtypes as a Result of the Conversion

In many proof-of-principle studies of direct neuronal conversion, only pan-neuronal markers like Tubb3 and MAP2 were used to confirm the neuronal identity of the induced cells (see Table 1). However, there are multiple kinds of neurons, and in different neuropathologies the desired outcomes of transformation are different. Moreover, not all neuronal subtypes may be easily generated from other cell types. In the simplest scenario, a cell type-specific TF may be used to efficiently produce neurons of this type, as was shown for DLX2 and GABAergic neurons [338,378]. In some cases, manipulations with the expression level of the same TF may affect the outcome of transformation [322,396,404]. The microenvironment in the brain region where the conversion is initiated is also important for determining the phenotype of iNs (described in the next section). In some cases, however, the result of neuronal transdifferentiation mostly depends on the starter cell type. Pereira et al. tested multiple combinations of pro-neural genes and fate specifiers for the reprogramming of striatal OPCs into different types of neurons, with the aim being to generate dopaminergic neurons, which was not achieved. The authors reported that OPCs tend to transform into neurons similar to fast-spiking PV+ interneurons, independently of the combination of reprogramming factors used [347]. This is corroborated by the results of Guo et al. who noted that after the same manipulation, namely, the overexpression of *Neurod1*, OPCs more often than astrocytes give rise to GABAergic neurons in the cortex after a stab wound [318]. This may be explained by the close proximity in lineage distance between at least some populations of OPCs and GABAergic neurons: progenitor cells in the embryonic ganglionic eminences produce both these cell types [379]. The proclivity of OPCs to turn into interneurons independently of the neuronal transformation method used means that OPCs are not a good starting cell type for neuroregeneration therapies of PD. However, it also means that OPCs may be exactly what is needed for epilepsy therapies, because these are usually aimed at restoring the loss of GABAergic interneurons; indeed, different currently researched stem cell repair strategies for epilepsy are supposed to generate GABAergic neurons [24].

Nevertheless, we must note that GABAergic interneurons are not strictly “anti-epileptogenic” per se, and there are some mechanisms by which they may incite or promote seizures [405,406]. GABA signaling upregulation has been reported in diverse animal models of seizures and epilepsy [101,407,408,409]. The major function of fast-spiking PV+ basket cells is the synchronization of network activity, so under some circumstances, enhanced GABAergic signaling might trigger seizures [410]. Inhibitory interneurons can promote synchronization by a GABAergic depolarization resulting from the imbalance of chloride, or by a synchronous recovery from the inhibition of a larger population of principal neurons [411,412,413]. Using reactive astrocytes of the epileptic brain to transform them into GABAergic neurons makes the situation even more complicated, considering epilepsy-associated changes in GABA release from astrocytes (Section 2.4.5) and astrocyte interactions with ECM, which are particularly important for the survival of some types of GABAergic neurons (Section 2.4.6).

It should also be considered that with iNs, confirming the expression of cell-type-specific markers may not be a sufficient proof that these cells are fully functionally identical to resident neurons of this subtype. For example, Cooper et al. showed that while ASCL1SA6 induction generates iNs with some hallmarks of GABAergic interneurons, these cells fail to activate a key gene expression module, *Dlx* family, and migrate to atypical positions within the cortex [337]. Ideally, the iN phenotype should be assessed by multiple methods, as was done, for example, in the study by Zhou et al., where the dopaminergic phenotype of iNs was confirmed by staining for proteins involved in dopaminergic transmission, electrophysiological tests and experiments where the uptake and release of a fluorescent dopamine analogue were measured [388].

To summarize, GABAergic neurons seem to be a neuron subtype that is relatively easy to generate by glia transdifferentiation approaches; however, it is possible that iNs may be functionally different from resident GABAergic neurons. GABAergic signaling in epilepsy is dysregulated by multiple mechanisms involving both neurons and glia, and an excessive activity of GABAergic neurons may contribute to epileptogenesis, just like their insufficient activity. This complicated picture means that the effect of GABAergic iNs in epilepsy models may be unpredictable. Thus, any strategies of generating GABAergic neurons in situ in the epileptic brain need to be evaluated by functional tests of seizure susceptibility in vivo.

### 6.3. The Role of Brain Region (Microenvironment and/or Regional Identity of Glial Cells) in the Conversion Outcome

Another factor that may affect the outcome of neuronal conversion in situ is the brain region. For example, Liu et al. obtained different combinations of iN subtypes after *Neurod1* expression from the same vector in the cortex or striatum, and this vector failed to convert astrocytes into neurons in the corpus callosum [357]. It was easier to generate iNs in the striatum as compared to the cortex when NeuroD1 [28] or Ngn2 [314] was used. Conversely, ASL1-induced astrocyte-to-neuron conversion was shown to be more effective in the cortex than in the striatum [325]. Xu et al., who transformed Müller glia into two different types of retinal neurons with NeuroD1, suggest that the change of location of the injection may be one of the reasons for different outcomes [404]. One of the explanations for such differences between CNS regions is the microenvironment (local exposure to signaling molecules) affecting the outcome of glia-to-neuron transformation. A strong argument for this is that not only the region but also the injury conditions affect the outcome of the transformation, in terms of both the number and the type of iNs [314,349]. A direct proof of the role of the microenvironment in neurogenesis by astrocytes is given by the experiment with *Rbpj* deletion in all astrocytes throughout the brain; only astrocytes in the medial striatum completed the neurogenic program activated by this manipulation. The authors hypothesized that SVZ, being located close to the medial striatum, provides a gradient of neurogenic factors into this region, and establishes a more neurogenesis-permissive environment there [160]. Notably, there are some other examples when the astrocyte-specific knock-out of a gene caused different effects depending on brain region (reviewed in [257]).

While the role of microenvironment in glia-to-neuron conversion has been confirmed, the clonal origin (regional identity) of astrocytes chosen for transformation may be even more important. Astrocytes display inter-regional diversity: in a transcriptomic study where astrocytes from 13 different regions of mouse CNS were compared, only 825 out of 4314 astrocyte-enriched genes were shared between all studied regions [414]. Hu et al. demonstrated that murine astrocytes derived from different brain regions have different degrees of susceptibility to neuronal reprogramming in vitro. They used an LV vector expressing *Ngn2* to convert early postnatal (P2–P4) astrocytes to neurons, and estimated the efficiency of reprogramming as 50%, 30% and 20% for astrocytes taken from the cortex, cerebellum, and spinal cord, respectively. A similar difference between these regions was observed for adult cultured astrocytes, but their conversion efficiency was much lower. When *Ascl1* expression was used as the transformation method, differences in the conversion rate between astrocyte groups were similar to what was observed with Ngn2. However, the type of iNs depended on the transformation factor and not on the astrocyte origin region; cells from different regions generated mostly glutamatergic neurons when Ngn2 was used, and mostly GABAergic neurons when ASCL1 was used. Next, the authors aimed to identify the mechanisms underlying regional differences affecting astrocyte-to-neuron conversion. Astrocytes from different regions have different intrinsic proliferation rates; however, experiments with proliferation blockade showed that it does not affect the efficiency of neuronal transformation induced by the factors used in this study. The screening of multiple transcription factors and key signaling pathway proteins demonstrated the difference in expression levels of Notch1 signaling-related proteins correlating with transformation efficiency (see Section 4.8 for a description of Notch1-related experiments in this study). However, the authors note that the Notch1 signaling blockade caused a relatively small increase in the reprogramming efficiency, suggesting that other unidentified factors may also be important for this [327]. Herrero-Navarro et al. showed that in the neocortex and thalamus, neurons and astrocytes express shared region-specific transcriptional and epigenetic signatures. Multiple genes (among them some TFs and genes known to confer regional neuronal identity) that are differentially expressed between neocortical and thalamic neurons are also differentially expressed between neocortical and thalamic astrocytes. More detailed analysis showed similar patterns at the subregional level (between different thalamic nuclei and different parts of the cortex). It was shown that region-specific gene expression is maintained following astrocyte-to-neuron reprogramming by *Ngn2* and *Bcl2* expression, both in vitro and in vivo (in early postnatal mouse brain). Co-culture experiments have shown that the regional identity of iNs is largely cell-autonomous; the presence of cells from a different brain region in the same culture did not change it. Importantly, the authors were able to manipulate this regional-specific code; a co-expression of a single thalamus-specific gene *Gbx2* together with *Ngn2* in cortical astrocytes resulted in a shift towards thalamic identity in iNs, even though only a partial change of region-specific gene expression occurred [335]. Interestingly, the brain region may not be important at all to the result of the neural conversion of OPCs; Pereira et al. note that the PV+ interneurons that they generated from striatal OPCs normally comprise less than 1% of striatal neurons. Moreover, they obtained iNs with a similar phenotype by transforming OPCs in midbrain [347].

To conclude, when designing strategies for neuronal transdifferentiation to treat epilepsy, it is important to consider the target brain region where neurons have to be restored. In many cases, the optimal strategy would be transdifferentiation in situ within the same region; however, a situation where astrocytes collected from some other region and transdifferentiated in vitro would be more useful is conceivable.

### 6.4. Lineage Tracing, Cell Type Identification and Estimation of Transformation Efficiency

The development of any novel therapeutic approach that includes in situ neurogenesis should make it possible to perform the monitoring and precise phenotypic characterization of subpopulations of cells undergoing transdifferentiation [320]. One important methodological problem is the possibility of the wrong identification of preexisting mature neurons as iNs when the expression vector has a leaky promoter [331,350,363,390,391,392,415]. It should be noted that AAV vectors are routinely used in neuroscience, including studies of glial cells; however, many AAV serotypes are known to preferentially transduce neurons [416,417], which presents a problem when gene delivery exclusively to glial cells is needed. Interestingly, Wang et al. showed that some AAV serotypes, when used with an astrocyte-specific promoter, show a very high specificity of transgene expression in astrocytes shortly after the infection (>90% of labeled cells are astrocytes at 4 dpi), but a noticeable expression in neurons starts later (<70% of labeled cells are astrocytes at 14 dpi) [363]; in most in vivo transdifferentiation studies, the result is estimated at 14 dpi or later. In addition, it was shown that promoters that are considered universal (including CAG, EF1α, CMV), when used in a virus injected into the rat brain, drive highly specific expression in neurons, with few or no astrocytes expressing the transgene (for the LV vector used in this study, neuronal tropism is debated) [418]. The use of cell type-specific promoters for glial cells may not significantly improve the outcome. In particular, the GFAP promoter (the most common for astrocyte targeting) raised some concerns; for example, Papadimitriou et al., who used a reporter mouse strain with a virus expressing Cre under the GFAP promoter, noted that the reporter was unexpectedly expressed in resident neurons besides its expression in astrocytes [340]. A similar problem, a significant level of reporter expression in resident neurons when the GFAP promoter is used, was reported by Fukui et al. [30] and Ghazale et al., who could not achieve astrocytic specificity even with different combinations of AAV serotypes and GFAP promoter variants [350].

NeuroD1 overexpression and PTBP1 downregulation are the two neuronal conversion strategies with the highest number of studies disproving previously reported results. With NeuroD1, results that were not reproduced by other groups include microglia-to-neuron transdifferentiation. Notably, for NeuroD1, multiple studies also report protective effects on preexisting neurons (see Section 4.4), while for PTBP1 downregulation, it was suggested that the targeted cell types may be NSCs together with astrocytes, or even exclusively NSCs (Section 4.9). Thus, with these strategies, a rescue of symptoms in a disease model may happen even if new iNs were not in fact produced by glial cells. Table 3 summarizes how many of the in vivo studies applying these strategies and cited in the present review used either methodological approaches that can produce false positive results or additional lineage tracing that should prevent this. We must note that the viral or genetic tracing of astrocytes very often utilizes the GFAP promoter, so all the concerns about this promoter being not highly astrocyte-specific apply here as well. It seems like the papers in Table 3 that reported a successful astrocyte-to-neuron conversion either did not use any form of lineage tracing, or used GFAP promoter for tracing.

One way to improve the glial cell targeting and avoid the misinterpretation of results is to prevent the viral vector from infecting neurons altogether. A recent example is the study by Liu et al. who used a modified AAV capsid with an astrocyte-targeting P1 peptide [29]. An alternative approach to improve astrocyte targeting is adding miRNA-binding sites to the expression vector. Gleichman et al. developed a cassette of four copies of six miRNA-targeting sequences that triggers transgene degradation specifically in neurons and endothelial cells, and greatly increases the astrocytic specificity of transgene expression [419]. To target OPCs (which comprise the vast majority of proliferating cells in the intact brain), retroviruses that only infect dividing cells may be used, such as in the study by Bazarek et al. [329]; this would assure that no resident neurons are infected. However, in a damaged brain, astrocytes and microglia also proliferate and would be infected by such vectors, so this method is only usable for proof-of-principle experiments, not for disease models.

A somewhat similar technical problem complicates the discrimination between astrocytes and NSCs as the starting cell type, because their molecular markers overlap significantly [153,165] and the identity and properties of intermediate differentiation stages of the astrocyte lineage are still not well understood [253]. At least one study demonstrated that the increased neurogenesis and memory improvement observed after treatment (antisense oligonucleotides against *Ptbp1* transcript, in a mouse line with labeled GFAP-expressing cells) is a result of NSC activation. In this study, GFAP+ NSCs were discriminated from GFAP+ astrocytes by their morphology, position and GFAP distribution inside the cell [31]. In another study, the authors suggest that *Ptbp1* knockdown targeted to GFAP-expressing cells could stimulate neurogenesis by both astrocytes and NSCs [30].

**Figure A1:**
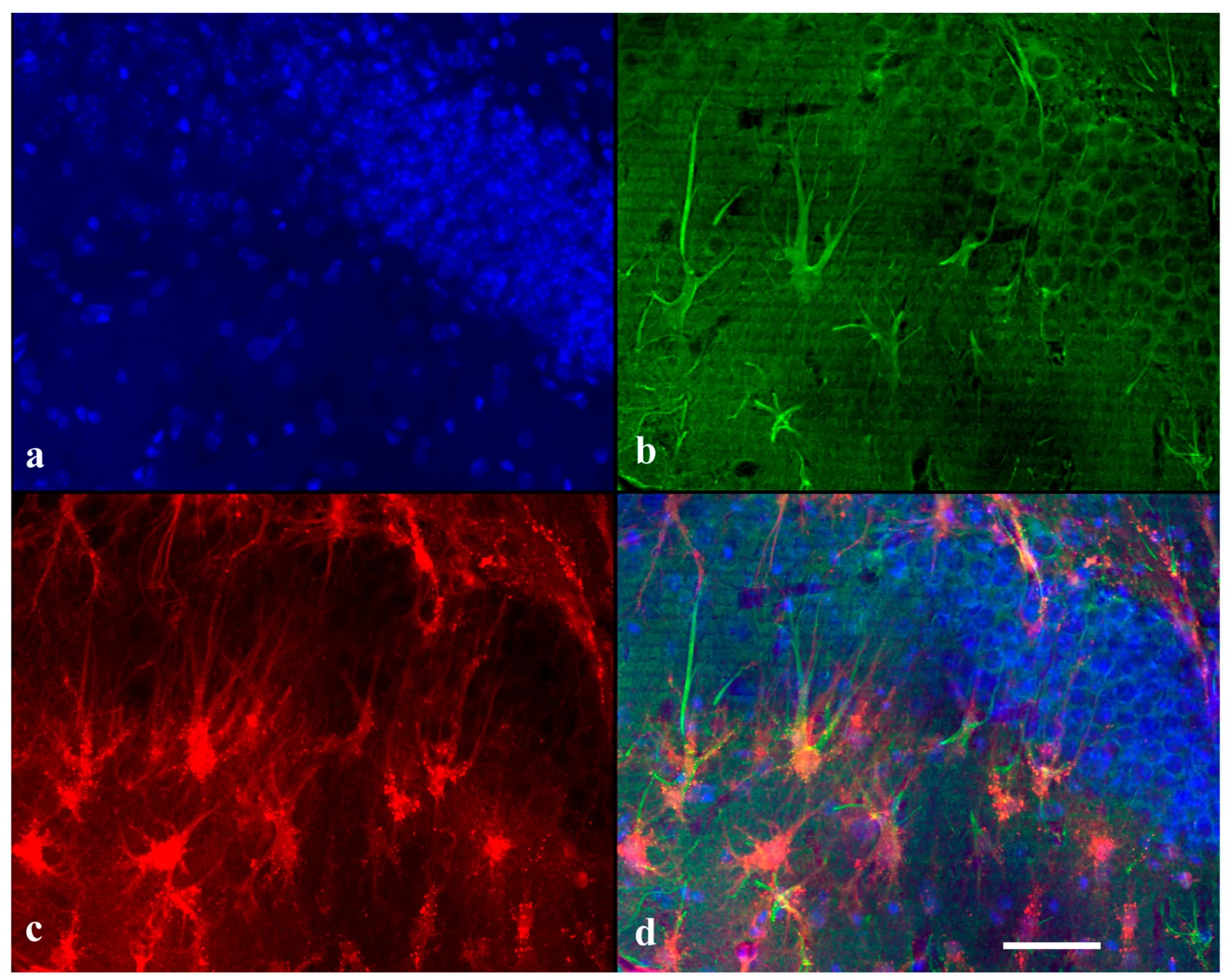
An illustration of reactive astrocytes staining positive for GABA. Results from our lab. Hippocampus of a mouse (C57Bl6, male, 3 months old) with KA-induced epilepsy, photographed far from the KA injection point. This epilepsy model includes a loss of GABAergic neurons in the hippocampus. Slice thickness is 50 µm. Confocal microscopy. DG (upper right corner) and hilus (bottom) are shown. The scale bar is 50 µm. (**a**) Nuclei stained with DAPI. (**b**) Immunostaining for GABA (antibody Sigma-Aldrich A2052, 1:500). (**c**) mScarlet fluorescence in cells with active GFAP promoter (viral labeling by GfaABC1D::mScarlet; AAV9 serotype, 2 × 10^12^ vg/mL). (**d**) Merge. The colocalization of GABA (green) and mScarlet (red) is clearly seen in astrocytes in the lower left corner of the view.

In addition to being expressed by other cell types, GFAP, the most commonly used astrocyte marker, is also not expressed by all astrocytes, because they are highly heterogeneous. Hu et al. compared the protein expression levels of GFAP and Aldoc (aldolase C, a more universal astrocyte marker) in different CNS regions, and showed that even in the spinal cord, where the ratio of GFAP+/Aldoc+ cells to Aldoc+ cells was the highest among the studied regions, it was still less than 50% [327]. Other standard cell type markers may also not represent the cell type very well. Similarly to GFAP in astrocytes, NeuN is the most commonly used marker for mature neurons. However, NeuN is not expressed by some neuron types. In neurons that normally express NeuN, it may be downregulated in some neuropathologies, including epilepsy [420], which may be misinterpreted as neuronal death [421]. Even GABA may not be a good marker for GABAergic neurons when astrocyte-to-neuron transformation is considered, because reactive astrocytes (which would be present near the injection site even in a healthy brain) are able to produce GABA [422,423], a phenomenon we have observed in our own lab (Figure A1); see also Section 2.4.5. Moreover, some astrocytes express NG2 [278], and some subpopulations of microglia express NG2, nestin or vimentin [279], all routinely used as markers for other CNS cell types.

While there are many indications that actively proliferating glial cells are more susceptible to neuronal conversion, at least one study showed that BrdU-labeled cells had a low efficiency of reprogramming. However, the authors suggest that this may be an artifact of using BrdU to label proliferating cells [29]. Indeed, experiments with cultured cortical astrocytes showed an unexpected inhibition of astrocyte-to-neuron conversion by BrdU [424]. This observation suggests that in many studies, including papers cited in the present review, neuronal conversion efficiency may be underestimated because BrdU is widely used for cell fate tracing in this research area.

To summarize, it is always important to consider what percentage of the target cells would express the protein chosen as a marker, what other cell types may express it in the chosen experimental model, and what other markers and labeling strategies may be used to prevent errors in cell type identification. Cell morphology and position are important; however, they are expected to change in transdifferentiation experiments, so it is more reliable to combine morphological cell identification with labeling based on gene expression. It is preferable to use multiple cell type markers instead of one. Choosing a cell-type-specific promoter should follow the same logic, but in this case using multiple genes instead of one requires complex genetic engineering manipulations. In the case of Cre-based methods, a transient activation of the chosen gene promoter in progenitor cell types should be excluded, since it may unexpectedly cause labeling [293] or a gene knock-out [110] in a different mature cell type than was planned.

Reliably identifying and/or targeting astrocytes, in particular, is a very complicated task; it seems like all commonly used astrocyte markers are expressed by some other cell types, especially NSCs [153], while at the same time no single promoter is able to strongly drive expression in all of the diverse astrocyte subpopulations [415]. Moreover, the problem mentioned above with Cre activation in progenitor cell types may also make it impossible to distinguish NSCs and mature astrocytes that originate from NSCs by Cre-based methods. Importantly, for the KA model of epilepsy in particular, it was demonstrated that NSCs in the SGZ switch from neurogenesis to producing astrocytes [400], which even further complicates the distinction between these cell types. In the case of NSCs being an actual target of a neurogenic treatment instead of astrocytes, the results showing beneficial effects of the treatment would still be valid and even translatable to clinical practice. However, the mechanisms of the treatment would be interpreted wrongly, which would affect the design of future studies and may lead to a waste of resources on irrelevant experiments.

### 6.5. Confirming Functional Integration of Induced Neurons

Another problem that makes it necessary to perform a strict monitoring of the fate of iNs is their possible incorrect integration into neuronal circuits. It was demonstrated that even iNs that reached an advanced maturation stage may occupy atypical locations in the brain [337]. Arguably, this problem may be more important in treating epilepsy compared to many other neuropathologies, since it is known that in the epileptic brain, there are abnormalities in the integration of seizure-born new neurons [191,193], including heterotopic neurons [59], pathological neuronal circuit remodeling [72] and even aberrations in connections between brain regions [12,13].

Thus, it is not enough to confirm the successful neuronal conversion of the glial cells or even their differentiation into the target neuronal subtype; it is also necessary to verify their integration and correct function within the neuronal network. Anatomically, such integration may be estimated by retrograde monosynaptic tracing, which was used, for example, in [25,346]. Electron microscopy may also be used to assess synapses formed by iNs, as was done, for example, in [270]. Physiologically, the electrical activity of the cells within the restored circuits may be recorded. For example, Jiang et al. used MultiElectrode Array recordings in brain slices to reveal that iNs restored interrupted cortical circuits and synaptic plasticity [27]. Ideally, anatomical and physiological tests of iN integration should be combined. One example of this is the work of Chen et al., who used a combination of patch-clamp recordings of acute spinal cord slices and microscopy to estimate the number of synapses in the iNs, and came to a conclusion that these neurons are immature and have limited synaptic connectivity [355].

Interestingly, multiple studies showed that new neurons generated locally in the striatum or retina after an injury have a short lifespan; however, it was speculated that these transient neurons may still contribute to recovery after the injury by some forms of compensatory plasticity and/or by exerting protective roles for neurons that have lost their targets [159,242,272,273]. It should be noted that, in a healthy brain, most newborn neurons generated in adult neurogenic niches do not survive beyond a few weeks [425,426,427], and immature neurons perform some specific functions in transient plasticity [154]. Similarly, in the developing CNS, immature neurons (including cell types that do not survive into adulthood) have important functions in circuit formation [428,429,430]. An even more complicated situation is possible wherein, despite their transient life, lesion-induced neurons mature morphologically and functionally, integrating into circuits for a short time [272].

To conclude, for a confirmation that the iNs rescue the neurological deficits of the model phenotype, it is always preferable to perform behavioral tests with animals in addition to registering the changes in the brain on the cellular level. It should also be noted that even confirmed phenotype improvement may not be the result of iNs replacing lost neurons, but may be attributed to some other mechanisms triggered by transformation factors, for example, the secretion of neuroprotective molecules. Specifically, for PTBP1 downregulation, one proposed mechanism of phenotype rescue in the case of the virus targeting resident neurons is their increased survival as a result of the modified splicing of some mRNA targets [29,431]. Indeed, in some studies, behavioral or locomotor improvement was reported, but it cannot be mechanistically linked to the restoration of lost neurons in the targeted CNS region, since this region does not contribute to this specific behavior (reviewed in [432]). Such results are still useful for designing treatments for neuropathologies, but wrong assumptions about their mechanisims would hinder the improvement and refinement of these treatments (increasing their efficiency and overcoming undesired side effects).

### 6.6. Other Complications That Should Be Considered with Neuronal Transdifferentiation

Gene therapy in general has an inherent problem of dosage; the distribution of transfected cells and the expression level of the target gene cannot be changed after the therapy has been given, which could lead to under- or over-dosing [433]. An interesting approach to overcoming this was applied by Mahmoudi et al. who used a special hydrogel for a controlled release of an AAV vector carrying the *Neurod1* transgene for the neural reprogramming of reactive astrocytes. Controlled release mitigates the issues of high viral dosage at the target site, off-target delivery, and immunogenic reactions. Experiments in vitro and in vivo confirmed the efficiency of astrocyte-to-neuron reprogramming with this method in a TBI model [401]. A somewhat similar approach with artificial fibrous matrices was used by Kim et al. to provide physical guidance for iNs and overcome the problem of these neurons not elongating their axons to the target site in a model of SCI [434].

With any treatments inducing cell proliferation or dedifferentiation, especially in a pro-inflammatory microenvironment, tumorigenesis should be considered as a possible undesired consequence. Indeed, there are notable similarities between NSCs in SVZ, astrocytes associated with injury, and astrocytes associated with cancer. Human gliomas express reactive and neurogenic astrocytic cell markers [162]. Moreover, glioblastoma is known as a tumor with a particularly high cell heterogeneity; glioblastoma cells execute neurodevelopmental programs and display a continuum of “differentiation” into states resembling different CNS cell types. Another important factor in glioblastoma formation is injury response programs [435]. Astrocyte dedifferentiation into stem-like cells in response to injury is normally rare, but it is greatly facilitated in astrocytes with a mutation of tumor suppressor protein p53 [436]. Wang et al. also showed that the p53 pathway’s downregulation increases the efficiency of the SOX2-induced reprogramming of astrocytes into neuronal precursors [368]. However, as expected, p53 dysfunction is also a key factor for tumorigenesis by astrocytes: while *NANOG* overexpression in normal astrocytes is sufficient to convert them into NSCs [312], in p53-deficient astrocytes, the same manipulation transforms them into cancer stem-like cells [437]. To make sure that a treatment is not tumorigenic, long-term experiments may be required. For example, the group that worked with SOX2 kept observing experimental animals for signs of tumorigenesis for a year in one study [367] and for 15 months in another study [368]. Based on the similarities between NSCs and reactive astrocytes, inflammatory response may be another undesired side effect of inducing neurogenic programs in astrocytes. Indeed, Liu et al. registered neuroinflammation in the corpus callosum in experiments with DLX2-mediated transdifferentiation in the striatum. Interestingly, a co-expression of *Neurod1* with *Dlx2* inhibited this inflammation. Based on immunohistochemistry results, the authors supposed that DLX2 alone triggers a partial conversion of white matter astrocytes to an intermediate state associated with neuroinflammation, while the addition of NeuroD1 reverses this partial conversion [377].

Lastly, we have to point out the limitations of both in vitro and in vivo models relevant to this theme that may affect the translational potential of the results. Cell cultures may be a poor model for processes happening in the brain. Cultured astrocytes often acquire artificial functional properties [15,37]. Astrocytes in culture without neurons show signs of reactivity even in the absence of specific stimuli [34]. Reactive astrocyte migration towards a wound was demonstrated in experiments in vitro, but not in vivo [38]. Microglia cultures have limited applications because of multiple changes happening with these cells under culture conditions [58]. OPCs are capable of producing neurons in vitro, but their ability to do so in the brain is debated (see Section 3.2). iPSCs differentiated into neurons in vitro did not closely resemble any known subtype of CNS or PNS neurons; neurons in iPSC-derived brain organoids were more similar to some reference neuronal subtypes, highlighting the role of organ structure (ECM and guiding molecules) in neuron development [322]. Indeed, more recently, a successful generation of a specific subtype of interneurons starting from the ESC-derived glial precursor cells was demonstrated in a 3D spheroid culture environment; the iNs were transcriptomically highly similar to endogenous chandelier PV+ interneurons [438]. However, even with 3D culturing techniques, subtle differences in microenvironment between brain regions (which may be crucial for glia-to-neuron conversion, as discussed above) may be very difficult to imitate in vitro. Thus, experiments in vivo are a more reliable approach when disease models and putative treatments are considered. Still, some insights from animal models may not be useful for understanding processes in the human brain due to species-specific differences and some techniques used in experiments with rodents being undesirable for clinical applications. For example, adult neurogenesis in different mammalian species happens in different brain regions [154,171,173,439,440], with important differences even between closely related species such as rats and mice [153,441]. There are significant differences between expressed genes and dominant signaling pathways in mouse and human OPCs [442]. Human and mouse astrocytes have different degrees of susceptibility to oxidative stress and different responses to hypoxic and inflammatory conditions [443]. Finally, in experiments with rodents, viral vectors are usually injected directly into the brain, while this method of administration is not preferred when designing treatments for people. Brulet et al. demonstrated less effective astrocyte-to-neuron transformation when the vector was delivered systemically, even though the effective infection of astrocytes was confirmed. They suggest that the direct injection of a virus into the brain, as used in other studies, causes reactive gliosis by itself, and that reactive astrocytes located around the injection point are more responsive to transdifferentiation factors, which may skew the interpretation of results [28].

## 7. Conclusions

- Glia-to-neuron transdifferentiation therapy has the potential to help some patients with epilepsy, but if it is eventually approved for clinical use, its efficiency should be estimated on a case-to-case basis.
- Using indirect evidence (estimations of injury-related neurogenesis and transdifferentiation efficiency in animal models of diverse neuropathologies, as well as some results with resected epileptic brain tissue), it may be speculated that this therapy may be the most effective in very young patients, in cases of epilepsy starting shortly after TBI or stroke, and in cases where a hemorrhage or a glial scar are observed. It is possible that transdifferentiation therapy may also prove useful in cases with hippocampal sclerosis.
- Some anti-seizure drugs may improve the result of neuroregeneration therapies in epilepsy by promoting the generation of new neurons or increasing the survival of transitioning cells, new neurons or NSCs.
- It seems logical that the target identity of iNs in epilepsy is GABAergic interneurons. However, thorough testing strategies in animals are necessary to make sure these interneurons are properly integrated in existing circuits and would not participate in GABAergic ictogenic mechanisms.
- In most brain regions, the microenvironment is not favorable for neurogenesis, which also affects the transdifferentiation efficiency. The epileptic focus is usually located in the hippocampus or the cortex. Out of these two regions, the hippocampus may have a more neurogenic-permissive microenvironment since it contains a neurogenic niche. Extra steps and additional factors in the transdifferentiation protocol may be necessary when the target region is the cortex.
- Multiple factors complicate the estimation of cell type identity and transdifferentiation efficiency in experiments with animals. False positive results when preexisting neurons are labeled as iNs are possible; in addition, astrocytes as the starting cell type may be confused with NSCs. To overcome this, a strict lineage tracing and a careful choice of immunolabeling markers are advisable.
- Some methods aimed at producing new neurons in vivo (transdifferentiation of resident glia or NSC grafting) have been shown to have additional beneficial effects, including the protection of existing neurons, the normalization of synaptic plasticity and the prevention of neural circuit remodeling by abnormal neurogenesis, which is particularly important for epilepsy.
- In connection to the last two conclusions, there are reasons to believe that in some cases, the observed beneficial effects of transdifferentiation factors should not be attributed to the generation of iNs from glia, and must have other explanations. A situation wherein a working treatment is discovered by experiments based on wrong assumptions is still possible; however, more fundamental studies are warranted if there are doubts about the mechanisms of treatment.

Our current knowledge about changes in glial cells associated with epilepsy and about glia-to-neuron transformation methods provides an ample theoretical basis for developing strategies of treating epilepsy by cell transdifferentiation. However, so far there are not enough experimental papers wherein the transdifferentiation approach to neuroregeneration was used specifically in epilepsy models. This area of study is now at its early stage; nevertheless, the existing works provide encouraging results, and further research is warranted. The most urgent questions that may be answered by experiments in animal models include the definition of the time period during the progression of epilepsy in which transdifferentiation therapy may be the most efficient; the comparison of outcomes in models with or without hippocampal sclerosis; the estimation of effects of anti-seizure or anti-inflammation drugs used together with transformation factors. With epilepsy studies, there is also a unique opportunity to use live human brain tissue for research. Experiments with resected epileptic brain samples may elucidate which epilepsy etiologies and/or comorbidities make glial cells more susceptible for reprogramming interventions, which is the most important question for moving from preclinical to clinical trials.

## Figures and Tables

**Table 2:** Comparison of different reprogramming factors described in the studies cited in Section 4 and Section 5.

Reprogramming Factor ^1^	Physiological Role of the Factor	Starting Cell Types (In Vitro and In Vivo Experiments)	Resulting Cell Types (In Vitro and In Vivo Experiments)	Animal Models of Neuropathology Used in Transdifferentiation Experiments In Vivo	In Vivo Transformation Efficiency Range ^2^	Section Number
PAX6	Patterning the telencephalon in embryogenesis	Astrocytes	Neurons (subtype not specified)	-	-	Section 4.2
ASCL1	Differentiation of the GABAergic lineage during development and maintenance of adult NSC pool	Fibroblasts, astrocytes, OPCs	Neurons: glutamatergic, GABAergic (including specifically PV+), dopaminergic	PD, TBI, stroke, epilepsy	20–94%	Section 4.3 and Section 5
NeuroD1	Glutamatergic neuron differentiation in developing and adult brain	Astrocytes (including resting astrocytes), OPCs, microglia	Neurons: glutamatergic (including specifically TLX3+ spinal cord neurons), GABAergic (including specifically PV+, nNOS+, SST+, calretinin+, calbindin+, cholecystokinin+)	SCI, TBI, stroke, neonatal hypoxic–ischemic brain injury, epilepsy	0–96%	Section 4.4 and Section 5
SOX2	Self-renewal of NSCs (When SOX2 is used for neuronal transdifferentiation, additional factors are required)	Astrocytes, OPCs	Neural progenitors (ASCL1+, later DCX+); DCX+ immature neurons; calretinin+ striatal GABAergic neurons (when SOX2 is used together with neurotrophic factors or valproic acid)	SCI, TBI	-	Section 4.5
Ngn2	Commitment of progenitor cells to neuronal lineage in the developing nervous system	ESCs, iPSCs, astrocytes, OPCs, microglial lineage cells derived from ESCs	Neurons: glutamatergic (including specifically BRN2+/CUX1+/FOXG1+ layer 2/3 cortical neurons), cholinergic; PNS types	SCI, TBI	0–85%	Section 4.6
DLX2	Forebrain GABAergic neuron differentiation	Astrocytes, OPCs, NSCs	GABAergic neurons, including specifically striatal types (calretinin+, PV+, DARPP32+/CTIP2+ medium spiny neurons) and spinal cord PAX2+	HD, SCI, stroke, epilepsy	3–92%	Section 4.7 and Section 5
Notch1 downregulation	Notch signaling in NSCs prevents their differentiation into neuronal lineage	Astrocytes	Neuroblasts, GABAergic neurons (including specifically striatal nNOS+ and LGE_MEIS2/PAX6 embryonic class)	SCI, TBI	-	Section 4.8
PTBP1 downregulation	PTBP1 maintains alternative splicing patterns specific for non-neuronal cells and prevents neuronal differentiation by inhibiting PTBP2 and miR-124	Established cell lines (see Table 1), fibroblasts, astrocytes, NSCs	Neurons: glutamatergic, dopaminergic, ChAT+ motor	Stroke, PD, TBI, SCI	23–80%	Section 4.9
miR-124	Organization of TF networks involved in neuroprotection and neuronal subtype differentiation, coordination of metabolic changes during neurogenesis, inhibition of PTBP1	Astrocytes	Immature neurons (ASCL1+/TBR2+); glutamatergic and GABAergic neurons (when miR-124 is used together with ISX9)	TBI	72–75%	Section 4.10

^1^ The table includes studies where the specified factor was used in combination with other factors. ^2^ Efficiency numbers are taken only from papers that specify them as the ratio of NeuN+, FP+ cell number to FP+ cell number, where FP is the fluorescent protein used as the marker of transduction (similar to Figure 3 and Supplementary Table S1). For different factors, the range was estimated with different numbers of studies.

**Table 3:** Differences in methods used in studies reporting successful or unsuccessful astrocyte-to-neuron or microglia-to-neuron transdifferentiation in vivo with NeuroD1 overexpression or PTBP1 downregulation.

**NeuroD1 Overexpression (In Vivo Studies Cited in Section 4.4)**		
Studies: 14; used astrocytes: 11; microglia: 3	Reported many iNs: 11	Reported no or very few iNs: 3
Used an AAV vector ^1^	6/11	0/3
Used a universal promoter ^2^	0/11	2/3
Used GFAP promoter for astrocytes	8/9	1/2
Used viral or genetic lineage tracing ^3^	9/11	3/3
Astrocyte fate tracing used GFAP promoter	6/9	1/2
**PTBP1 Downregulation (In Vivo Studies Cited in Section 4.9)**		
Studies: 10, all used astrocytes	Reported many iNs: 7	Reported no or very few iNs: 3
Used an AAV vector ^1^	5/7	2/3
Used a universal promoter ^2^	0/7	0/3
Used GFAP promoter	6/7	2/3
Used viral or genetic lineage tracing ^3^	2/7	2/3
Fate tracing used GFAP promoter	2/7	1/3

^1^ Studies using AAV serotypes with confirmed tropism for astrocytes are not counted. ^2^ Studies using a Cre-FLEX system with one cell type-specific and one universal promoter are not counted. ^3^ The use of Cre-FLEX for cell transduction with TFs (not exclusively for labeling) is counted as a form of lineage tracing. More detailed information (the exact names of vectors, promoters, transgenic strains used in each reference, as well as some details of additional experiments with OPCs and Müller glia) is presented in Supplementary Table S2.

## Data Availability

No new data were created or analyzed in this study. Data sharing is not applicable to this article.

## Supplementary Materials

The following supporting information can be downloaded at: https://www.mdpi.com/article/10.3390/ijms27209147/s1.

## Abbreviations

The following abbreviations are used in this manuscript:

A2B5	The number of the cell clone producing the antibody recognizing c-series gangliosides with three sialic acids
AAV	Adeno-associated virus
ABCA1	ATP Binding Cassette Subfamily A Member 1
AD	Alzheimer’s disease
ADK	Adenosine kinase
Aldh1l1	Aldehyde Dehydrogenase 1 Family Member L1
Aldoc	Aldolase C
ALN	ASCL1, LMX1A, and NURR1
AP	Action potential
APOE	Apolipoprotein E
AQP4	Aquaporin 4
Arx	Aristaless related homeobox
ASCL1	Achaete Scute Complex Like-1
ATP	Adenosine triphosphate
Aβ42	Amyloid-β protein (1–42)
BAC	Bacterial artificial chromosome
BAM	ASCL1, BRN2 and MYT1L
BBB	Blood–brain barrier
Bcl2	B cell leukemia/lymphoma 2
BDNF	Brain-derived neurotrophic factor
Best1	Bestrophin-1
BrdU	Bromodeoxyuridine
BRN2	Brain-Specific Homeobox/POU Domain Protein 2
Btg2	B-Cell Translocation Gene 2
C1q	Complement component 1q
C3	Complement component 3
CA1–4	Cornus ammonis 1–4
CAG	Hybrid promoter containing CMV enhancer and the promoter of the chicken β-actin gene
Calb2	Calbindin 2
CaMKII	Ca^2+^/calmodulin-dependent protein kinase II
Cas	CRISPR-associated protein
CC1	The number of the cell clone producing the antibody recognising adenomatosis polyposis coli protein
CCM	Cerebral cavernoma
CD11b	Cluster of differentiation molecule 11b (integrin subunit alpha M)
CD16	Cluster of differentiation molecule 16 (type III Fcγ receptor)
CD24	Cluster of differentiation molecule 24
CD68	Cluster of differentiation molecule 68
Cdk1	Cyclin-dependent kinase 1
ChAT	Choline acetyltransferase
CHX10	ceh-10 homeodomain-containing homolog
CldU	5′-Chloro-2′-deoxyuridine
CMV	Cytomegalovirus
CNPase	2′,3′-Cyclic-nucleotide 3′-phosphodiesterase
CNS	Central nervous system
COX	Cyclooxygenase
CPOX	Coproporphyrinogen oxidase
CreER	Cre recombinase fused to a mutated ligand-binding domain of the estrogen receptor
Cre-FLEX	Cre-mediated flip-excision
CRISPR	Clustered regularly interspaced short palindromic repeats
CSF	Cerebrospinal fluid
CSPG4	Chondroitin sulfate proteoglycan 4
Cstb	Cystatin B
CTIP2	COUP-TF-interacting protein 2
CUX1	Cut Like Homeobox 1
Cx	Connexin
CXCL1	C-X-C motif chemokine ligand 1
CXCL10	C-X-C motif chemokine ligand 10
D2L	Dopamine receptor D2-like family
DAMPs	Damage-associated molecular patterns
DAPT	N-[N-(3,5-difluorophenacetyl)-l-alanyl]-S-phenylglycine t-butyl ester
DARPP32	Dopamine- and cAMP-regulated neuronal phosphoprotein
DAT	Dopamine transporter
DCX	Doublecortin
DDC	DOPA decarboxylase
DEG	Differentially expressed gene
DG	Dentate gyrus
Dll1	Delta Like Canonical Notch Ligand 1
DLX1	Distal-less homeobox 1
DLX2	Distal-less homeobox 2
DLX5	Distal-less homeobox 5
DNMT3A	DNA methyltransferase 3 alpha
dpi	Days post-infection
DsRed	*Discosoma* sp. red fluorescent protein
E	Embryonic day
EAAC1	Excitatory amino acid carrier-1
EAAT1	Excitatory amino acid transporter 1
EAAT2	Excitatory amino acid transporter 2
ECM	Extracellular matrix
EdU	5-Ethynyl-2′-deoxyuridine
EF1α	Elongation factor 1 alpha
EGF	Epidermal growth factor
En1	Engrailed Homeobox 1
En2	Engrailed Homeobox 2
EPSC	Excitatory postsynaptic current
EPSP	Excitatory postsynaptic potential
ERBB2	Erb-B2 Receptor Tyrosine Kinase 2
ERK	Extracellular-signal-regulated kinase
ESC	Embryonic stem cell
ET1	Endothelin-1
FACS	Fluorescence-activated cell sorting
FBS	Fetal bovine serum
FGF	Fibroblast growth factor
FGF-2	Fibroblast growth factor 2
FOXA2	Forkhead Box A2
FOXG1	Forkhead Box G1
FP	Fluorescent protein
GABA	Gamma-aminobutyric acid
GABA_A_R	Type A gamma-aminobutyric acid receptor
GAD	Glutamate decarboxylase
GAD65	Glutamate decarboxylase, 65 kDa
GAD67	Glutamate decarboxylase, 67 kDa
GADD45B	Growth arrest and DNA-damage-inducible 45 beta
GAERS	Genetic Absence Epilepsy Rats from Strasbourg
GAT3	GABA transporter type 3
Gbx2	Gastrulation Brain Homeobox 2
GDNF	Glial cell derived neurotrophic factor
GFAP	Glial fibrillary acidic protein
GFP	Green fluorescent protein
GJA1	Gap Junction Protein Alpha 1
GLAST1	GLutamate ASpartate Transporter 1
GLS	Glutaminase
GLT-1	Glutamate transporter 1
GlyT2	Glycine transporter 2
GO	Gene Ontology
GPM6A	Glycoprotein M6A
GS	Glutamine synthetase
HCAR1	Hydroxycarboxylic acid receptor 1
HD	Huntington’s disease
HeLa	Cell line derived from cervical cancer cells of Henrietta Lacks
Hes5	Hes Family BHLH Transcription Factor 5
Hey1	Hes Related Family BHLH Transcription Factor With YRPW Motif 1
HIBI	Hypoxic-ischemic brain injury
HLA-DR	Human Leukocyte Antigen—DR isotype
HMGB1	High mobility group box 1
HNA	Human nuclear antigen
HS	Hippocampal sclerosis
Hsp90	Heat shock protein 90
HS-TLE	Temporal lobe epilepsy with hippocampal sclerosis
i.c.v.	Intracerebroventricularly
i.v.	Intravenous
iANBs	Induced adult neuroblasts
IB4	Isolectin B4
IBA1	Ionized calcium binding adaptor molecule-1
IBE	International Bureau for Epilepsy
iC3b	Inactive complement component 3b
ICM	Intracranial meningioma
IFNβ	Interferon beta
IFNγ	Interferon gamma
IGF-1	Insulin-like growth factor 1
IKK	I Kappa B Kinase
IL1α	Interleukin 1 alpha
IL1β	Interleukin 1 beta
IL4	Interleukin 4
IL6	Interleukin 6
ILAE	International League Against Epilepsy
iN	Induced neuron
iPCS	Induced pluripotent stem cell
IPH	Intraparenchymal hemorrhage
IPI	Initial precipitating injury
IPSC	Inhibitory postsynaptic current
Islet1	ISL LIM Homeobox 1
ITGAV	Integrin Subunit Alpha V
JAK	Janus kinase
JNK	c-Jun N-terminal kinase
KA	Kainic acid
KCNJ10	Potassium inwardly rectifying channel subfamily J member 1
Ki67	Marker of proliferation Kiel 67 (protein)
LARA	Lipid-accumulated reactive astrocyte
LDH	Lactate dehydrogenase
LDHA	Lactate dehydrogenase subunit A
LHX6	LIM Homeobox 6
LMX1A	LIM homeobox transcription factor 1 alpha
LRP1	LDL Receptor Related Protein 1
LV	Lentivirus
MAP2	Microtubule-associated protein 2
MAPK	Mitogen-activated protein kinase
MASH1	Mammalian Achaete-Scute Homolog-1
MBP	Myelin basic protein
mCherry	Monomeric Cherry fluorescent protein
MCT1	Monocarboxylate transporter 1
MEA	Medial entorhinal area
mGluR	Metabotropic glutamate receptor
Mki67	Marker of proliferation Kiel 67 (gene)
MMLV	Moloney murine leukemia virus
MMP	Matrix metalloproteinase
mpi	Months post-infection
MTLE	Mesial temporal lobe epilepsy
Myt1l	Myelin transcription factor 1 like
N2A	Neuro-2a mouse neuroblastoma cell line
NANOG	Nanog homeobox
Nestin-TK	Nestin-δ-HSV-thymidine kinase-EGFP
NeuN	Neuron-specific nuclear protein
NF-kB	Nuclear factor kappa-light-chain-enhancer of activated B cells
NG2	Nerve/glial-antigen 2 (chondroitin sulfate proteoglycan 4)
Ngn1–3	Neurogenin 1–3
NMDAR	*N*-methyl-D-aspartate receptor
nNOS	Neuronal nitric oxide synthase
Nog	Noggin
NOTCH2	Notch receptor 2
NPC	Neural progenitor cell
NPY	Neuropeptide Y
NR2	*N*-methyl-D-aspartate receptor subunit R2
NSC	Neural stem cell
NT3	Neurotrophin 3
Nurr	Nuclear receptor-related 1 protein
O-2A	Oligodendrocyte-type-2 astrocyte progenitor
O4	Oligodendrocyte marker sulfated galactocerebroside O4
Olig1	Oligodendrocyte transcription factor 1
Olig2	Oligodendrocyte transcription factor 2
OPC	Oligodendrocyte precursor cell
P	Postnatal day
P2X7	P2X purinoceptor 7
P2YR1	P2Y purinoceptor 1
p38	p38 mitogen-activated protein kinase
p53	transformation related protein 53
PAG	Phosphate-activated glutaminase
PAP	Perisynaptic astrocyte process
PAX2	Paired Box 2
PAX3	Paired Box 3
PAX6	Paired Box 6
PAX7	Paired Box 7
PD	Parkinson’s disease
PDGFRα	Alpha receptor for platelet-derived growth factor
Plp	Proteolipid protein 1
PNN	Perineuronal net
PNS	Peripheral nervous system
Prdx2	Peroxiredoxin 2
PROX1	Prospero homeobox protein 1
PRPH	Peripherin
PTB-ASO	Antisense oligonucleotide targeting Ptbp1 mRNA
PTBP1	Polypyrimidine Tract Binding Protein 1
Pten	Phosphatase and tensin homolog
PTZ	Pentylenetetrazole
PV	Parvalbumin
QA	Quinolinic acid
Rad-NSCs	Reactive astrocyte-derived neural stem cells
Rbfox3	RNA Binding Fox-1 Homolog 3
Rbpj	Recombination signal binding protein for immunoglobulin kappa J region
RC2	Radial glial cell marker-2 (a specific form of nestin)
RG	Radial glia
RGLC	Radial glia-like cell
ROS	Reactive oxygen species
ROSA26	Reverse Orientation Splice Acceptor 26 (a locus in mouse genome commonly used for insertions)
RT-qPCR	Reverse transcription quantitative polymerase chain reaction
S100β	S100 calcium-binding protein B
SATB2	Special AT-rich sequence-binding protein 2
SC121	The number of the mouse monoclonal antibody labeling human cytoplasm
SCI	Spinal cord injury
SCL1A3	Solute Carrier Family 1 Member 3
SCO	Subcommissural organ
scRNA-Seq	Single-cell RNA sequencing
SE	Status epilepticus
SEZ	Subependymal zone
Sfxn5	Sideroflexin 5
sgRNA	Single guide RNA
SGZ	Subgranular zone
SHH	Sonic hedgehog
shPTB	shRNA targeting Ptbp1 mRNA
shRNA	Short hairpin RNA
SLC7A11	Solute carrier family 7 member 11
snRNA-Seq	Single-nucleus RNA sequencing
SOX10	SRY-Box Transcription Factor 10
SOX11	SRY-Box Transcription Factor 11
SOX2	SRY-Box Transcription Factor 2
SOX4	SRY-Box Transcription Factor 4
Srrm4	Serine/Arginine Repetitive Matrix 4
ssAAV	Single-stranded adeno-associated virus
SST	Somatostatin
STAT3	Signal transducer and activator of transcription 3
STR-nbl	Neuroblasts generated by striatal astrocytes
SVZ	Subventricular zone
TAP	Transit-amplifying progenitor
TBI	Traumatic brain injury
TBR1	T-box brain transcription factor 1
TBR2	T-box brain transcription factor 2
tdRFP	Tandem-dimer red fluorescent protein
tdT	Tandem dimer Tomato fluorescent protein
TF	Transcription factor
TGFβ	Transforming growth factor beta
TGFβR	Transforming growth factor beta receptor
TH	Tyrosine hydroxylase
TLE	Temporal lobe epilepsy
TLX3	T Cell Leukemia Homeobox 3
tMCAO	Transient middle cerebral artery occlusion
TNFα	Tumor necrosis factor alpha
*t*-NMDAR	Triheteromeric N-methyl-D-aspartate receptor
Tubb3	Beta-tubulin class III
vg/ml	Number of viral genomes per 1 mL of solution
VGAT	Vesicular GABA transporter
VGlut1	Vesicular glutamate transporter 1
VGlut2	Vesicular glutamate transporter 2
VPA	Valproic acid
V-SVZ	Ventricular-subventricular zone
WAG/Rij	Wistar Albino Glaxo from Rijswijk
Wnt	Wingless-related integration site
wpi	Weeks post-infection
WT	Wild-type
YAP1	Yes1 Associated Transcriptional Regulator
YFP	Yellow fluorescent protein
Z/EG	Reporter system expressing *lacZ* before Cre excision and *EGFP* after Cre excision
Zeb2	Zinc finger E-box binding homeobox 2
ZFP36L1	ZFP36 Like 1 Zinc Finger CCCH-Type
